# An Integrative Approach to Inferring Gene Regulatory Module Networks

**DOI:** 10.1371/journal.pone.0052836

**Published:** 2012-12-20

**Authors:** Michael Baitaluk, Sergey Kozhenkov, Julia Ponomarenko

**Affiliations:** 1 San Diego Supercomputer Center, University of California San Diego, La Jolla, California, United States of America; 2 Skaggs School of Pharmacy and Pharmaceutical Sciences, University of California San Diego, La Jolla, California, United States of America; American University in Cairo, Egypt

## Abstract

**Background:**

Gene regulatory networks (GRNs) provide insight into the mechanisms of differential gene expression at a system level. However, the methods for inference, functional analysis and visualization of gene regulatory modules and GRNs require the user to collect heterogeneous data from many sources using numerous bioinformatics tools. This makes the analysis expensive and time-consuming.

**Results:**

In this work, the BiologicalNetworks application–the data integration and network based research environment–was extended with tools for inference and analysis of gene regulatory modules and networks. The backend database of the application integrates public data on gene expression, pathways, transcription factor binding sites, gene and protein sequences, and functional annotations. Thus, all data essential for the gene regulation analysis can be mined publicly. In addition, the user’s data can either be integrated in the database and become public, or kept private within the application. The capabilities to analyze multiple gene expression experiments are also provided.

**Conclusion:**

The generated modular networks, regulatory modules and binding sites can be visualized and further analyzed within this same application. The developed tools were applied to the mouse model of asthma and the OCT4 regulatory network in embryonic stem cells. Developed methods and data are available through the Java application from BiologicalNetworks program at http://www.biologicalnetworks.org.

## Introduction

One of the goals of systems biology is to infer gene regulatory networks (GRNs) from experimental data. GRNs describe and visualize dependencies between proteins, transcription factors (TFs) and their target genes. GRNs has proven to be a useful tool in describing complex transcriptional programs in development [1], hematopoiesis [2], and global regulatory programs in *S. cerevisiae* [3] and bacteria [4]. GRNs can be built from the modules of co-expressed genes, assuming that TFs and other regulators are co-expressed with the genes they regulate [3], [4], [5], [6]. An assumption that only regulated genes should be co-expressed can also be applied [7], [8]. Several methods have been developed to address the problem both at the level of cis-regulatory modules and global network (see [1] and references within). Some methods, such as the Signature Method [9], Stochastic LeMoNe [10] and Inferelator [11] use only expression data; while others, for example, GRAM [5], SPARC [12], DISTILLER [13], GPS [14], and others [15] use additional experimental data and thus fall into the category of integrative methods. Integrative methods might take into account known protein-protein interactions [16], data on TF affinity to various DNA sequences [17], *in vitro*-obtained DNA-binding specificities [12], sequence data on experimentally determined TF binding sites from genome-wide experiments [5], [13] or *de novo* DNA motifs [3], even searching for them concurrently with the bi-clustering genes and conditions of the expression data [18].

Integrative methods are the most attractive and promising for inferring gene regulatory modules and global networks because they take into account a wealth of biological data [4]. However, these methods challenge the user to collect heterogeneous data from many sources and to use numerous bioinformatics tools. The necessity of using different tools for visualization and module functional analysis further complicates the analysis, making it expensive, irreproducible, and time consuming. For example, to identify modules of co-regulated genes involved in response to asthma [19], the authors had to extract data from fifteen different databases, map the human genes to those in mouse, infer the modules using Genomica [3], and finally visualize and analyze the generated network and modules using the Ingenuity software.

The goal of this presented work was to develop a resource that simplifies and streamlines the regulatory network inference and analysis. The resource relies on the database IntegromeDB, which integrates public data on gene expression, pathways, gene and protein sequences from multiple species, and contains a comprehensive collection of public data on TF binding sites and gene regulatory sequences [20]. This database is accessible within the BiologicalNetworks application that has been developed by the authors to provide integrative analysis and visualization of networks, microarrays, and sequences [21], [22]. In this work, BiologicalNetworks was extended with tools for inferring and analysis of gene regulatory modules and networks. The implemented module inference method is unsupervised and integrative. The method is designed to be query-driven, which can be applied for inferring global networks as well. Following the recent trend [7], [8], TFs and regulators are assumed to not necessarily be co-expressed with the target genes. All data required for regulatory modules discovery are automatically mined during the module network inference. Additional data to mine can be integrated in the database by the user at www.integromedb.org/integration.jsp or used directly in the application.

BiologicalNetworks allows a researcher, starting with the list of genes or TFs and gene expression experiments, to select homologous TFs/genes, to select sources of data on known or/and predicted TF binding sites from the integrated databases, to build and visualize regulatory modules and network, and to explore them synchronically with expression data, protein-protein interactions, canonical pathways, and sequences of genes and regulatory sites. Similar to other available software for inferring regulatory modules and networks, this program allows users to upload their own data on TF-gene pairs and protein-protein interactions, as well as work with their own microarray experiments. The usability of the presented tools is demonstrated in two case studies.

## Methods

The tools described in this work are implemented within the BiologicalNetworks application. The modules, search, analysis and visualization capabilities are described in detail in the authors’ earlier works [21], [22], [23].

### IntegromeDB Database

The BiologicalNetworks application’s backend database is IntegromeDB [20] that integrates public data on gene expression, pathways, gene and protein sequences from multiple species and contains a comprehensive collection of public data on TF binding sites and gene regulatory sequences. Thus, all data required for regulatory modules discovery are automatically mined during the module network inference.

Genomic regulatory sequences, such as TF binding sites, are integrated with meta-graph (e.g., molecular interactions) and experimental data (e.g., microarray gene expression, etc.) in the backend database so that the genomic sequence intervals, represented as a Relational Interval (RI)-tree structures, are assigned to meta-graph objects. (RI)-trees are used for navigation through sequences (scroll upstream/downstream, GetNext gene/operon/chromosome, etc.) and annotation of multiple overlapping sequences. Internal enumerations in the integrated databases–for example, TRANSFAC [24], which provides localization of regulatory regions in respect to the transcription start site–are recalculated to correspond to global genome positions. All databases listed in the category *Transcriptional regulator sites and transcription factors* of the NAR Database depository [25] have been integrated in IntegromeDB. Among them are databases collecting only curated binding sites, e.g., TRANSFAC and ORegAnno [26], as well as databases providing predicted binding sites, e.g., ECRbase [27] and GenomeTraFaC [28].

### Workflow of Regulatory Modules Inference

The procedure of data mining and building modules in BiologicalNetworks consists of eight steps that are described in this section. The provided implementation of a network inference is query-driven; that is, to obtain the modules and a network, the user has to specify the list of genes or TFs (Step 1 below). The BiologicalNetworks’ *Build Transcription Regulatory Network Wizard* guides the user through these steps. The screen shots of BiologicalNetworks at each step are shown in **files S1 and S3**.

#### Step 1. Specify genes and TFs

To proceed with the analysis, the user has to specify genes/TFs or any other IDs because BiologicalNetworks recognizes virtually all publically available IDs or aliases for genes and proteins. Alternatively, genes/TFs can be obtained from the search and analysis of pathways in KEGG [29], REACTOME [30], NCI-Nature [31], and Human Cyc [32] or microarray experiments in GEO [33] and ArrayExpress [34] compendiums, which are integrated in IntegromeDB and available for search in BiologicalNetworks. To perform a search, the user specifies genes/TFs that can be typed directly in the search window of BiologicalNetworks, uploaded as a text file, or obtained through keyword or other types of search available in BiologicalNetworks. When the genes/TFs are selected, the user launches *Build Transcription Regulatory Network Wizard*, or simply the Wizard, which prompts the user to specify for each selected gene/TFs whether it should be considered a gene (transcription factors binding its regulatory regions will be searched), TF (its target genes will be considered), or both.

#### Step 2 (optional). Homology search

In the previous step, the user can either skip homology search or specify its stringency by selecting the minimum Blast bit-score as provided by the COGs database [35], from which the information about homologous was imported. Homologies across over 1100 organisms are supported. The Wizard allows the user to select genes/TFs for which targets and/or TF binding sites will be searched.

#### Step 3. Search target genes and binding sites

In this step, the user is prompted to specify data sources that provide information about TF binding sites and their target genes: either curated only, computed only, or both; specific data sources can be also selected/unselected. All databases listed in the category *Transcriptional regulator sites and transcription factors* of the NAR Database depository are available for selection. The region for searching TF binding sites can also be specified. On this step the user can also upload a data file(s) with TF-gene pairs obtained, for example, using genome-wide studies or motif-search algorithms. This data can be considered on its own, or together with data from selected databases.

#### Step 4. Select target genes and binding sites to build regulatory modules

When the search (Step 3 above) is complete, the user is encouraged to examine found genes, TFs, and binding sites and select only those that will be used to build a regulatory network (see **file S1**). Information about selected binding sites can also be saved in a file. As the integrated approach implemented in the IntegromeDB is purely automatic, erroneous sites and genes might be expected, meaning that BiologicalNetworks provides just the tool to examine found entries and manually narrow the search. In many cases, for the first exploration of data, the user doesn’t need to spend time on investigating each site and make a decision of whether to select it or not, because, for example, if an inferred module contains genes co-expressed with both the selected TFs and at least one of their targets (see the method of module inference below), the probability of obtaining such a module by chance is low. Further, the experiment can be repeated with a thorough selection of the sites and target genes. In this step, the user should select the species for which the experiments will be searched and for which the network will be built; by default, the species specified on Step 1 is chosen. All target genes and TFs in other species will be matched to homologous TFs/genes in the selected species.

With this tool, BiologicalNetworks provides unprecedented opportunity to simultaneously access and extract information from all databases listed in the category *Transcriptional regulator sites and transcription factors* of the NAR Database depository. For example, one can start with a list of transcription factors, using either their names or IDs from any database integrated or linked to the databases integrated in BiologicalNetworks (e.g., UniProt or PDB IDs), and using the Wizard, search for the list of target genes and binding sites. Note, however, that if the sequence of the site is specified in the database, the tool checks if this sequence matches to the sequence of the specified region of the gene. If there is no match, the site is not reported.

#### Step 5. Specify parameters to build regulatory modules

In this step, the user specifies the p-value (based on t-distribution) that will be used as a threshold for the significance of the Gene Ontology (GO) terms in selecting clusters of genes (the clustering method is described in the section *Building regulatory modules and the network* below). The user has also to specify whether the file(s) with the microarray experiment(s) will be provided by the user or multiple experiments will be selected from IntegromeDB and used to build the modules. In the former case, after the user specifies the file(s) located on the computer, Step 6 will be skipped. In the latter case, GEO and ArrayExpress will be searched for microarray experiments in which selected genes/TFs and their targets are strongly co-expressed; that is, the FDR (False Discovery Rate)-corrected on multiple experiments where the Pearson correlation coefficient is above 0.75. The experiments with a number of conditions (columns) more than 25 are not considered to avoid bias towards the experiments with too many conditions; likewise, experiments with less than 5 columns are also excluded to avoid bias towards samples with basic/control levels of expression that are usually present in every experiment. The number of experiments with more than 25 conditions did not exceed 5% of all available experiments. Still, the user can analyze an experiment of any size uploading the file.

For information on how to upload the user’s file, and for supported formats, see the tutorial at http://biologicalnetworks.org/tutorials/index.php#7. We recommend loading the microarray file in the system before opening the Wizard to make sure that the system recognizes the file format and opens the file properly, since the file will be loaded only after the Wizard finishes its work. As we are still in the process of obtaining statistics on the number of expression data points for human/mouse that can be processed at specific allocation of RAM, we give the user warning if the PC’s RAM might not be able to handle the selected amount of experiments. At 4 GB RAM we recommend working with fewer than 200 samples, or 10–20 average-size microarray experiments. In the future, we will provide an option to run extensive calculations on the server side.

#### Step 6. Select experiment(s) to build regulatory modules: the matrix of multiple experiments

**Figure 1:**
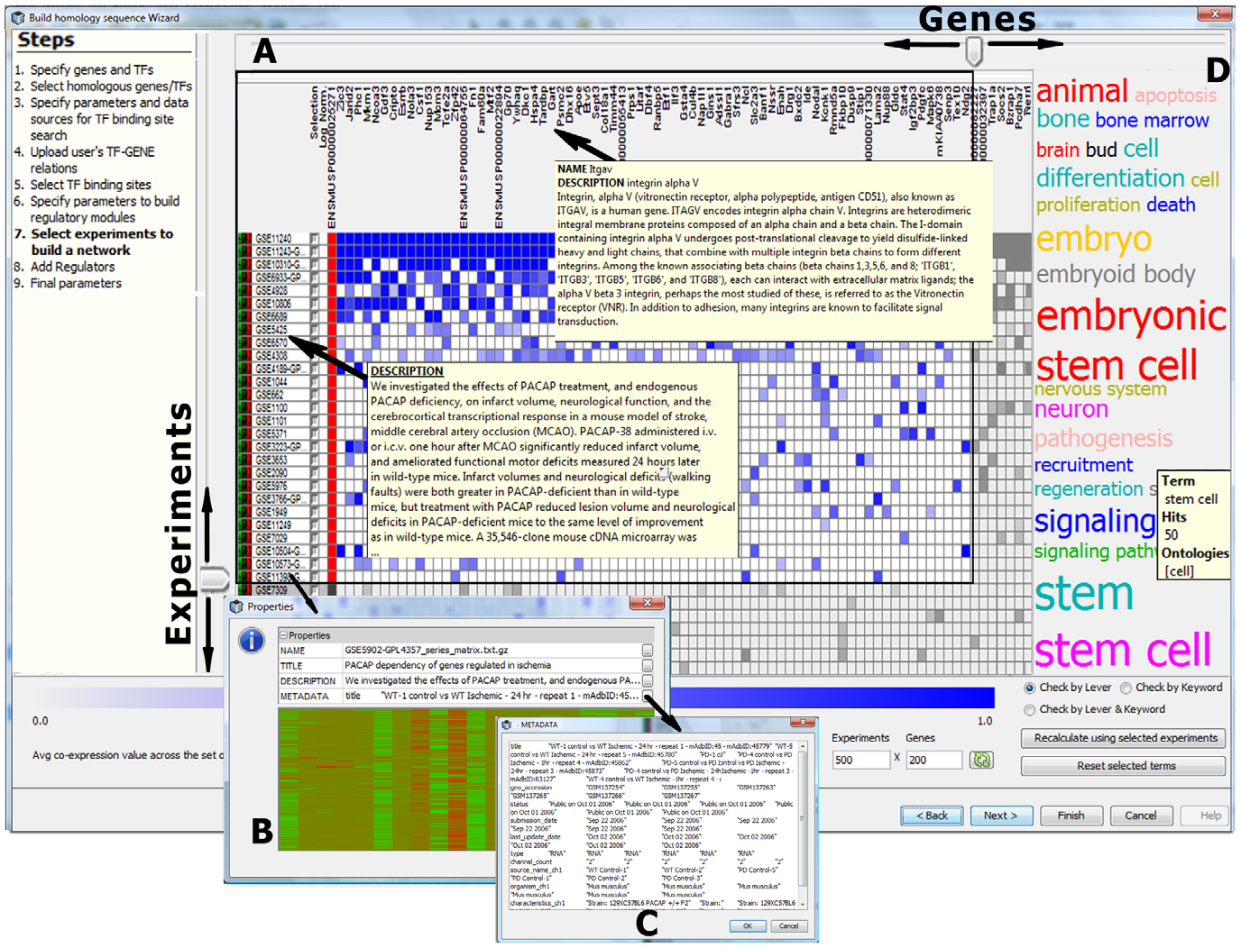
Screen-shot of the Multi-Experiment viewer (Use Case #1, Study 2). (**A**) The matrix represent the genes (in columns) co-expressed with the query gene(s) in microarray experiments (in rows). The brightness of blue of the matrix element corresponds to the co-expression value of the gene in an experiment (Eq. 4). The genes and experiments are sorted by average Z-values of genes (Eqs. 1–3). The vertical and horizontal levers allow selecting the highest ranked genes and experiments for building regulatory modules (the selection is shown in a black square). Hovering over the genes and experiments brings up their short description. (**B–C**) Clicking on the experiment ID brings up the experiment properties and visualization of the expression data. (**D**) A word cloud that characterizes the found set of experiments described by keywords (ontology terms representing cell types, tissues, diseases, biological processes, etc.). Clicking on the term in the cloud highlights respective experiments. The ‘Recalculate’ button allows the user to recalculate the matrix choosing only the experiments containing selected terms.


The selected experiments are represented in the Wizard as a matrix (**Fig. 1**)–the concept introduced earlier in a web resource MEM (Multi-Experiment Matrix) for gene expression similarity searches across datasets [36]. MEM outputs a ranked list of genes that are co-expressed with the query gene in the selected collection of experiments, which is platform-specific. Due to the integrated nature of IntegromeDB, our tool can deal with multiple genes and multiple collections of experiments, basically with all microarray data from GEO and ArrayExpress in which the query gene(s) can be found. And while MEM treats each probe for each gene in each microarray separately, we average data across multiple probes–if any–for each gene in each experiment, thus allowing the user to abstract from considering only one specific microarray platform. The matrix depicts the experiments (shown in rows) in which genes (shown in columns) were found to be co-expressed with the query gene(s). Co-expressed genes in the matrix are ranked based on averaged over *M* experiments average Z-values of the Pearson correlation coefficients of co-expression of the gene *x* with the query genes {*y_i_*} (*i = 1…N*, where *N* is the number of genes in the query list), which are calculated using Fisher’s Z-to-r transformation [37], [38]:
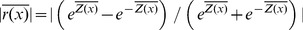
(1)

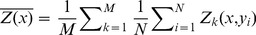
(2)where *Z_k_(x,y_i_)* is Fisher’s r-to-Z transformation of the Pearson correlation coefficient *r_k_(x,y_i_)* for the genes *x* and *y_i_* in a selected experiment *k (k = 1…M*, where *M* is the number of considered experiments):

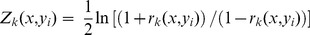
(3)

The experiments are ranked based on average Z-values (Eq. 2) averaged over all genes (columns) in the matrix.

The multi-experiment viewer (**Fig. 1**) was designed to allow the user selecting the best co-expressed genes and experiments that will be used for inferring regulatory modules. The user can select experiments in three different ways: by using a lever (to select the top ranked experiments), by keywords associated with the experiments, or both. Genes can be selected only by using the lever. For building the modules and the network, the selected genes will be considered in addition to previously selected TFs and their targets.

#### Step 7. Select regulators

At this step, the Wizard asks the user to select/unselect regulators which will be used in building the modules and the network, along with previously selected genes and TFs. Regulators are selected from IntegromeDB as annotated by the GO term or any database’s keyword *Transcriptional Regulation*. In contrast to the query gene/TFs, binding sites are not searched for regulators. Relationships between regulators and other genes/proteins are established based on available information about their physical interactions and co-citation.

#### Step 8. Final parameters setting

The final stage of the Wizard shows the final set of genes and proteins that the user has chosen for analysis and memory settings necessary for the run. In this step, the user can opt out of the visualization of the network, which takes additional time. Then, only regulatory modules will be inferred and shown. After the user clicks *Finish*, the Wizard finishes its work, and the nodes with their properties are retrieved from the database, the modules are inferred, and the network is generated. The time duration of this final step depends on the size of the microarray experiment(s), the number of considered genes and regulators, and the speed of the Internet connection. For example, on an Intel 2 GHZ processor and 6 GB RAM with 5 GB allocated for Java, runs for Studies 1 and 2 (see **files S1 and S3**) took about 15 minutes each.

### Building Regulatory Modules and the Network

In each selected experiment, the expression values for each gene are averaged across the gene probes for each sample. In cases where only one microarray experiment was selected or the user provided the experiment, each gene is described by a vector of expression values averaged over the gene probes. The similarity between each pair of genes, *x* and *y*, is calculated using either the Euclidian distance, Pearson correlation coefficient *r(x,y)*, or other distance; the similarity matrix is used as an input of the TEASE algorithm [39]. When more than one experiment is selected, the experiments cannot be uniformly normalized because data in different experiments may already be normalized in different scales and this information cannot be extracted from the experiment descriptions. Therefore, for multiple experiments, the TEASE’s input is the averages of transformed Pearson correlation coefficients computed for each pair of genes, *x* and *y*, across *M* experiments using Fisher’s Z-to-r transformation (Eqs. 1–3; where *y_i_* = *y* and *N = 1*).

The genes in the selected experiment(s) are hierarchically clustered with simultaneous GO term functional enrichment analysis as described in TEASE, prioritizing genes that are known to be regulated or have a binding site(s) for a respective TF(s) in the cluster. The module is inferred when at least one target gene for specified TFs/regulators can be found in the cluster with the TEASE’s p-value for at least one GO term below a specified threshold. The inferred modules are integrated in the regulatory network, which in turn is integrated with known (from literature as co-citation and public databases as direct or indirect evidence) protein-protein interactions.

It should be noted that TFs and regulators are treated both as genes and proteins since they can be regulated by other TFs and even self-regulated; therefore, on the network they are depicted twice as TFs/regulators and genes. Also, genes and proteins are treated as separate objects in BiologicalNetworks; thus, if two objects are known to be connected (through protein-gene, protein-protein interactions, co-expression, or co-citation) that connection between them is drawn on the network. However, to simplify the network representation, connections are not drawn among the genes in the modules. If the user is interested in seeing the known interactions among the genes and proteins within a module, these interactions can be drawn in BiologicalNetworks using BuildPathwayWizard (called by a right mouse-click).

### Integrative View of the Module Regulatory Network in BiologicalNetworks

**Figure 2:**
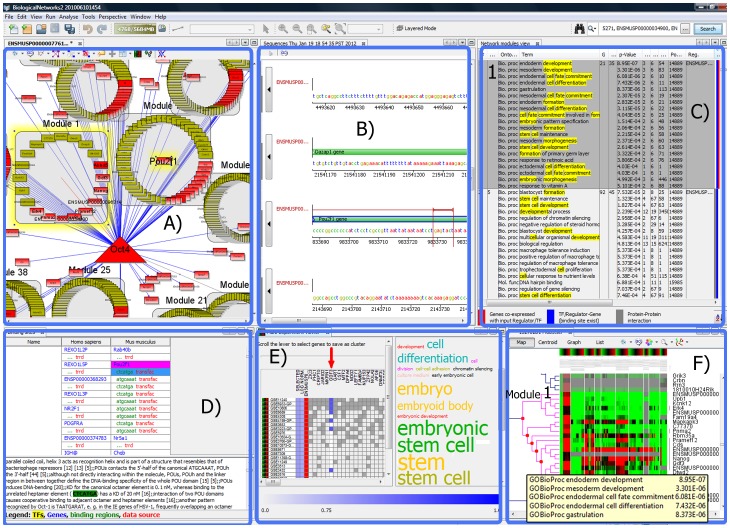
Integrative view of the OCT4 regulatory network (Use Case #1, Study 2). (**A**) Gene regulatory modular network of OCT4 transcription factor. Grey boxes represent the gene regulatory and co-expressed modules; rectangles represent the genes; red rectangles, the genes with known binding sites; a yellow triangle, the transcription factor; blue edges, TF-target gene relationships; red lines, co-expressed TF-gene pairs. The top module (shown in **C**), called ‘Module 1′, is highlighted. (**B**) GenomeBrowser window showing the sequences of the genes and TF binding sites. The OCT4 binding site for the selected in the network (**A**) *Pou2f1* gene is shown. (**C**) Module Table showing the gene modules, TFs, and functional annotation for each module with Fisher enrichment score (p-value) of GO terms. The top ‘Module 1′ is highlighted. (**D**) Table of TFs and target genes found in public databases. Gene *Pou2f1* (selected in **A**) is highlighted in magenta. (**E**) Multi-Experiment Viewer represents the matrix of genes (in columns) co-expressed with the query gene(s) in microarray experiments (in rows). (**F**) Microarray Gene Expression window showing the hit map and hierarchical tree of clustering data from selected experiments. Pointing out the mouse on the tree vertex shows the significant GO terms for the cluster; ‘Module 1′ is highlighted.

After the Wizard finishes inferring the modules, both the modules and the regulatory network can be seen in BiologicalNetworks (**Fig. 2**). In the final network (**Fig. 2A**), grey squares depict inferred modules, nodes represent genes/TFs/regulators, and edges represent interactions: regulatory (TF-gene) interactions established through the search for TF binding sites (Fig. 2A, blue edges), protein-protein interaction (Fig. 2A, grey edges), and co-expression in the selected experiments (Fig. 2A, red edges).

The windows in BiologicalNetworks are synchronized so that for a selected module in the network window, e.g., *Module 1* (**Fig. 2A**), functional terms are shown in the Module Table window (**Fig. 2C**), the heat-map and hierarchical tree of clustering the experiments are shown in the Microarray Gene Expression window (**Fig. 2F**), and the table of TF/gene binding sites and accompanying annotation are shown in **Figure 2D**. Also, selecting a specific node, for example, *Pou2f1* gene, in the network window (Fig. 2A), the user can see the information about its sequence and find TF binding sites (**Fig. 2D**) in the GenomeBrowser window (**Fig. 2B**).

**Figure 3:**
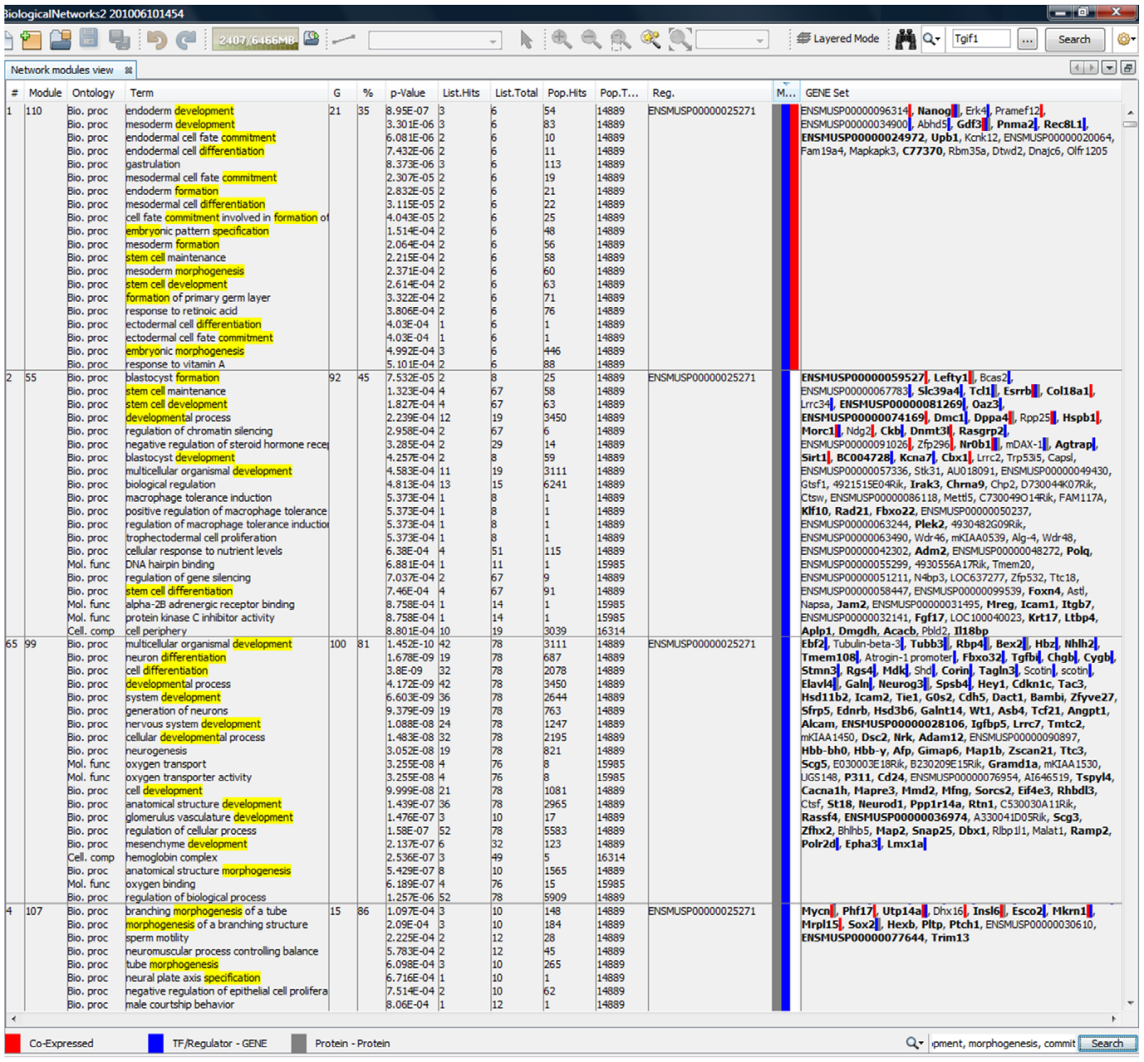
Screen-shot of BiologicalNetworks showing top OCT4 regulatory modules (Use Case #1, Study 2). The top module is marked in red as it contains OCT4 gene and the genes (marked in red) that are co-expressed with OCT4 in the selected in Study 2 experiments. It is also marked in grey as it contains genes (marked in grey) in which protein products are known to be involved in protein-protein interactions with OCT4 either in human or mouse. And it is marked in blue when it contains genes that have been selected in Study 2 as the mouse or human genes containing known or predicted OCT4 binding sites in the promoters. The ‘G’ column specifies the number of genes in each module. The ‘%’ column represents functional coherence of each module, measured as percentage of genes in the module covered by significant gene annotations (at a specified threshold on p-value). Each module is formed by a part of hierarchical clustering tree and thus represents a hierarchical tree with different terms assigned to different clusters. For each selected and shown GO term, we provide p-value, number of genes assigned to this GO term (the ‘List Hits’ column), number of genes in the tree clusters associated with this term (the ‘List Total’ column), and number of genes with this term among all mouse genes (the ‘Population Hits’ column) in the ontology (the ‘Population Total’ column). Genes with GO terms listed are shown in bold. Column ‘Regulators’ contains transcription factors and regulators (in this case OCT4 only) predicted to regulate a respective module. The search window on the right bottom allows the user to search genes and GO terms in the table.

The Modules Table presents a summary table of inferred modules (**Fig. 3**). For each module, the following information is provided: (i) significantly enriched GO terms; (ii) number of genes in the module (column ‘G’ in Fig. 3); (iii) functional/biological coherence of the module measured as the percentage of genes in the module covered by significant gene annotations (column ‘%’ in Fig. 3); and (iv) TFs and other regulators predicted to regulate the genes in the module, along with supporting evidence for each regulator/TFs. The supporting evidences are compiled from integrated data and can be as follows: *‘a regulator has known or predicted binding site in the gene(s) in the module’* (Fig. 3, red squares), *‘a regulator is co-expressed with the gene(s) in the module in the selected for the module inference experiments’* (Fig. 3, blue squares), and *‘a regulator is involved in protein-protein interactions with the product(s) of the genes in the module’* (Fig. 3, grey squares).

## Results and Discussion

Two use cases have been chosen to test the presented tools. The first use case studies the OCT4 regulatory network in mammalian embryonic stem (ES) cells, using microarrays and OCT4 binding data obtained in mouse ES cells [40], as well as data from public databases. The second case concerns a regulatory map of asthma in mouse built using microarray data published in [19]. The sequence of steps to reproduce the analysis described in this section is shown in **files S1, S2, and S3** and the Web tutorial. Note, however, that since the database and the ontology are regularly updated, the results of search and the resulting modules and networks obtained later might differ from those discussed here. In the multi-experiment matrix top ranked genes and experiments might also change with new experiments added in the database.

### Use Case #1: OCT4 Regulation in Mammalian ES Cells

Transcription factor OCT4, also known as POU5F1, was first isolated from mouse ES cells; it is a member of a large family of transcription factors that bind to the octameric DNA sequence ATGCAAAT [41]. OCT4 is a key factor of embryonic development, it controls self-renewal and pluripotency in ES cells [42], [43]. OCT4 regulation has been extensively studied in human [44] and mouse ES cells, using ChIP-PET [45], *Oct4* knockdowns [46] and other experimental [47] and computational [48] approaches. Here we demonstrate the results of the two following computational experiments. In Study 1, we considered only data from [40], specifically, time course data in mouse ZHBTc4 ES cells, in which the level of OCT4 expression was reduced in tetracycline-controllable manner, and OCT4 binding sites identified by Sharov and others as the most functionally relevant, using ChIP-PET data from [45]. In Study 2, an OCT4 regulatory network was built using public data on microarrays in mouse and human ES cells and OCT4 binding sites in the promoters of mouse and human genes, both experimentally identified and predicted as provided in public databases.

#### Study 1

In this study, for the module inference, the user’s data were used only. We started from searching the mouse OCT4 transcription factor in BiologicalNetworks (it was found under the name ENSMUSP00000025271) and then opened *Built Transcription Regulatory Network Wizard,* which guided us through the steps of building the regulatory modules. Microarray data was used as provided in Additional file 2 from [40] (see **file S4**). Also, we used OCT4 target genes from [45], selecting only those genes that had OCT4 PET-regions in [−10 kb;+1 kb] relative to the transcription start site (**file S5**). Regulators were not considered. The Pearson correlation coefficient was used as a measure of distance for the clustering of microarray data. At p-value of 1.0E-6 for GO term assignment, 26 modules were obtained; they were associated with such terms describing biological process as *Embryo development* and *Embryonic organ morphogenesis* (90 genes), *Cell cycle* (85 genes), *Tissue development* and *Intracellular signal transduction* (96 genes), *Primary metabolic process* (102 genes), *Nucleic acid metabolic process* (100 genes), *Positive regulation of biological process* (70 genes), *Biosynthetic process* (80 genes), *Translation* and *Ribosome biogenesis* (59 genes). Among 1919 genes assigned to the modules there were 486 genes which promoters bound OCT4 in ChIP-PET [45], including well-studied direct OCT4 targets, Sox2 and Nanog. At the less stringent p-value of 1.0E-4, 212 modules containing 10,003 genes were inferred, with 2063 of them with promoters binding OCT4 in ChIP-PET and 186 genes which protein products are known to bind OCT4. We looked closer at the top 70 modules ranked by the number of genes associated with significant GO terms and the genes which promoters bound OCT4 (**file S6**). They included 5371 genes, among which 140 were involved in protein-protein interactions with OCT4. The modules were associated with *Cell cycle, Tissue development, Signal transduction, Cell death, Apoptosis, Anatomical structure development, Metabolic process, Muscle fiber development, Nucleic acid metabolic process, Gene expression, Cellular response to hormone stimulus, Catabolic process, Regulation of DNA repair, Mesoderm morphogenesis, Formation of primary germ layer, Regulation of T-cell migration/chemotaxis/apoptosis, Nucleosome assembly, Chromosome segregation, Retina development, Eye morphogenesis, Organ morphogenesis, Nervous system development, Positive/negative regulation of fatty acid oxidation, Positive regulation of cell growth, Erythrocyte differentiation, Regulation of response to stress, Learning or memory, Female gamet generation, Embryonic organ development, Chordate embryonic development, Regulation of multicellular organismal development, Skeletal system development*, and other biological processes that are known to be associated with mammalian embryo development and were found for the putative OCT4 targets identified by Sharov and others, using the same experimental data and their own algorithm.

#### Study 2

This study was different from Study 1 as it relied only on public data and, along with regulatory modules (when the module of co-expressed genes contains at least one OCT4 target gene), the modules of only co-expressed genes were inferred. We started again with searching the mouse OCT4 transcription factor in BiologicalNetworks, searched for the OCT4 target genes in human and mouse in all databases providing experimentally identified and predicted OCT4 binding sites and looked for the sites located at [−10 kb;+1 kb] relative to the transcription start site. Microarray experiments were searched in IntegromeDB; and in the obtained matrix of gene-experiment pairs **(** **Fig.1** **)**, we selected the experiments that were associated with the keyword *Embryonic stem cell,* and then among them we selected the five top-ranked microarrays that contained 200 top genes co-expressed with OCT4 in human and mouse (the modules containing at least one of those genes will be attempted to be inferred as well). **File S1** provides the detailed instructions on how to repeat this run. At p-value of 1.0E-3, 118 modules containing 5780 genes were inferred (**file S7**). Among them, 61 modules included genes that contained known or predicted OCT binding sites reported in the databases. Of these 61 modules, 28 top modules included also genes that proteins are known to interact with OCT4 in either human or mouse; 16 of these 28 modules included genes that were co-expressed with OCT4 in the selected microarrays. Among these 28 modules (1871 genes), 16 modules (1289 genes) were associated with GO terms describing developmental processes and cell differentiation (two top modules are shown in **Fig. 3**); the rest modules were associated with biological processes *Lymphocyte differentiation* (3 modules), *RNA splicing”* and *Chromosomal segregation*, *Response to stimulus* and *Localization*, *Nucleic acid metabolism*, *Organ growth* and *Regulation of transcription*, *Response to chemical stimulus* and *ER-nucleus signaling pathway*, *Interaction with symbiont*, *Immune system process* and *Defense response* (2 modules), and *T cell activation*. This result demonstrates the power of the presented tools as a researcher with no preliminary data can infer the regulatory and co-expressed modules and build the gene regulatory network in a matter of a few hours. **Figure 2** shows the network built by the program and a screen-shot of the integrative view of discovered genes, experiments, and modules.

#### Comparison of the results in study 1 and study 2

The direct comparison of modules inferred in Study 1 and Study 2 is not strictly appropriate by the study design. Thus, in Study 1, only one microarray was used, OCT4 targets were obtained from ChIP-PET data, and the modules were required to contain at least one OCT4 target gene. While in Study 2, public data on OCT4 binding sites were used, along with public microarray data associated with the keyword *Embryonic stem cell* and containing genes co-expressed with OCT4; the modules were not required to contain OCT4 targets, they required to contain at least one gene co-expressed with OCT4. Also, Study 1 was restricted to a specific mouse cell line, while Study 2 included all available data for both mouse and human. These differences in the studies might explain the different number of inferred modules (212 in Study 1 and 118 in Study 2) and the low number of common genes obtained in Study 1 and Study 2, which was 1743, or 18% of all genes in the modules of Study 1 and 30%, of Study 2. The 100 top modules in each Study shared 167 significant GO terms (the hierarchy of GO terms was not considered), 18 of which were associated with development processes and included the GO terms *Embryonic Organ Development, Embryonic Organ Morphogenesis, Endoderm Formation, Anatomical Structure Morphogenesis, Blood Vessel Development, Positive Regulation Of Cell Proliferation*; 20 terms were related to metabolic processes; 20, to regulation and response; and other terms that are known to be associated with mammalian embryo development.

The results obtained in both studies support the hypothesis that the OCT4 may regulate transcription of many genes via mostly indirect binding to their promoters [40]. For example, it was shown that about 66% of the enriched (based on ChIP-on-ChIP) sequences did not contain OCT4 motifs, likely being indirect targets of OCT4 [48]. Among the genes in the modules inferred in Study 1 (at p-value of 1.0E-4) there were 61% of direct targets of OCT4 as identified in [40] and 35–52% of direct targets identified in the other three works, [48], [45], and [47]. It was not surprising that even so large a set of genes contained only half of direct OCT4 targets predicted by others as it was shown that an inter- and intra-species for ES and EC (embryonal carcinoma) cells comparison of putative OCT4 targets resulted in a rather small (from 10 to 25%) overlap of common targets [48]. This might be explained by the different platforms and analysis tools employed in the considered studies. Also, in Study 1 we intended to identify the genes that were co-expressed with the potential direct and indirect targets of OCT4 identified in ChIP-PET rather than searching for direct targets of OCT4. Among the genes in the modules inferred in Study 2 there were only 33% (1163) direct targets of OCT4 as identified in [40] from ChIP-PET data. This number is also not surprising as Study 2 was intended to infer along with regulatory modules (when the module of co-expressed genes contains at least one OCT4 target gene) the modules of only co-expressed genes. Our analysis was also influenced by the accuracy of GO annotation. Sharov and others considered only significantly down- and up-regulated genes and weighted the ChIP-binding sites based on the number of ChIP-PET ditags, the distance from TSS, and presence of CpG-rich regions. The absence of such data in pre-processing might have affected our results as well.

### Use Case #2: Gene Regulatory Module Network of Asthma

In this use case, to build a regulatory module network, we used published microarray data [19] and compared the modules inferred by BiologicalNetworks with the modules inferred by the authors using Module Networks software [3]. In Study 1, the input was all mouse genes listed in 61 modules inferred by Novershtern and others (the modules were inferred for 8086 mouse genes). To run the analysis on so many genes (6890 genes, as not all genes were included in the modules), 10 GB RAM was required. Therefore, we also conducted Study 2 on a much smaller set of 16 genes from one module. The sequence of steps to reproduce the analysis is shown in **file S3.**

#### Study 1

In this study, the genes included in the modules inferred by Novershtern and others were searched in BiologicalNetworks; TF binding sites in these genes were searched in all databases (for details see **file S3**). General transcriptional regulators were also considered. The Pearson correlation coefficient was used as a measure of distance for the clustering. In the result, 128 modules were inferred at p-value of 0.001 (see **file S8**). Among the top modules were the modules associated with the GO terms describing the immune system, leukocyte and lymphocyte regulation (p-value <1.0E-10; module #2, 108 genes); response to stimulus, inflammatory response and cytokine production (p-value <1.0E-10; module #9, 102 genes; module #51, 75 genes); immune response and leukocyte activation (p-value <1.0E-10; module #11, 116 genes); muscle and heart contraction and blood circulation (p-value <1.0E-10; module #24, 104 genes); signaling and cell communication (p-value <1.0E-9; module #25, 97 genes; module #29, 93 genes); oxidation-reduction process and respiratory chain (p-value <1.0E-20; module #36, 91 genes; p-value <1.0E-15; module #65, 54 genes; p-value <1.0E-10; module #74, 52 genes); and negative regulation of transcription, metabolic and biosynthetic processes (p-value <1.0E-8; module #6, 140 genes). These terms were found among those that were associated with the modules inferred by Novershtern and others. The gene *Il1rn* (interleukin 1 receptor antagonist), which is known to be associated with asthma in humans [49], was found in the module #58 that included 70 genes, was regulated among other by *Myc, Irf1, E2f1, Ccl2, Pax6, p53,* and was associated with the GO terms describing response to chemical stimulus, wounding, defense, nitric oxide mediated signal transduction, and CCR2 chemokine receptor binding (p-value <1.0E-6).

#### Study 2

**Figure 4:**
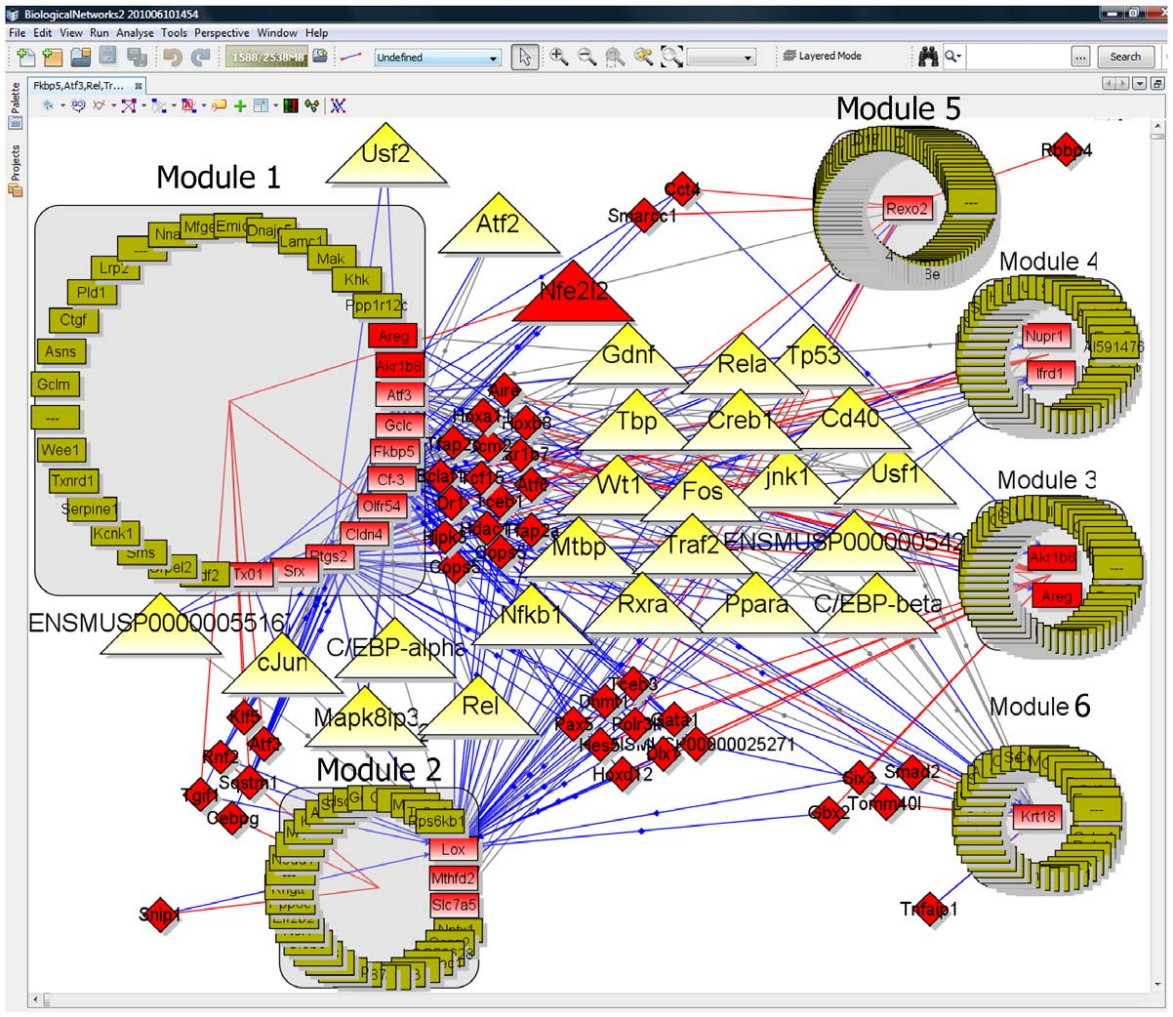
The modular network inferred for the genes from Module NC_622 (Use Case #2, Study 2). Grey boxes represent gene regulatory modules; rectangles, genes in the modules; red rectangles, genes from module NC_622; yellow triangles, transcription factors with known binding sites; red triangles, transcription factor that are co-expressed with the genes in the modules; red diamonds, regulators that are co-expressed with the genes in the modules; blue edges, TF-gene binding; red edges, co-expression relationships; grey edges, protein-protein interaction.

In this study, 16 genes from the Novershtern’s module #622, further called NC_622, were used as an input. Considering all found TFs and regulators potentially regulating these genes, 6 modules containing 349 genes were inferred in BiologicalNetworks (**file S9**). Module #1 of 39 genes contained 11 genes from Module NC_622 (**Fig. 4**; see also a screen-shot of integrative view in **file S3**) and was associated with responses to oxidative stress, hormone, endogenous and chemical stimuli. The genes in this module were potentially regulated by 25 TFs (when at least one TF binding sites were recorded in TRANSFAC, ORegAnno, Pazar, or TRRD databases) as these TFs co-expressed with the genes in the module; one of these TFs, GTF2h4, was common with potential regulators of module NC_622. Module #2 was associated with respiratory and lung development and metabolic process. Two of the rest modules were associated with the terms describing embryonic and organ development, morphogenesis and cell differentiation; this agrees with recent observations about a ‘developmental origin’ of asthma [50].

## Data Access

The described tools are available within the BiologicalNetworks software that can be download at www.biologicalnetworks.org. It is free for academic users.

### Tutorials and Demo

The ‘Regulation of OCT4 in mammalian ES cells’ and ‘Gene Regulatory Modular Network of asthma’ Projects, can be opened from the Welcome Page upon launching the BiologicalNetworks application, or from the BiologicalNetworks main page www.biologicalnetworks.org (see Driving Projects section).

### Data Used in the Use Cases

In Study 1 of Use Case #1, OCT4 regulatory genes identified by BiologicalNetworks were compared with the lists of genes provided in Additional files 4, 16, and 17 in [40], which in turn included the lists from [47] and [45]. For Study 1, microarray data and OCT4 binding sites were taken from Additional files 2 and 13 from [40]. Among the genes in Additional file 13, only genes that had OCT4 PET-regions in [−10 kb;+1 kb] relative to TSS were selected (3583 genes). The files in the txt-format that were used in Study 1 are provided as **files S4 and S5**. In Use Case #2, microarray data for analysis and regulatory modules were downloaded at http://www.jail.cs.huji.ac.il/~shefi. Specifically, to build the modules, we used a unified compendium of microarray data for 8086 genes and 52 samples. Modules 421 and 622 were considered in this work in detail.

### System Requirements

BiologicalNetworks is a Java application, requiring a minimum of 2 GB RAM dedicated to the Java Runtime environment and a stable Internet connection at all steps of the analysis. The quickness of the system response depends on RAM dedicated to Java. The use cases demonstrated in this work were performed on a PC with Windows 7 OS and 6 GB RAM, 5 GB of which was allocated for Java Runtime Environment (the RAM allocation can be specified upon installation of BiologicalNetworks).

## Supporting Information

File S1 **How to get started in BiologicalNetworks and step-by-step analysis for Use Case #1.**
(DOC)

File S2 **How to compare two lists of genes/proteins in BiologicalNetworks.**
(DOCX)

File S3 **Step-by-step analysis for Use Case #2.**
(DOCX)

File S4 **Microarray data used in Use Case #1, Study 1.**
(TXT)

File S5 **TF-gene pairs used in Use Case #1, Study 1.**
(TXT)

File S6 **Modules inferred in Use Case #1, Study 1.**
(TXT)

File S7 **Modules inferred in Use Case #1, Study 2.**
(TXT)

File S8 **Modules inferred in Use Case #2, Study 1.**
(TXT)

File S9 **Modules inferred in Use Case #2, Study 2.**
(TXT)

File S10 **List of genes in 70 top modules obtained in Use Case #1, Study1.**
(TXT)

File S11 **List of 420 genes (OCT4 targets) from Sharov’s Add. File 17** [**45**] **.**
(TXT)
