# Supplementary material for: An Integrative Approach to Inferring Gene Regulatory Module Networks
Source: PLoS One. 2012 Dec 20;7(12):e52836. doi: 10.1371/journal.pone.0052836 (PMC3527610; doi:10.1371/journal.pone.0052836)
Supplement: File S1 — How to get started in BiologicalNetworks and step-by-step analysis for Use Case #1. (DOC) [file pone.0052836.s001.doc]

**Supplementary file S1.**

- [**Get started in BiologicalNetworks**](#get_started)**.**
- [**Use Case #1. OCT4 regulation in mammals ES cells. Study 1**](#case1_study1)**.**
- [**Use Case #1. OCT4 regulation in mammals ES cells. Study 2**](#case1_study2)**.**
- **Get started. Download and install BiologicalNetworks.
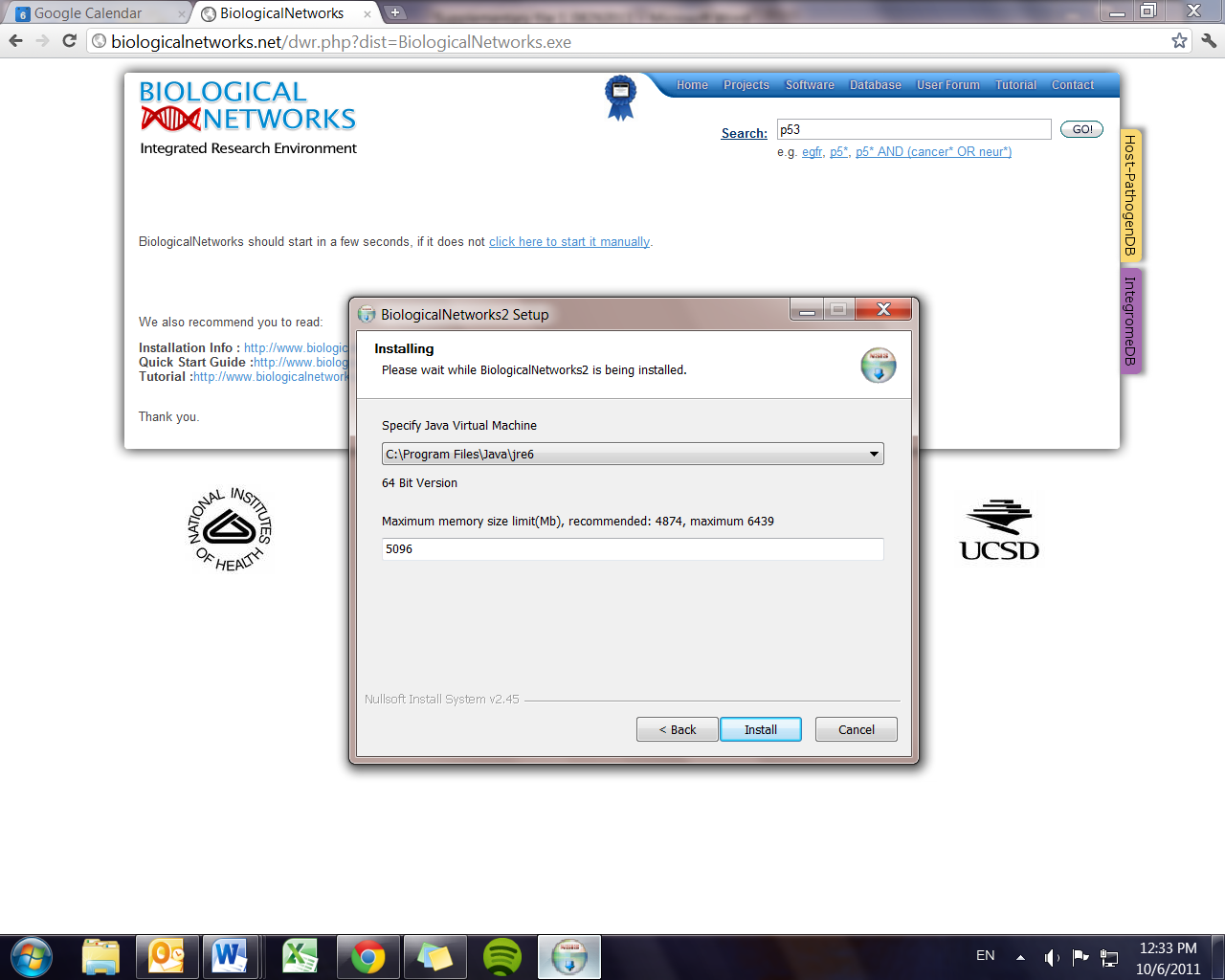
**

**
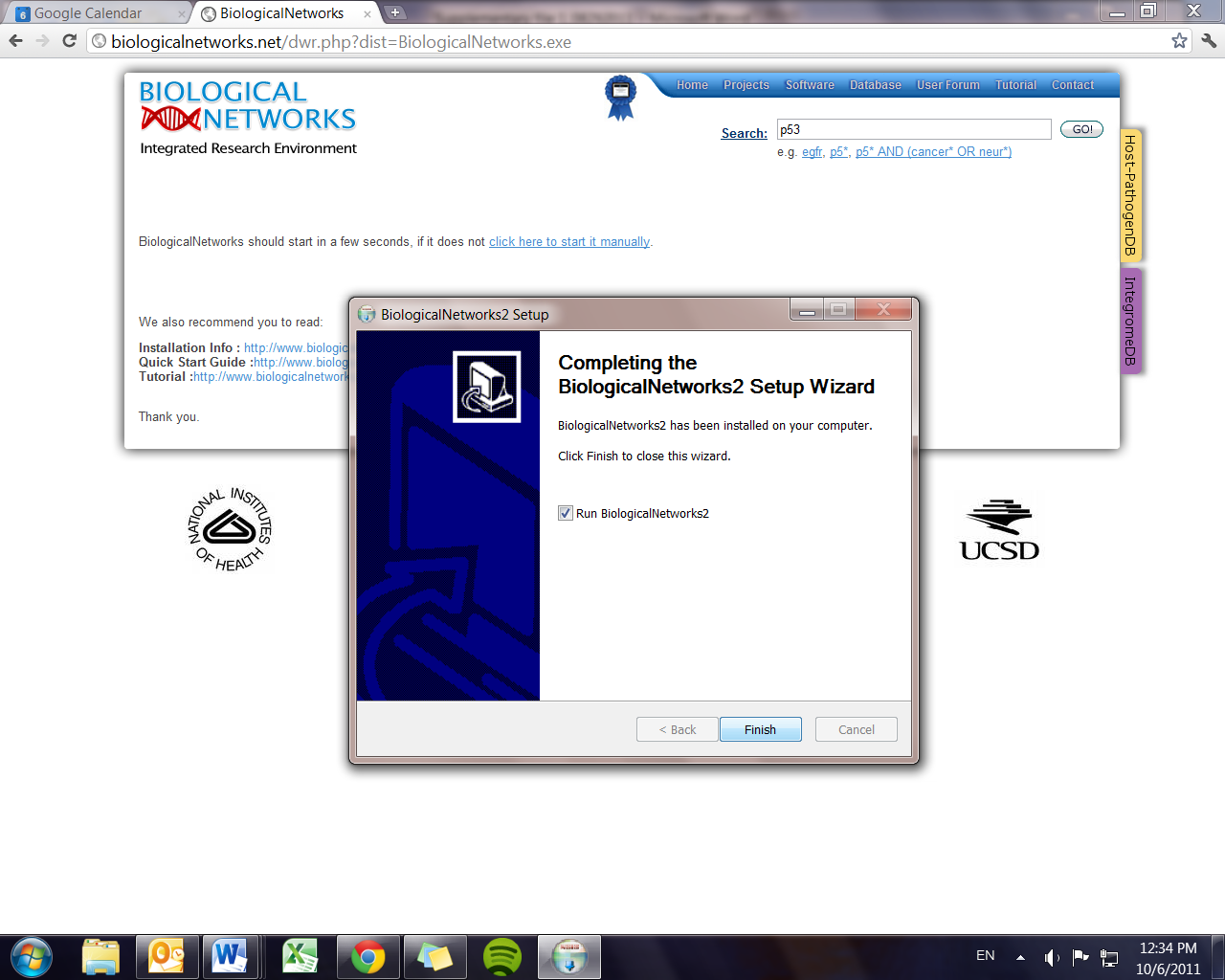
**

- **Create a new project.**

**
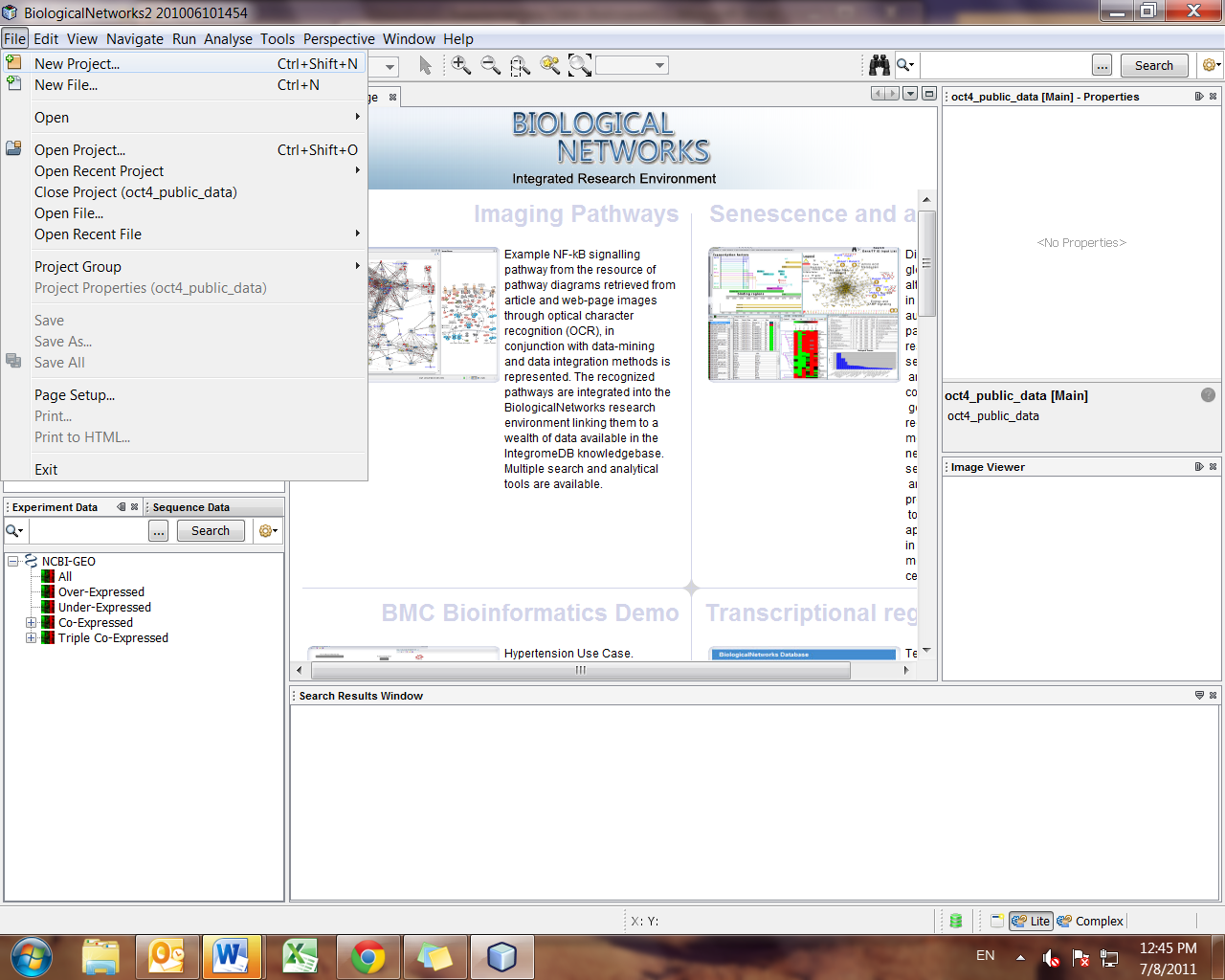
**

**
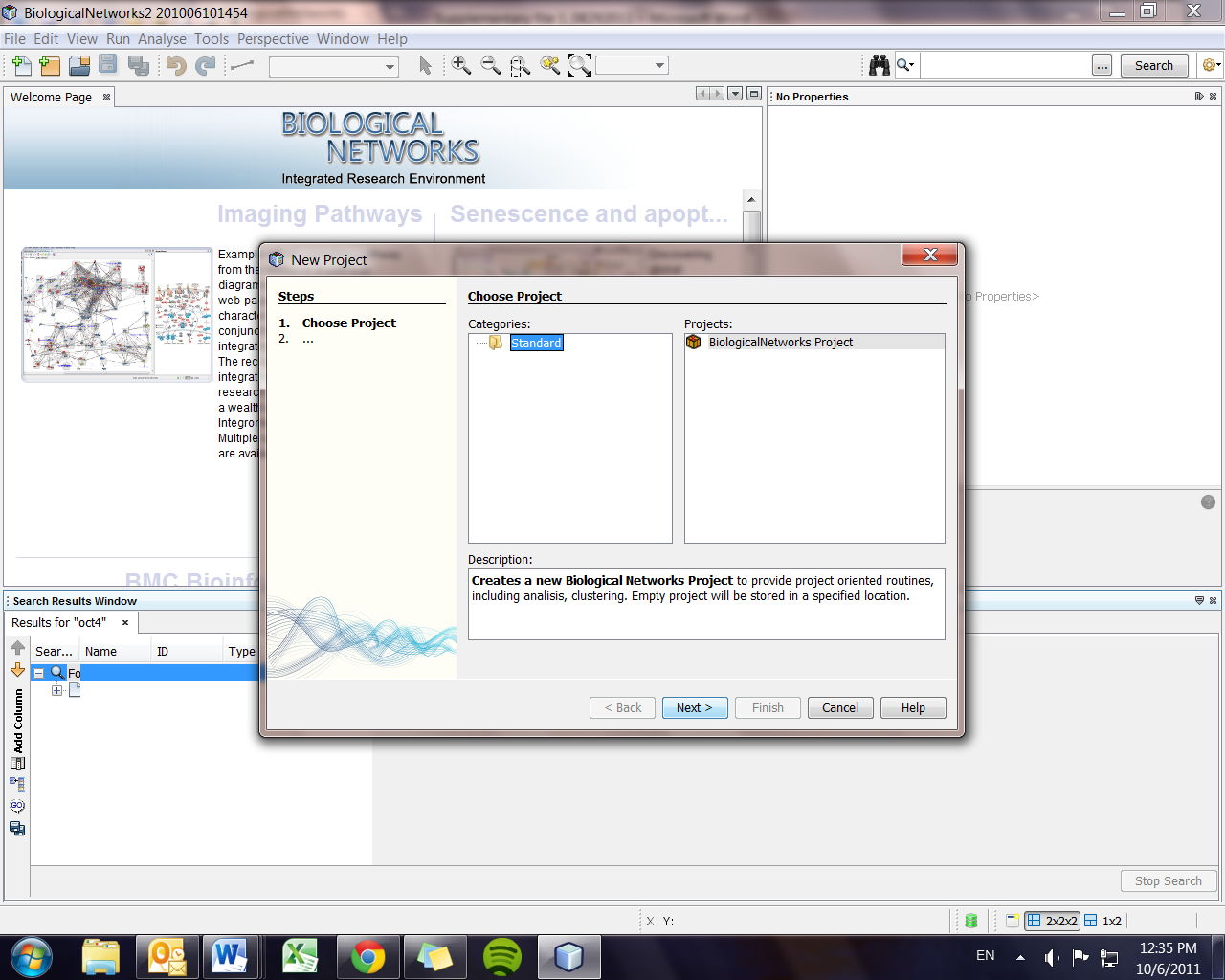
**

**
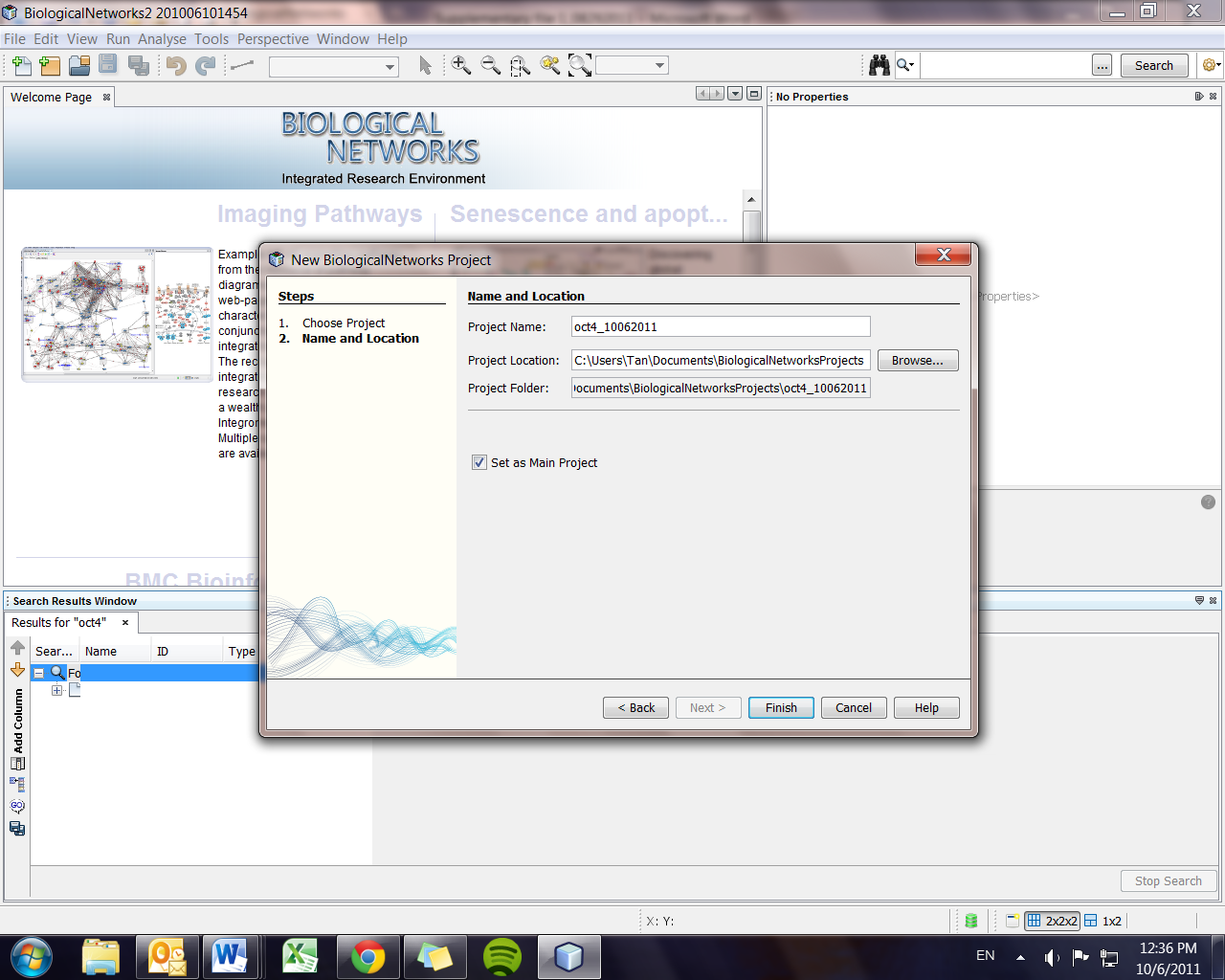
**

**Use Case #1. OCT4 regulation in mammals ES cells. Study 1.**

- **Search for OCT4 in mouse.** Type oct4 (case insensitive) in the search box (on the upper right corner of BiologicalNetworks), click on the left off the search box and select ‘Mus musculus’, and click ‘Search’.
- **
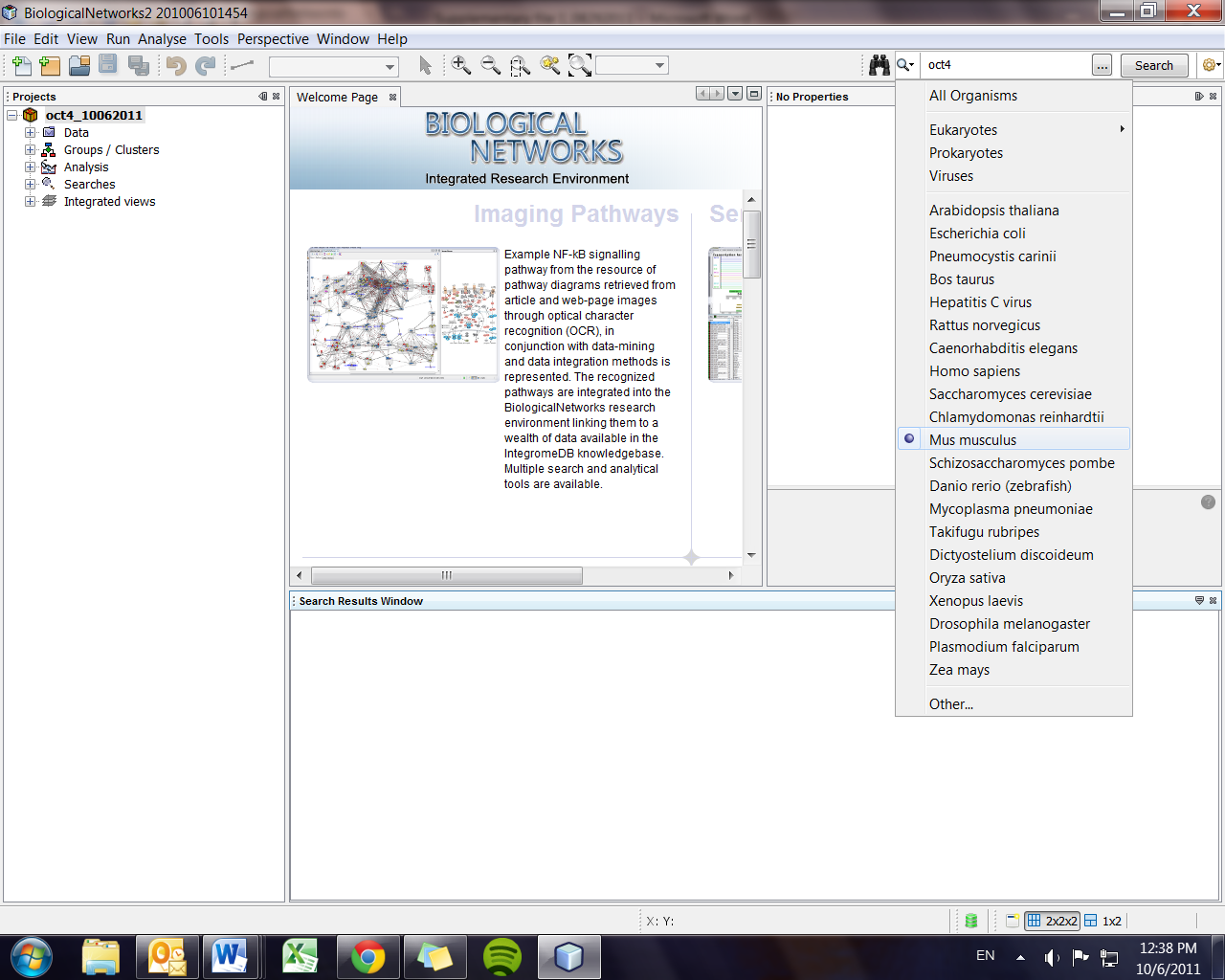
**
- In the search result window, select OCT4 protein (under the name ENSMUSP00000025271) and, using the mouse’s right button, select ‘Built Transcription Regulatory Network Wizard.’ **
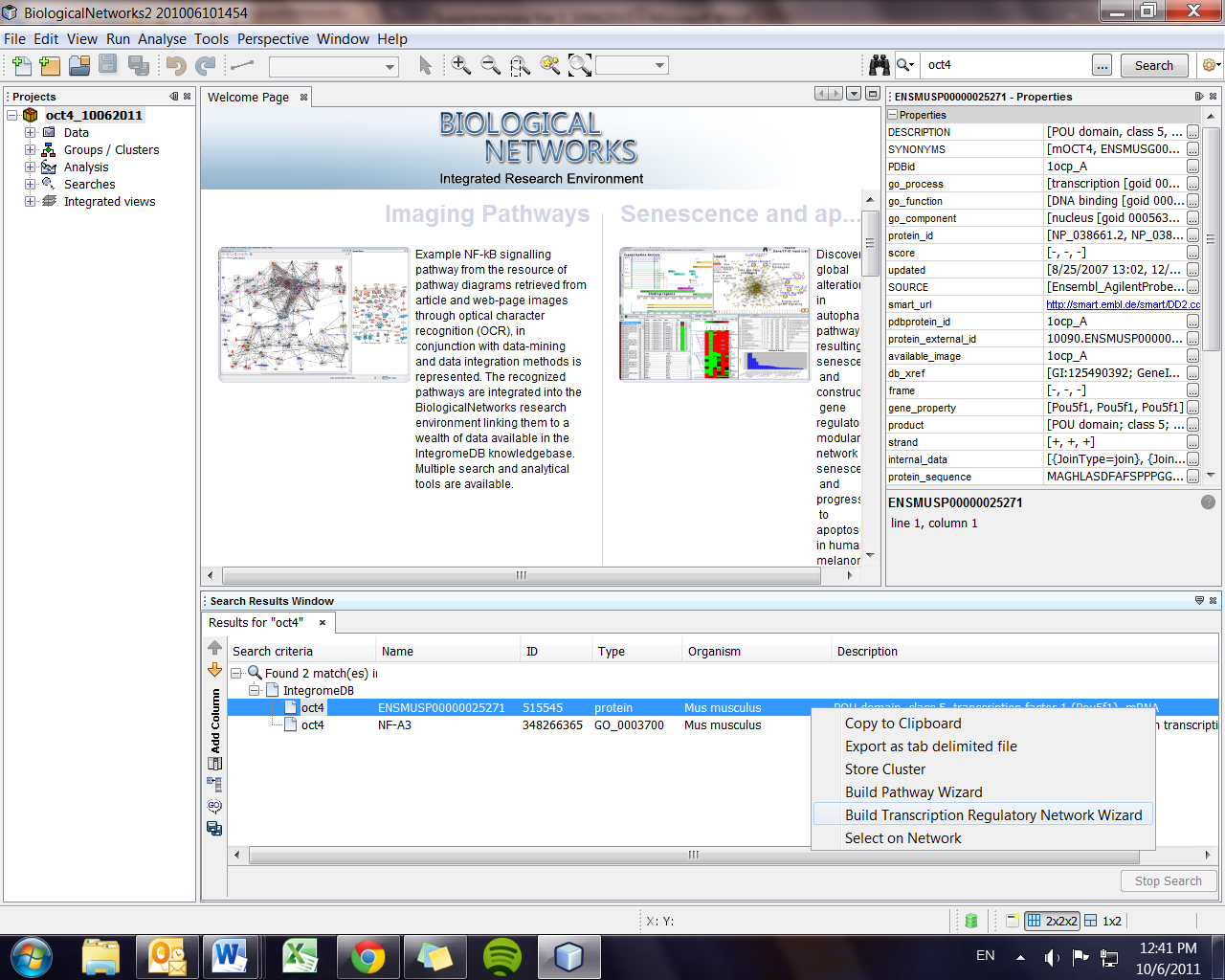
**
- **Specify genes/TFs and parameters for homology search.** In the wizard, select ’Select TFs’ for OCT4 and unselect ‘Select Genes’, select ‘Skip homologous search’, and click ‘Next’. **
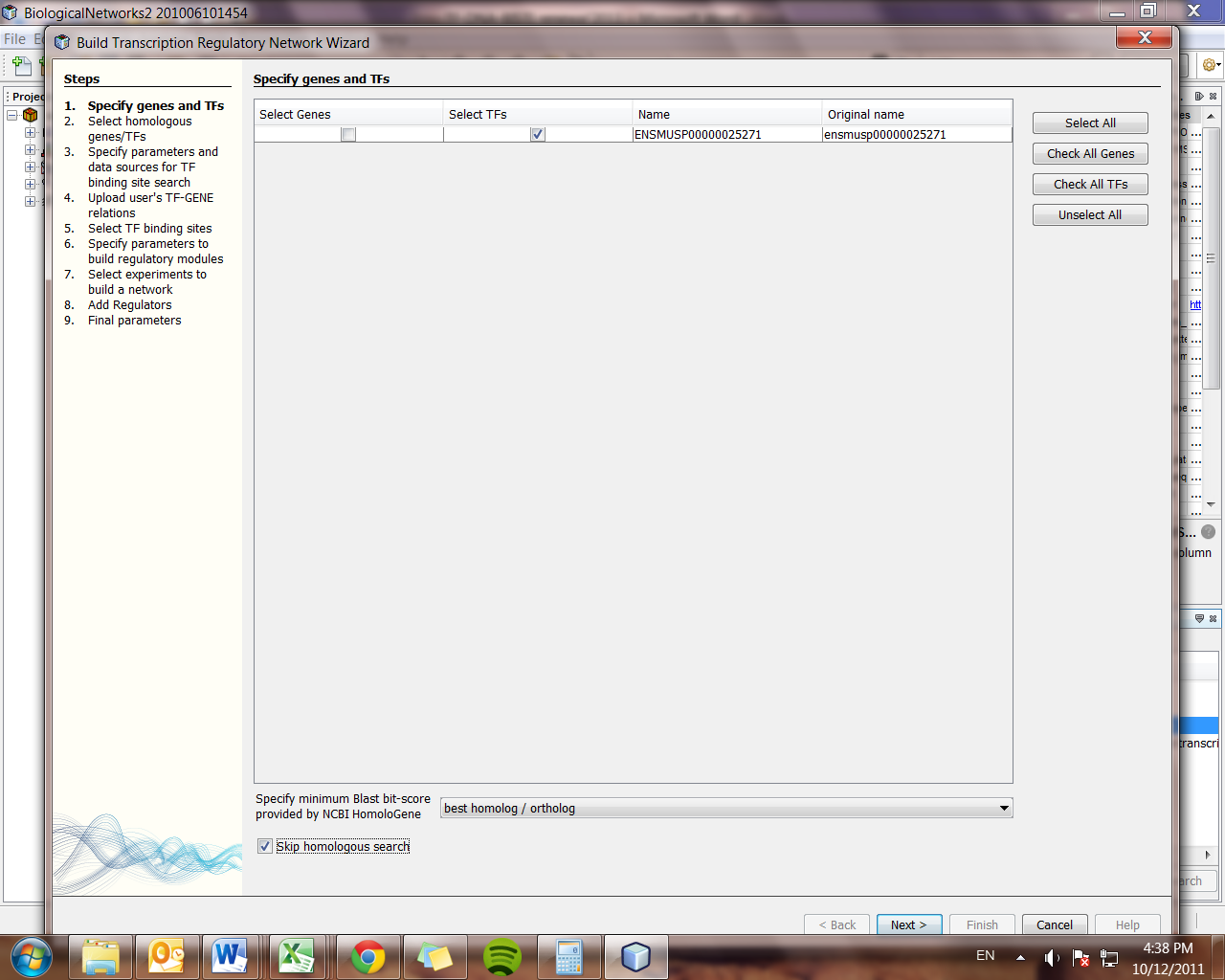
**
- **Specify parameters for TF binding sites search.** Unselect all data sources and select the check box ‘Upload own TF-gene relationships’. Click ‘Next.’

**
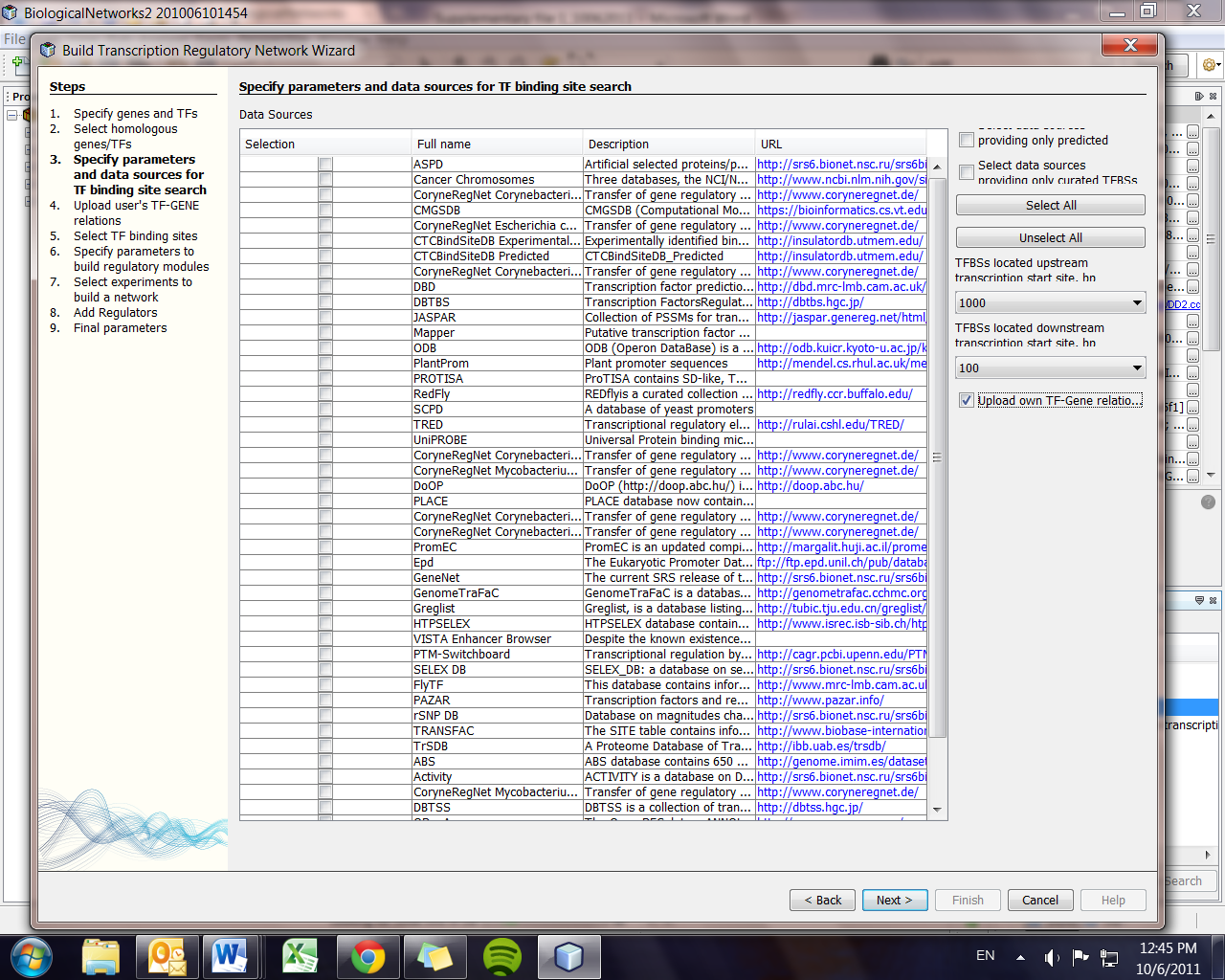
**

- **Upload Supplementary file S5.**

**
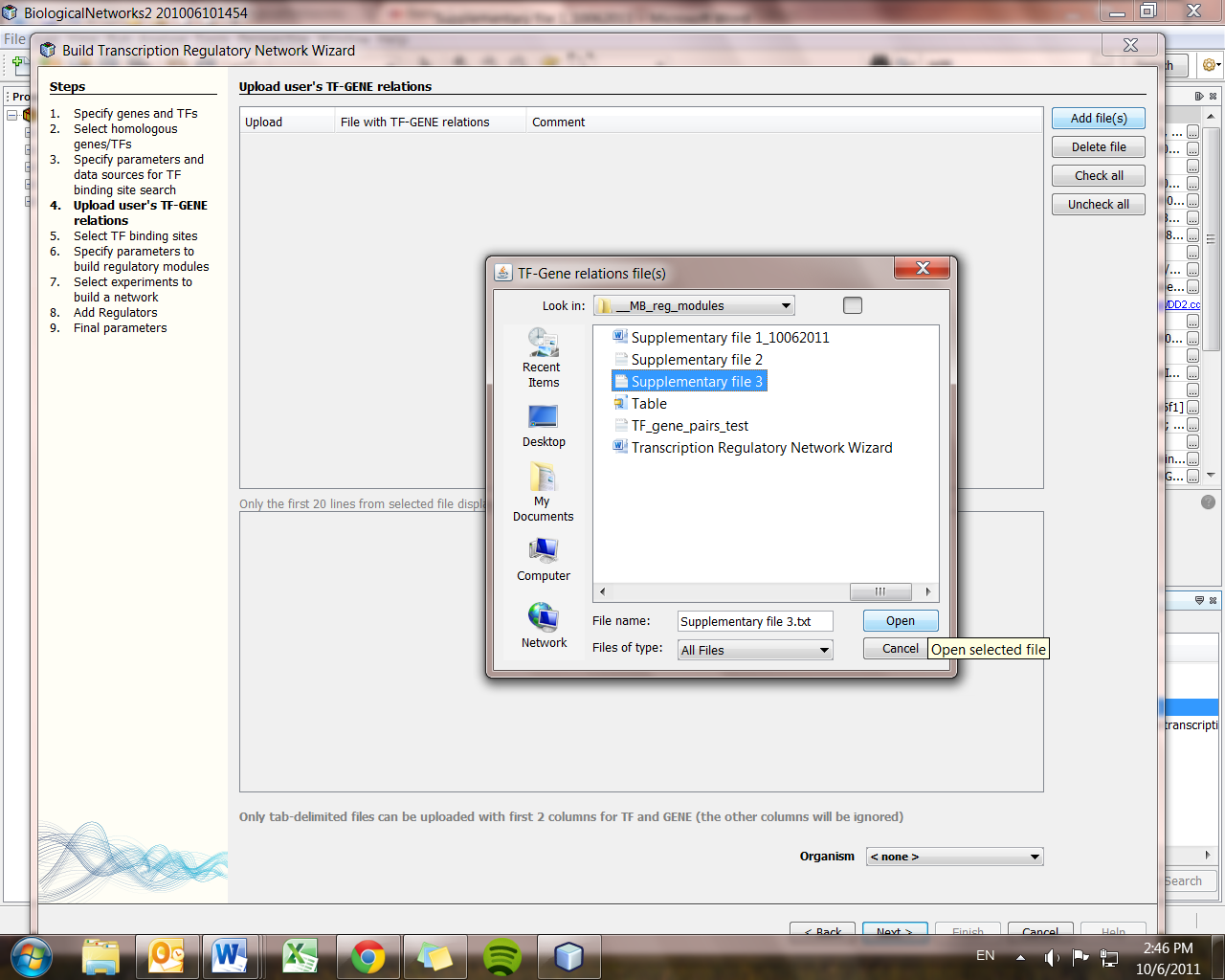
**

- **Select the main organism, *Mus musculus*.** Otherwise, the system will look for homologous genes in all species. Only the top few pairs are seen in the window; it is done for checking the proper formatting only.

**
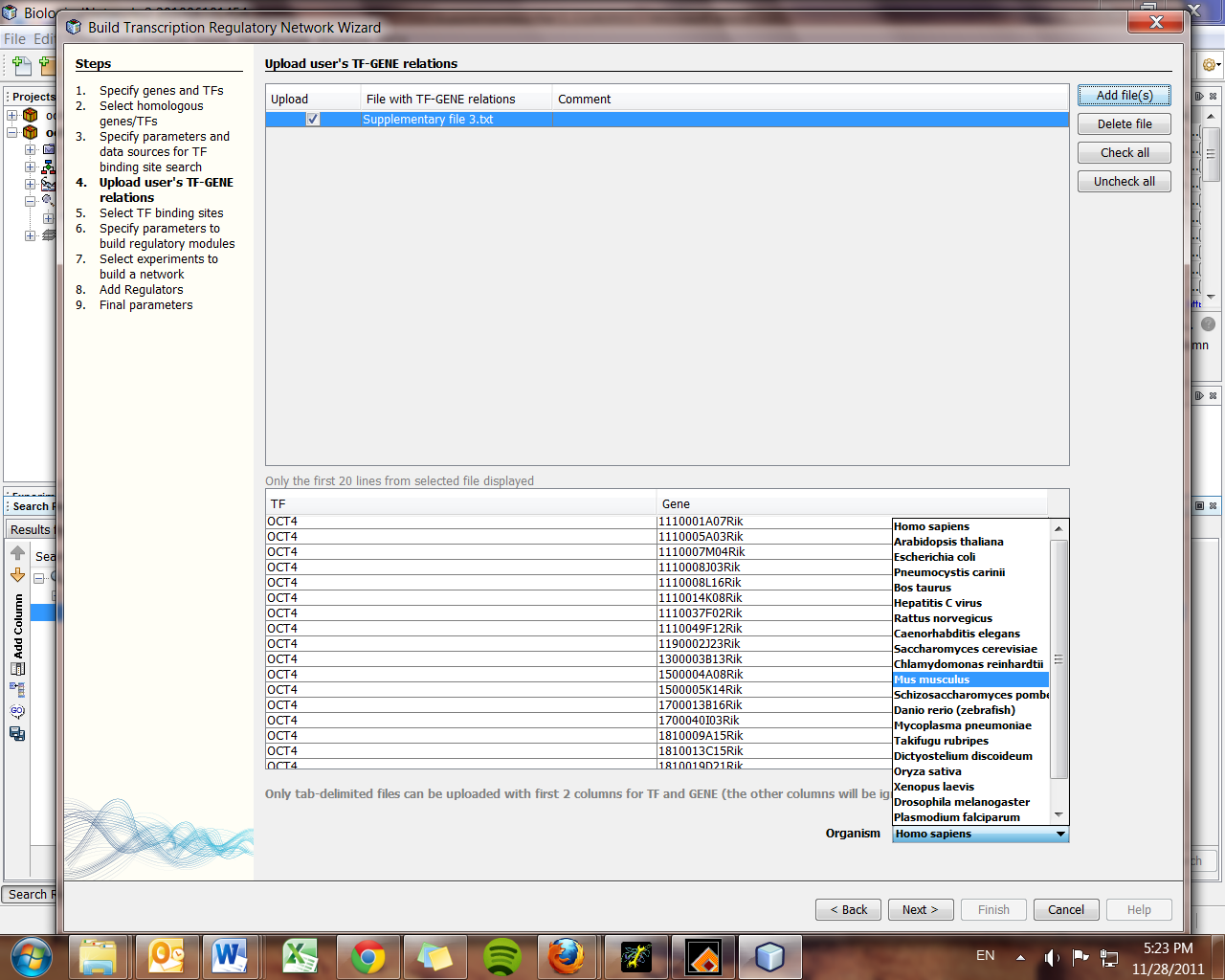
**

- **Select TF binding sites.** By default all found sites are selected. Use this default. Make sure that Mus musculus is specified as a main organism (that is an organism for which genes the network will be built and to which genes human (or other species if selected) genes and TFs will be mapped. Click ‘Next.’

**
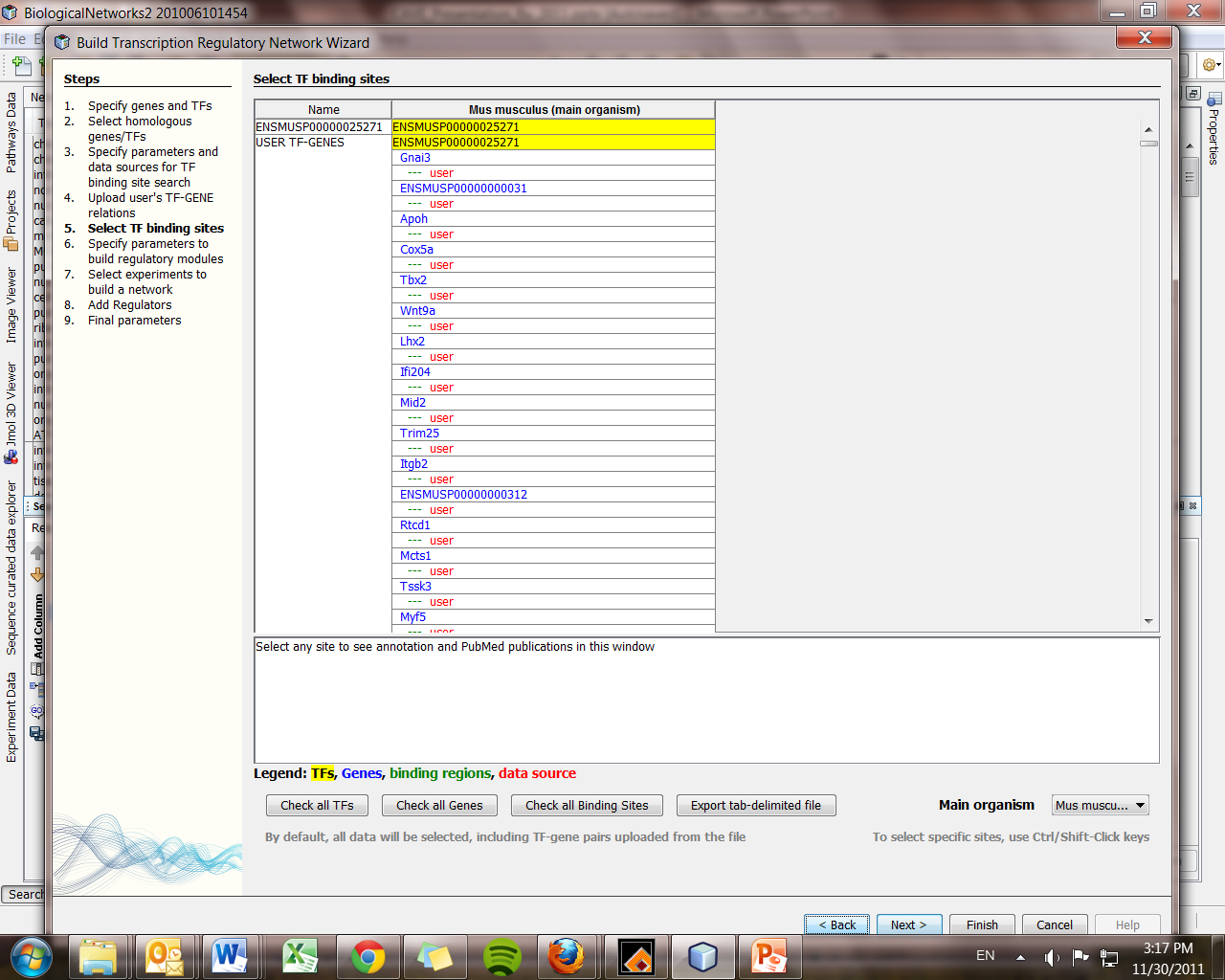
**

- **Specify parameters to build regulatory modules.** Specify p-value at 0.0001 (1.0E-4), select *‘User’s data’* for the source of microarray experiments, and upload **Supplementary file S4**. Click ‘Next.’

**
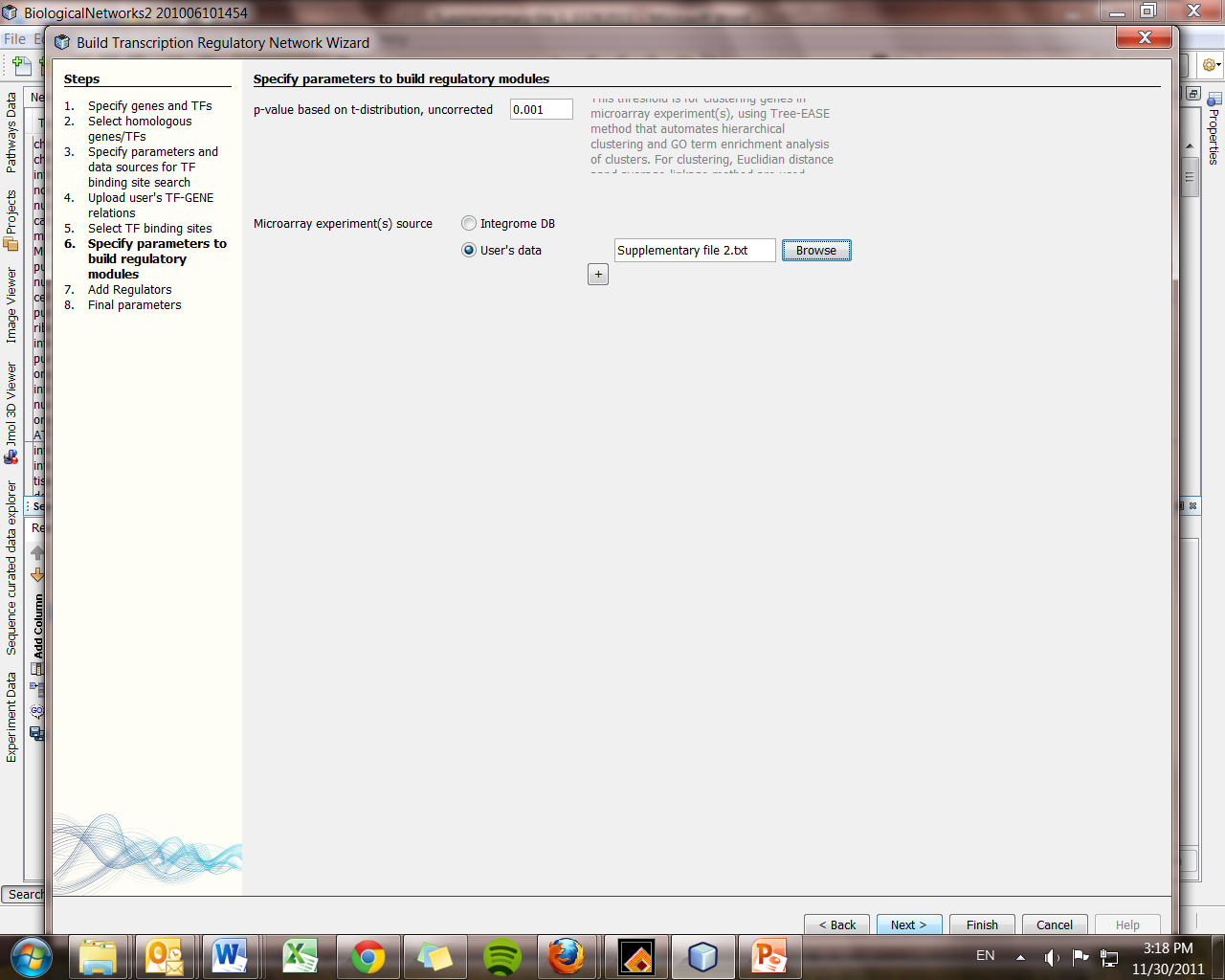
**

- **Select/unselect regulators.** Unselect the box ‘Add Regulators’. Click ‘Next’.
- **
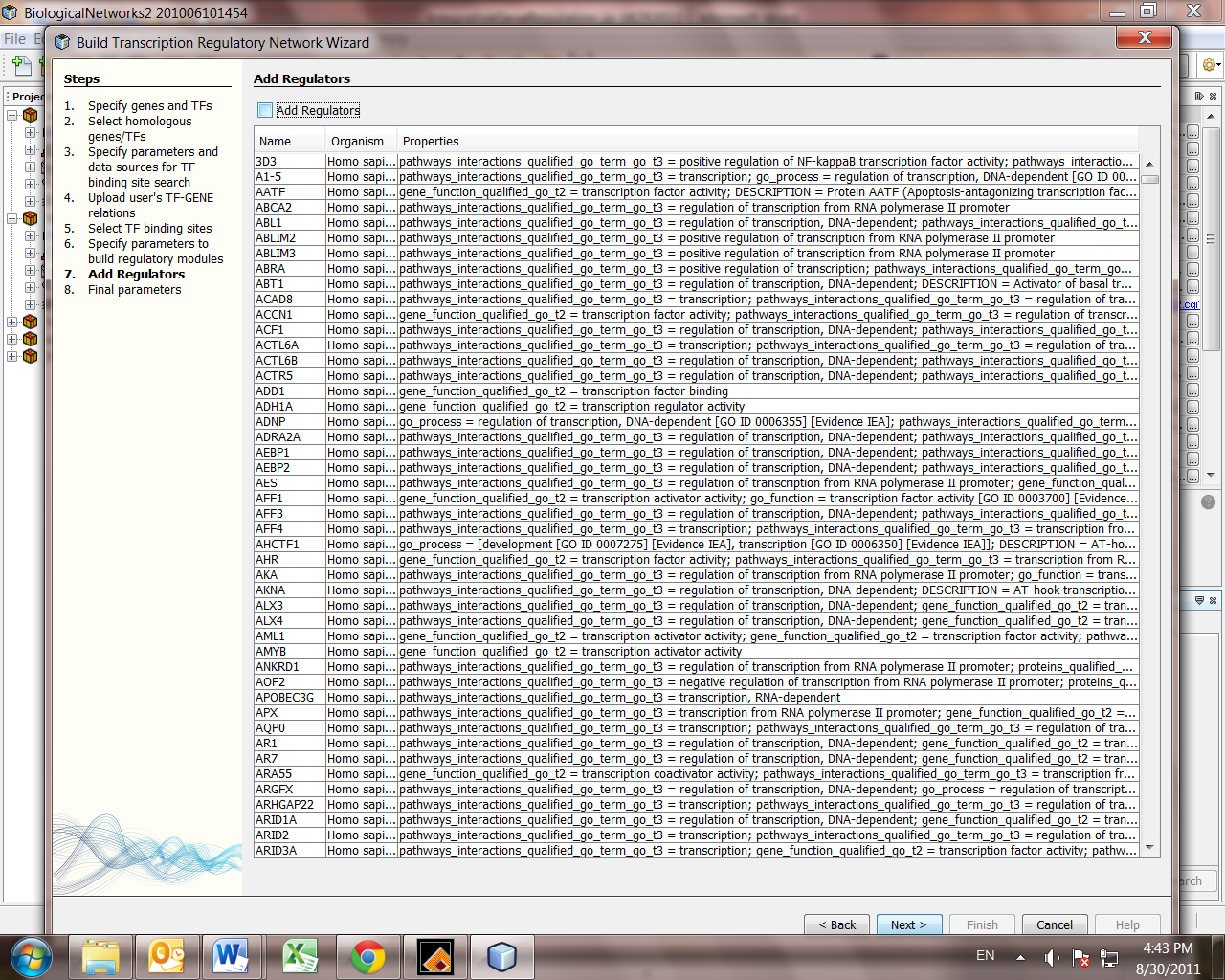
**
- **Final parameters.** Select/unselect ‘Create Gene Regulatory Network’. If this check box is unselected, only regulatory modules will be infered. Building and visualization of the network takes additional time, and for a large network it can be very time-consuming. For this study, unselect this check box. Also, select Pearson correlation as a distance for clustering and click ‘Finish’. At this point, the Wizard finishes its work, and the modules will start to be infered. Make sure to check data that will be submitted for calculation; for example, Oct, March, or Sept genes might be converted to dates if the file was prepared in Excel. If this is the case, close the Wizard and launch it *de novo*. Move the lever if the window for the input set appears empty.

**
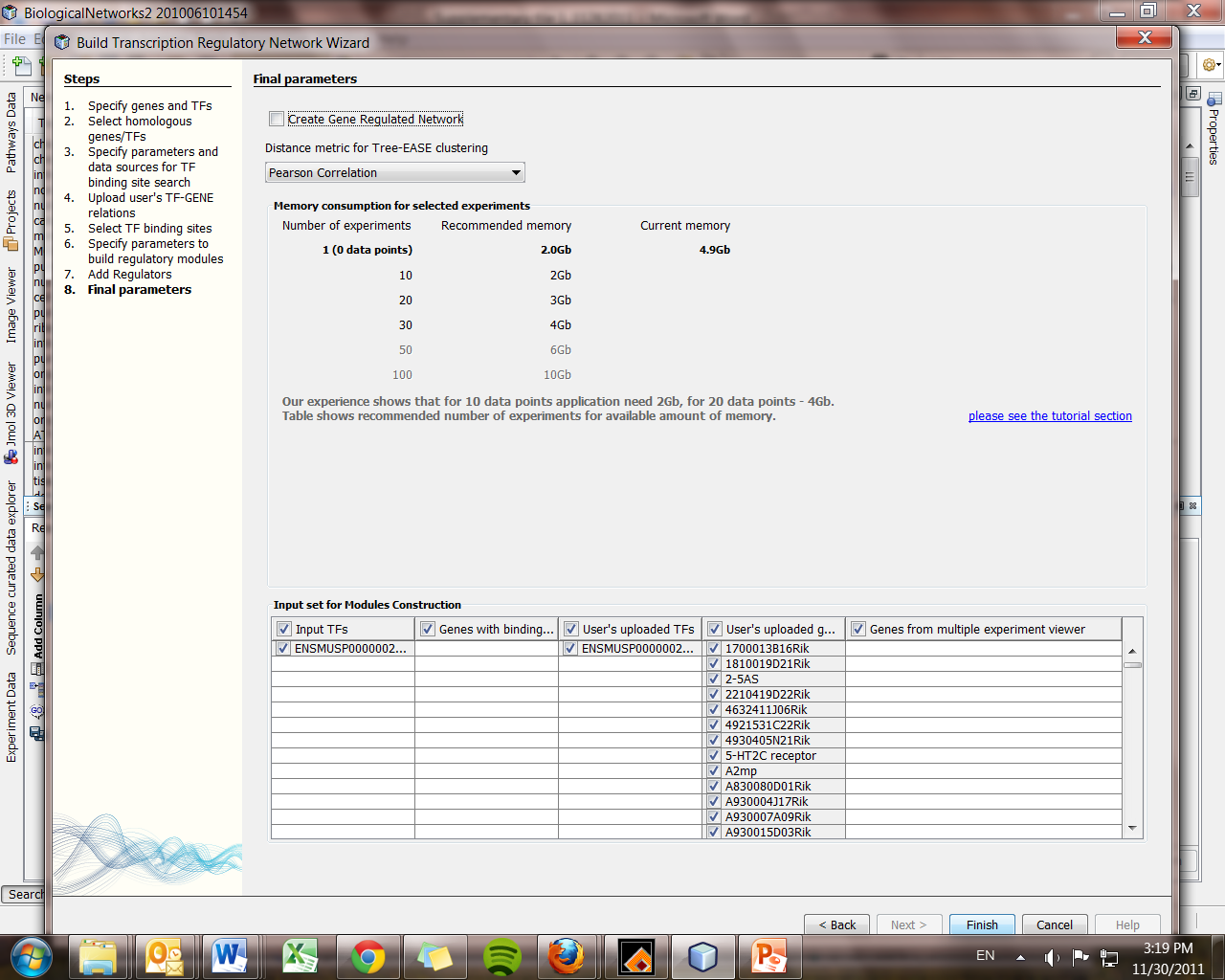
**

- **Loading the user’s microarray data.** In several minutes, the program will ask to specify the location of the left uppermost cell providing values for microarray data and specify the spieces. After specifying this, click ‘Load’. **
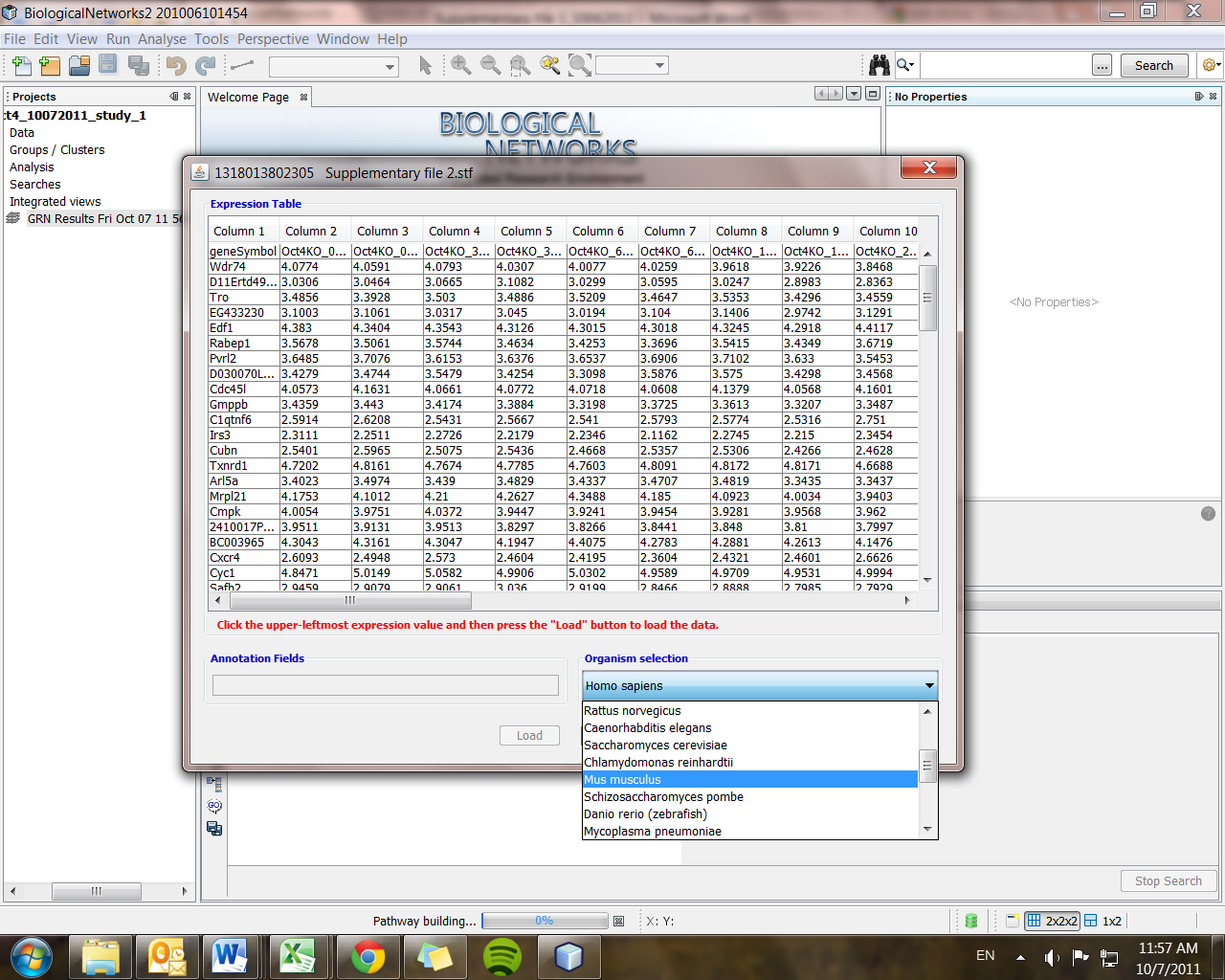
**
- **The progress of BiologicalNetworks work can be seen by clicking on the bottom running bar.
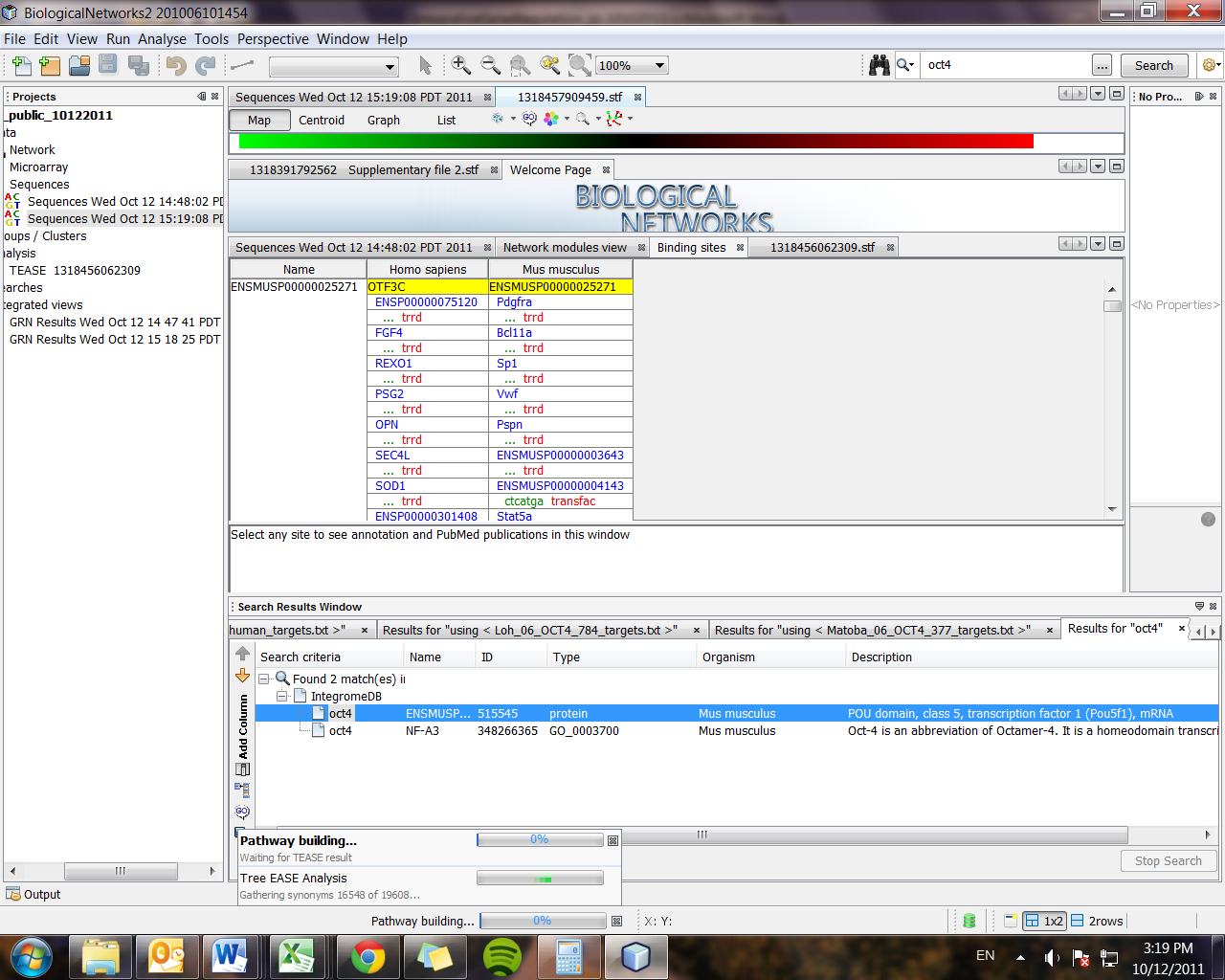
**
- **Clustering of microarray data.** Move mouse to the colored circle to see significant GO terms for the genes in the cluster.

**
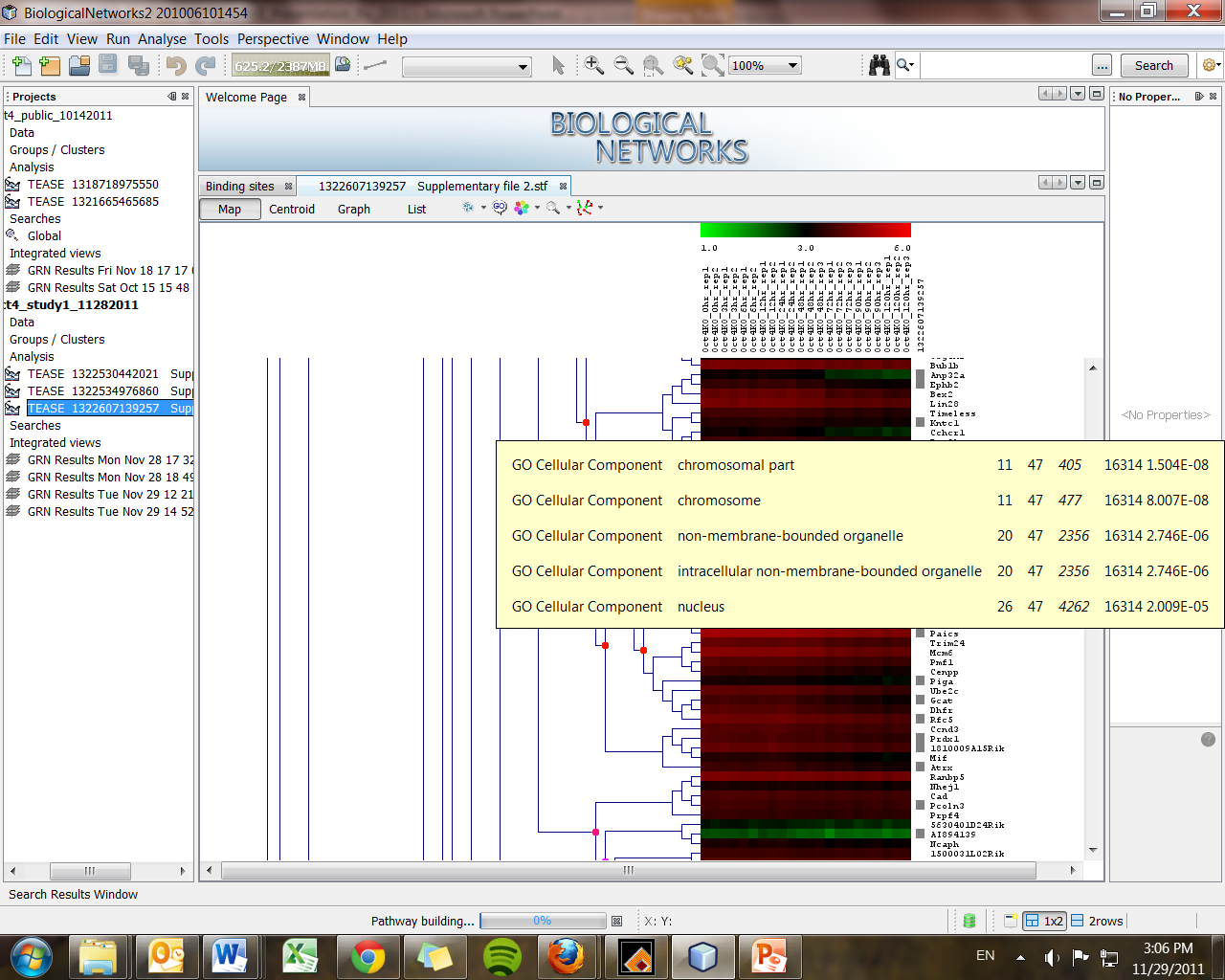
**

- **Results for Study 1: inferred modules.** The modules are initially sorted by the number of first ‘red genes’, then ‘blue genes’ (see Figure 3 and the text for detail).
- **
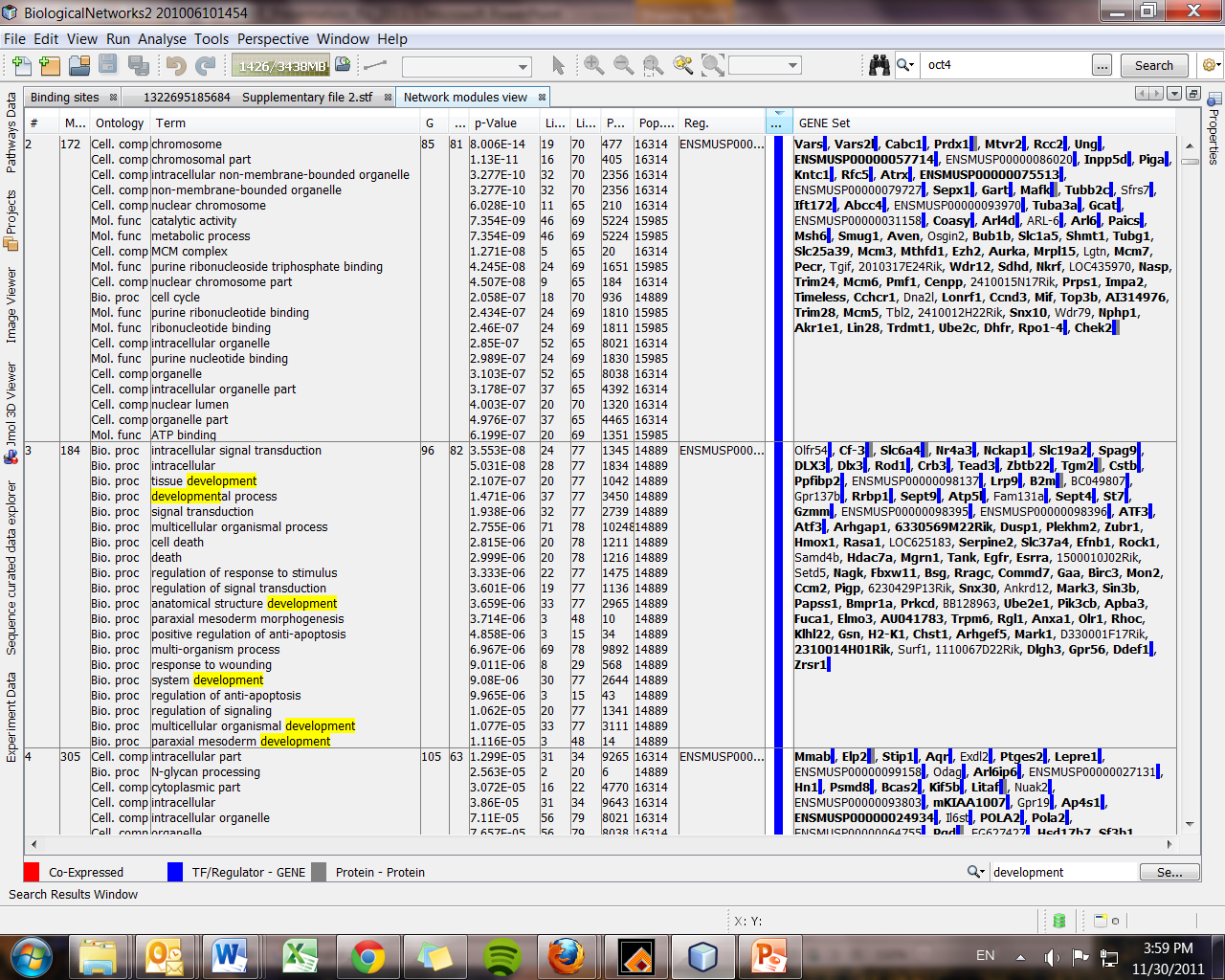
**
- **The modules can be sorted, for examle, by the number of GO terms (click on the ‘List Hits’ column).**
- **
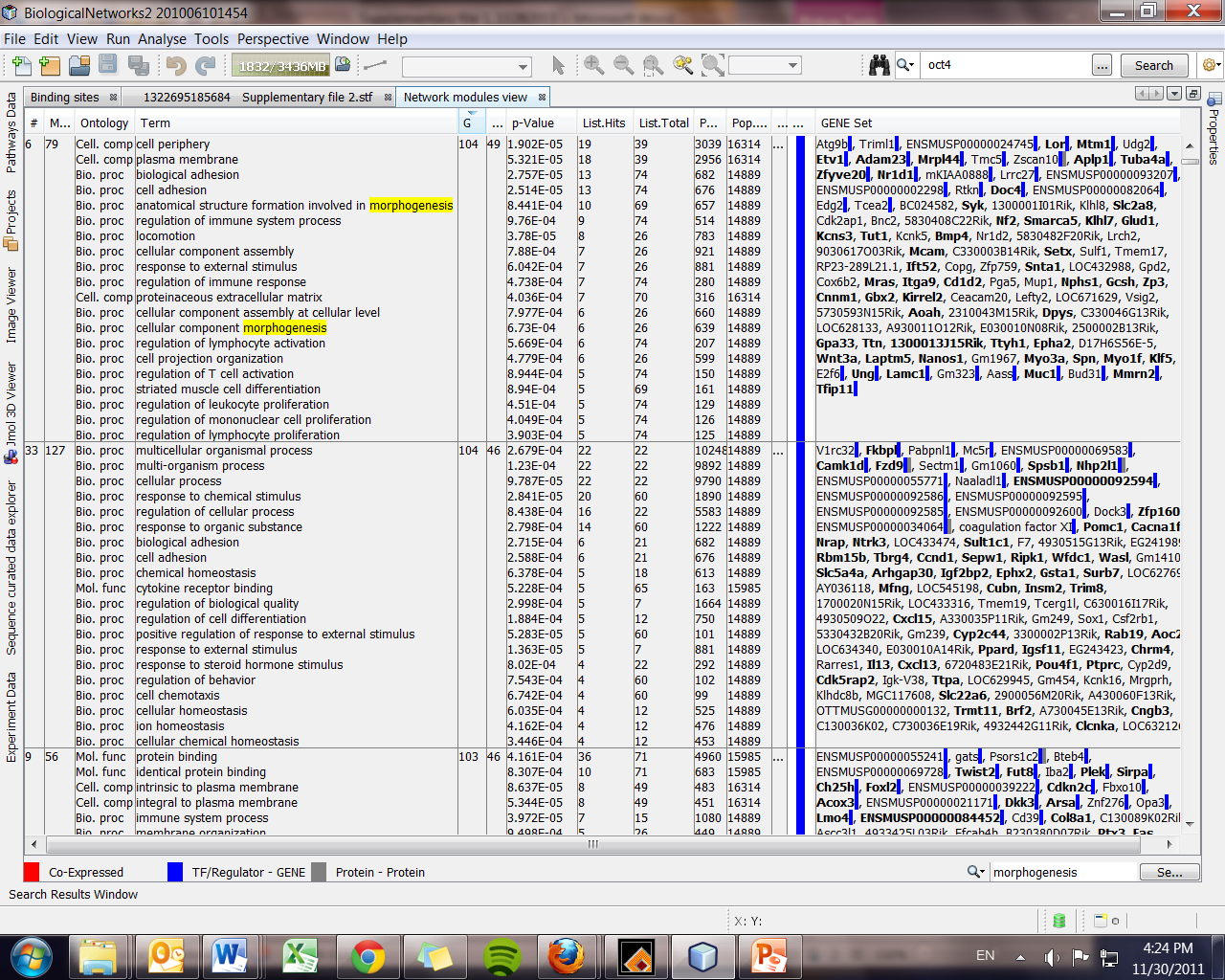
**
- **Save modules in the file.** The modules can be selected and, using the right mouse button, exported in the text file. For this Study 1, the modules have been saved in **Supplementary file S6**. **
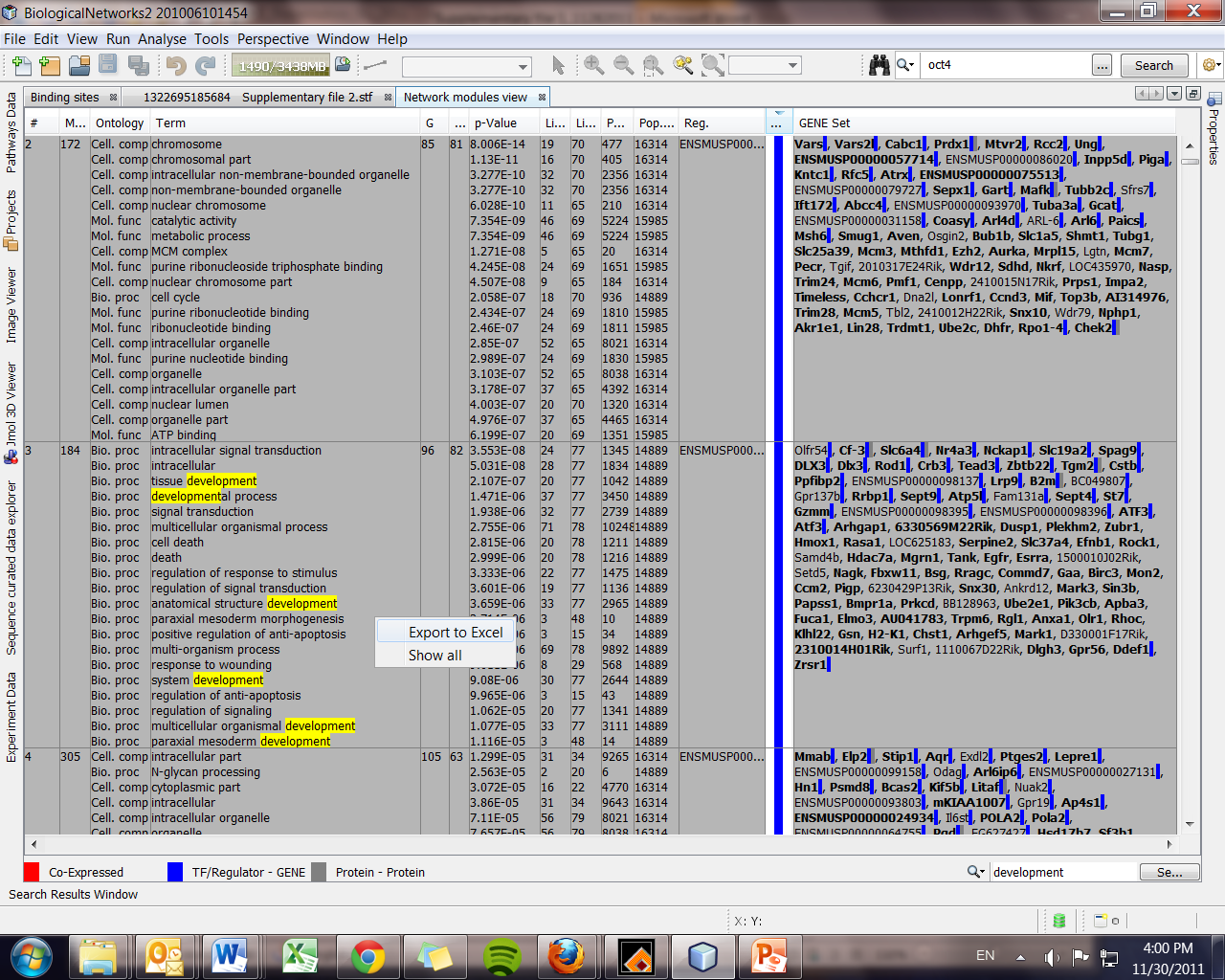
**

**Use Case #1. OCT4 regulation in mammals ES cells. Study 2.**

- **Search for OCT4 (see screen shots of Study 1 above).** Type oct4 (case insensitive) in the search box (on the upper right corner of BiologicalNetworks), click on the left off the search box and select ‘Mus musculus’, and click ‘Search’.
- In the search result window, select proteins and using the mouse’s right button select ‘Built Transcription Regulatory Network Wizard.’ **(see screen shots of Study 1 above).**
- **Specify genes/TFs and parameters for homology search.** In the wizard, select ’Select TFs’ for OCT4 and unselect ‘Select Genes’, select best homologs and click ‘Next’.
- **
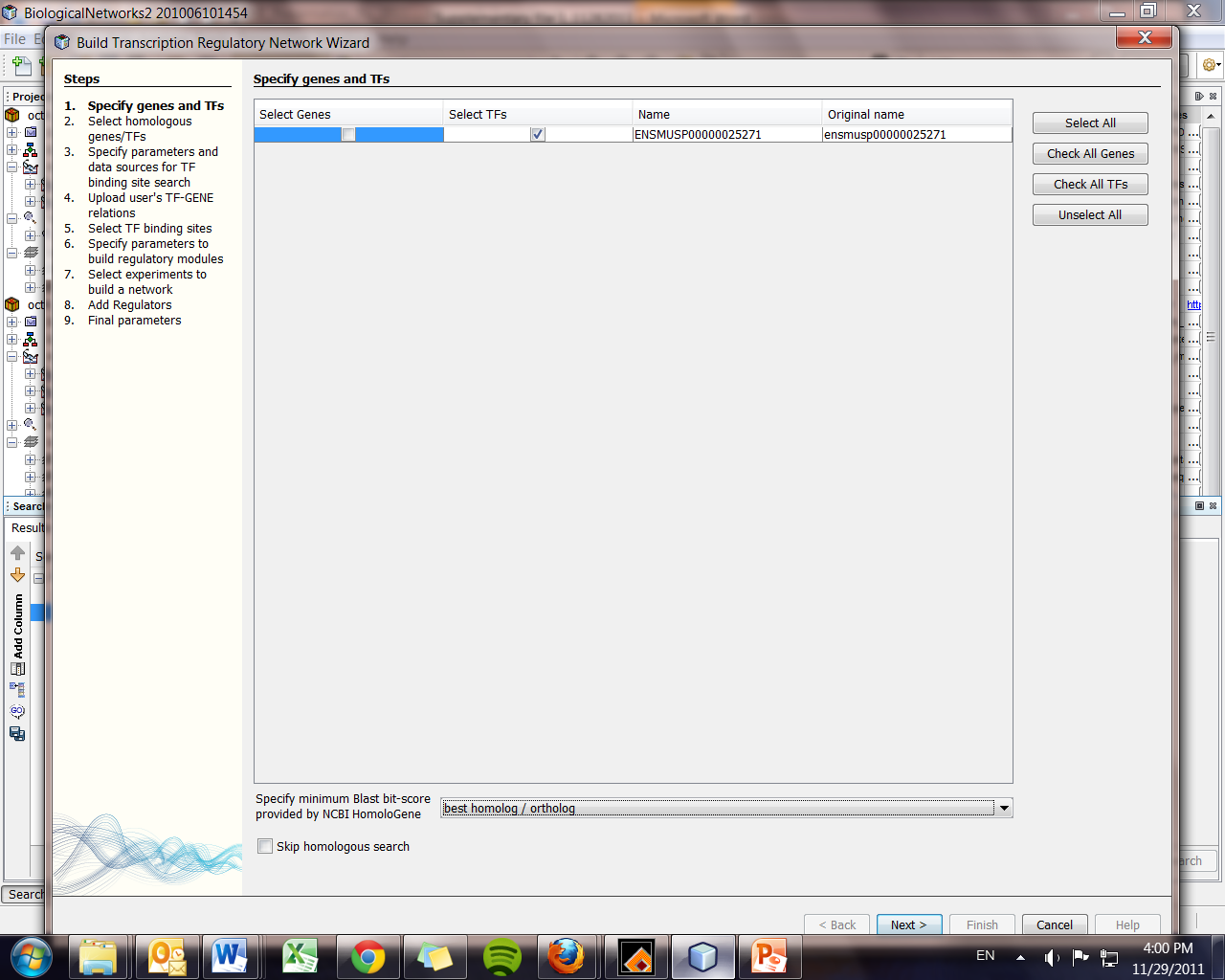
**
- **Select homologous genes/TFs.** To select homologous TFs, expand the window, clicking on the ‘Expand Cells’ button (on the bottom). Select all TFs for mouse and human (use Ctrl+Click for selection) *(rat TFs can be also selected; however, OCT4 binding sites in rat were not found)*. Click ‘Next.’

**
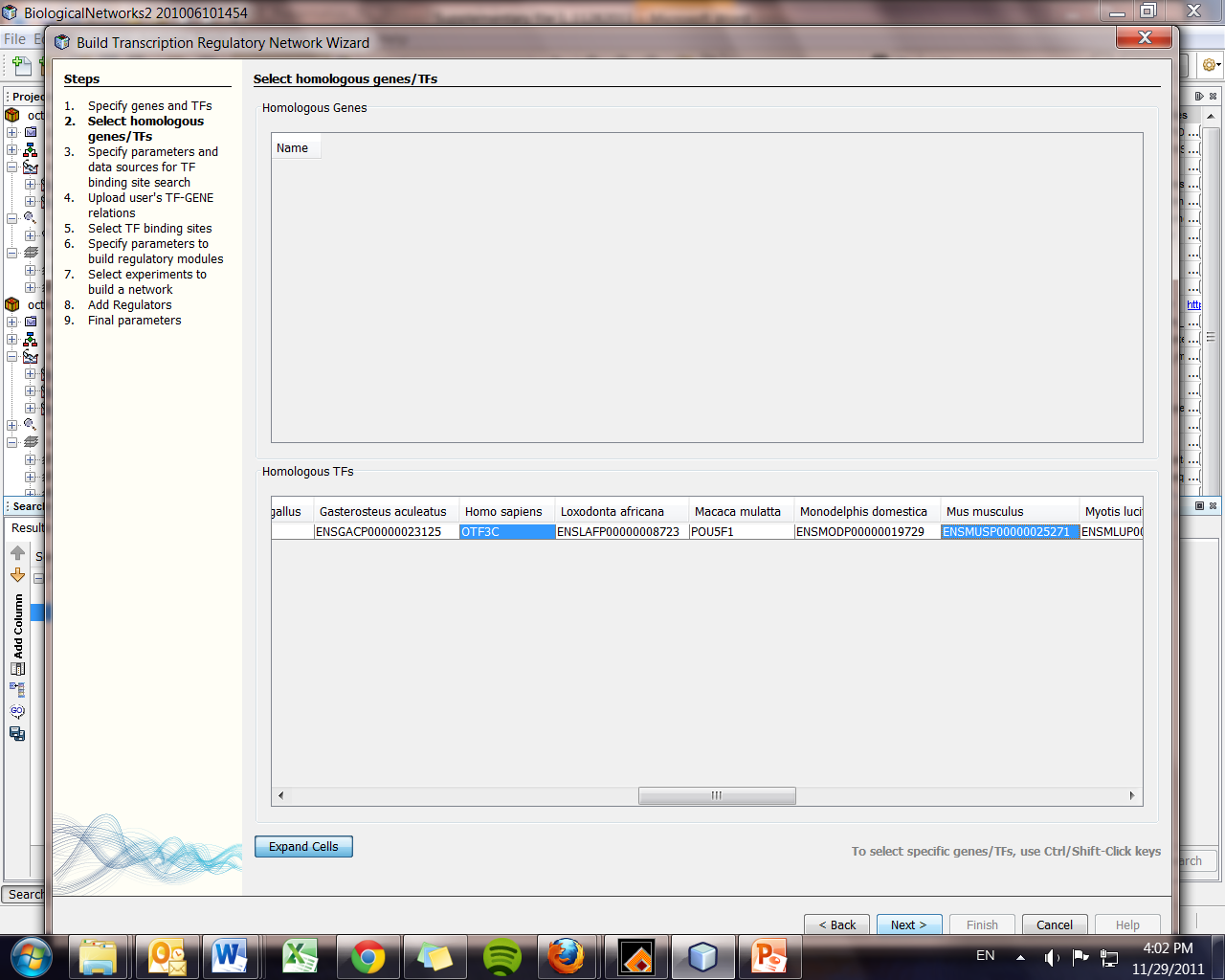
**

- **Specify parameters for TF binding sites search.** Select all data sources (default). Select 10,000 bp for upstream and 1000 bp for downstream regulatory region. Click ‘Next.’ **
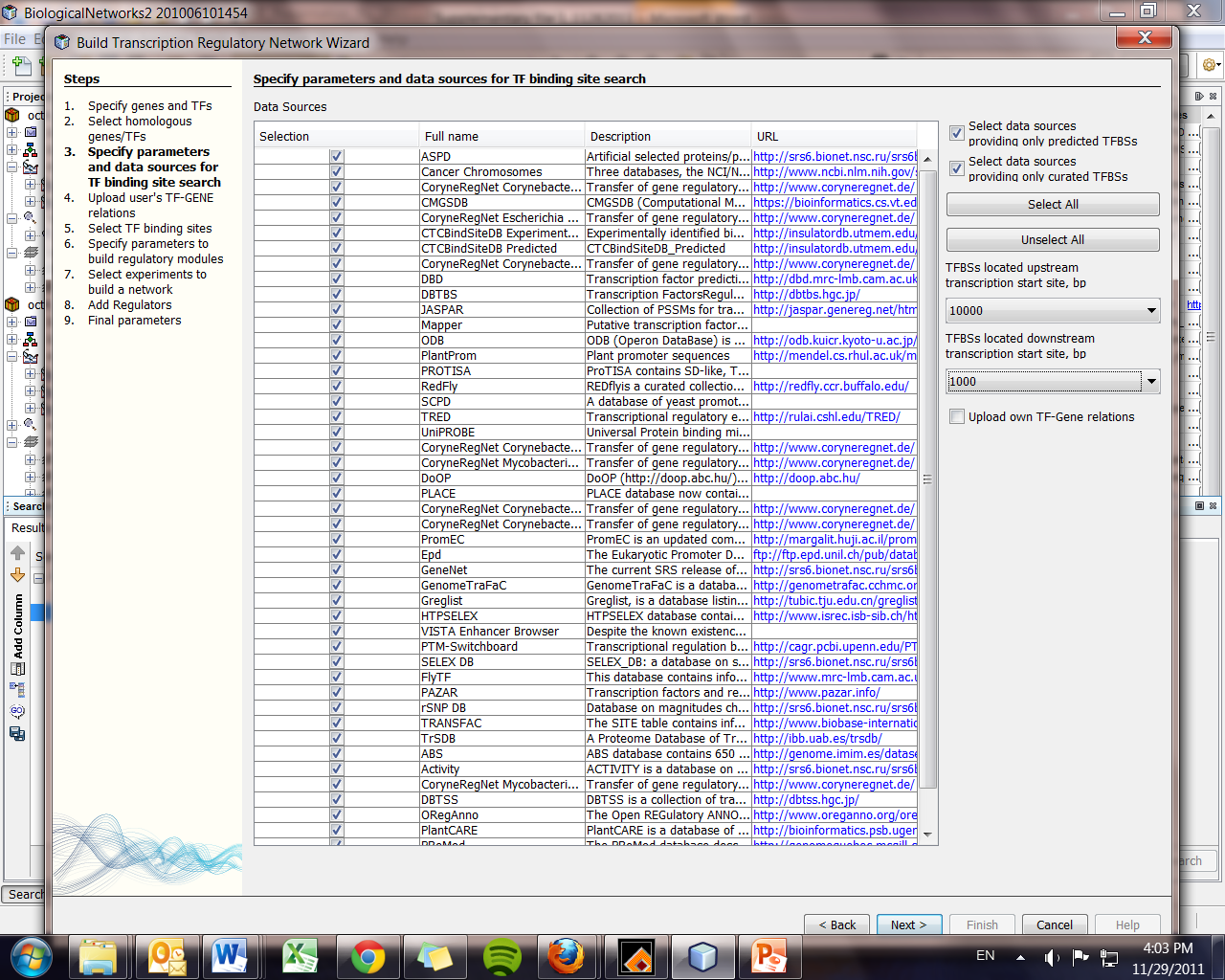
**

- **Select TF binding sites.** By default all TFs and target genes are selected. Select ‘Check all TFs’ to search for microarrays in which the genes co-expressed with OCT4 only and not with its targets. Make sure that Mus musculus is specified as a main organism (that is an organism for which genes the network will be built and to which genes human genes and TFs will be mapped; mouse is selected because much more microarray and PPI data are available for mouse than human). Click ‘Next.’
- **
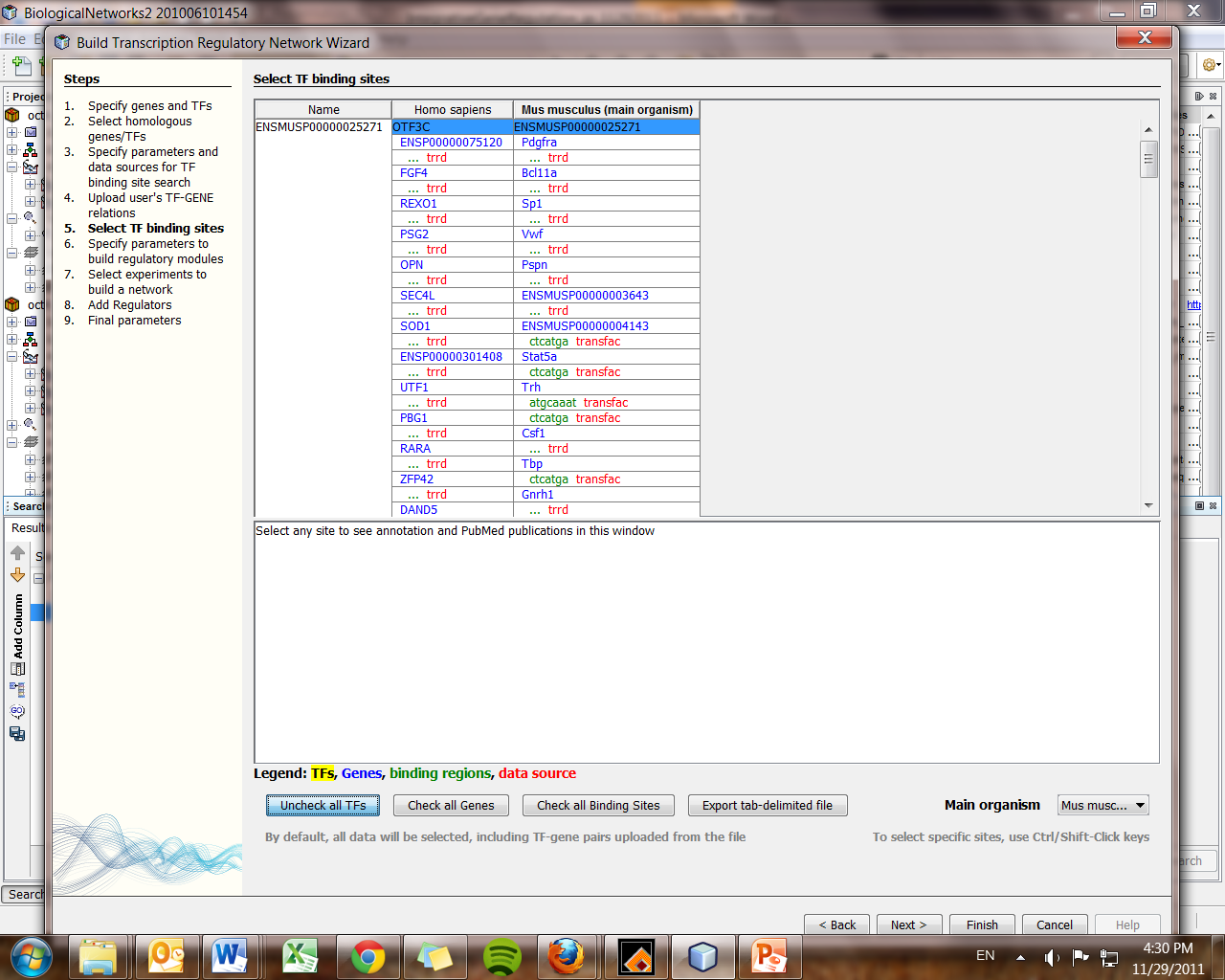
**

- **Specify parameters to build regulatory modules.** Specify p-value of 0.001 (1.0E-3) and IntegromeDB as a source, to consider all microarray experiment integrated from GEO and ArrayExpress in which selected genes/TFs, their targets and regulated TFs, and regulators are co-expressed in pairs). Click ‘Next.’
- **
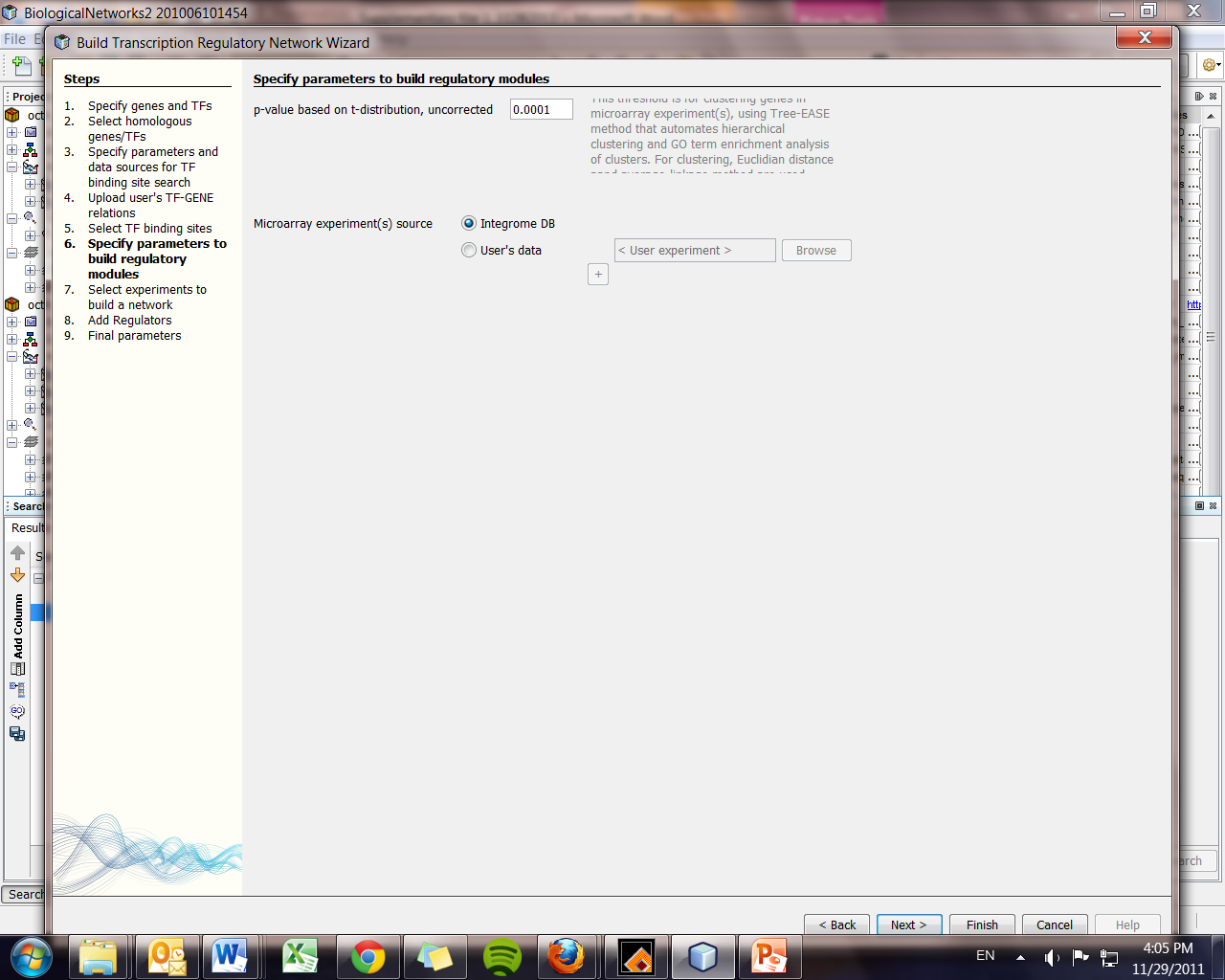
**
- **Select microarray experiments.** Check the button ‘Check by keyword’, select the keyword ‘Embryonic stem cell’ (more words can be selected using the Ctr-right mouse button; all selected keywords can be unselected using the button ‘Reset Selected terms’). Click ‘Recalculate using selected terms’. **
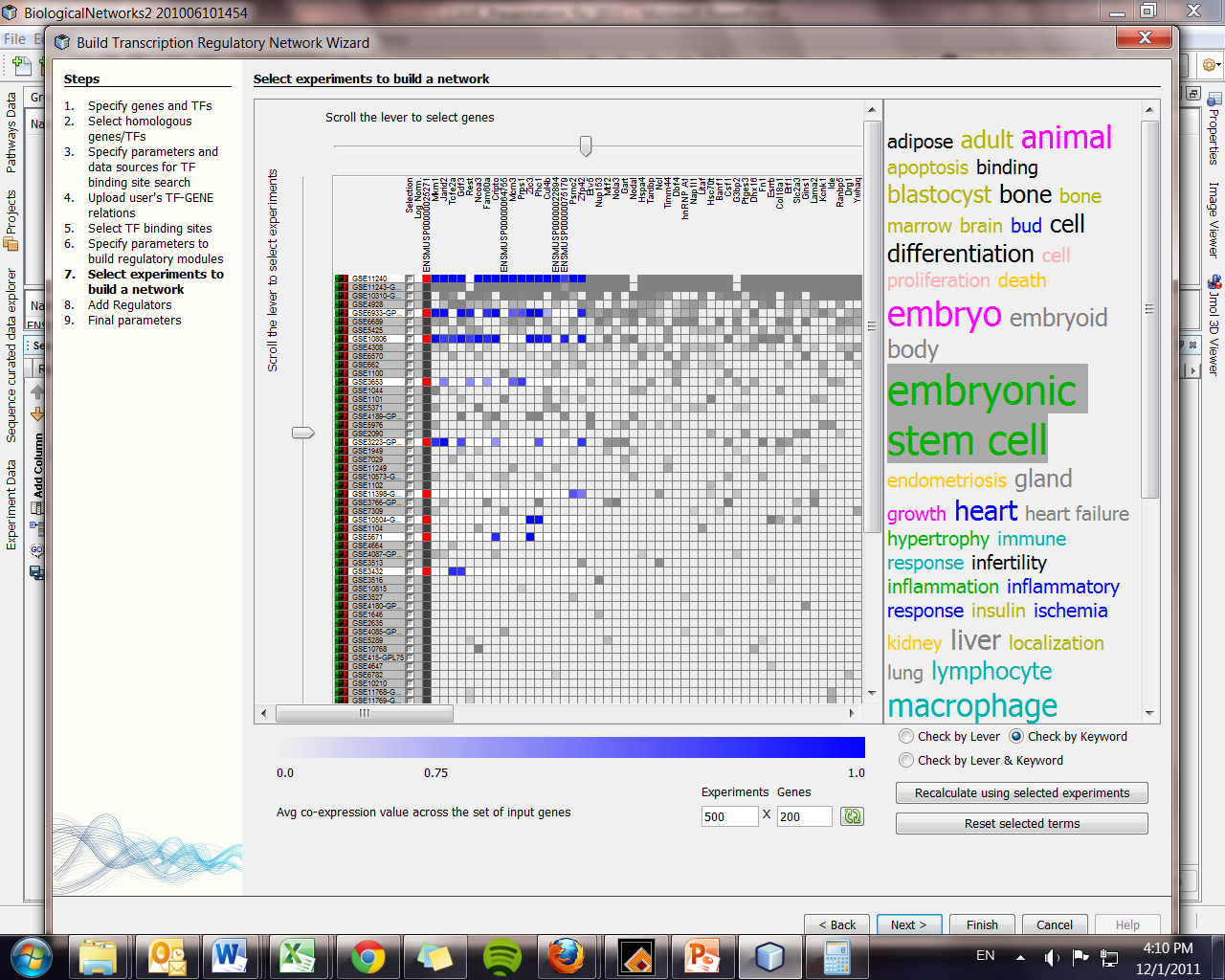
**
- **Select microarray experiments (continue).** Move the upper lever to the far right to select 200 shown genes (more genes can be shown by changing the number at the bottom and clicking the recalculation button). Move the left lever to select 5 top experiments. Click ‘Next’.
- **
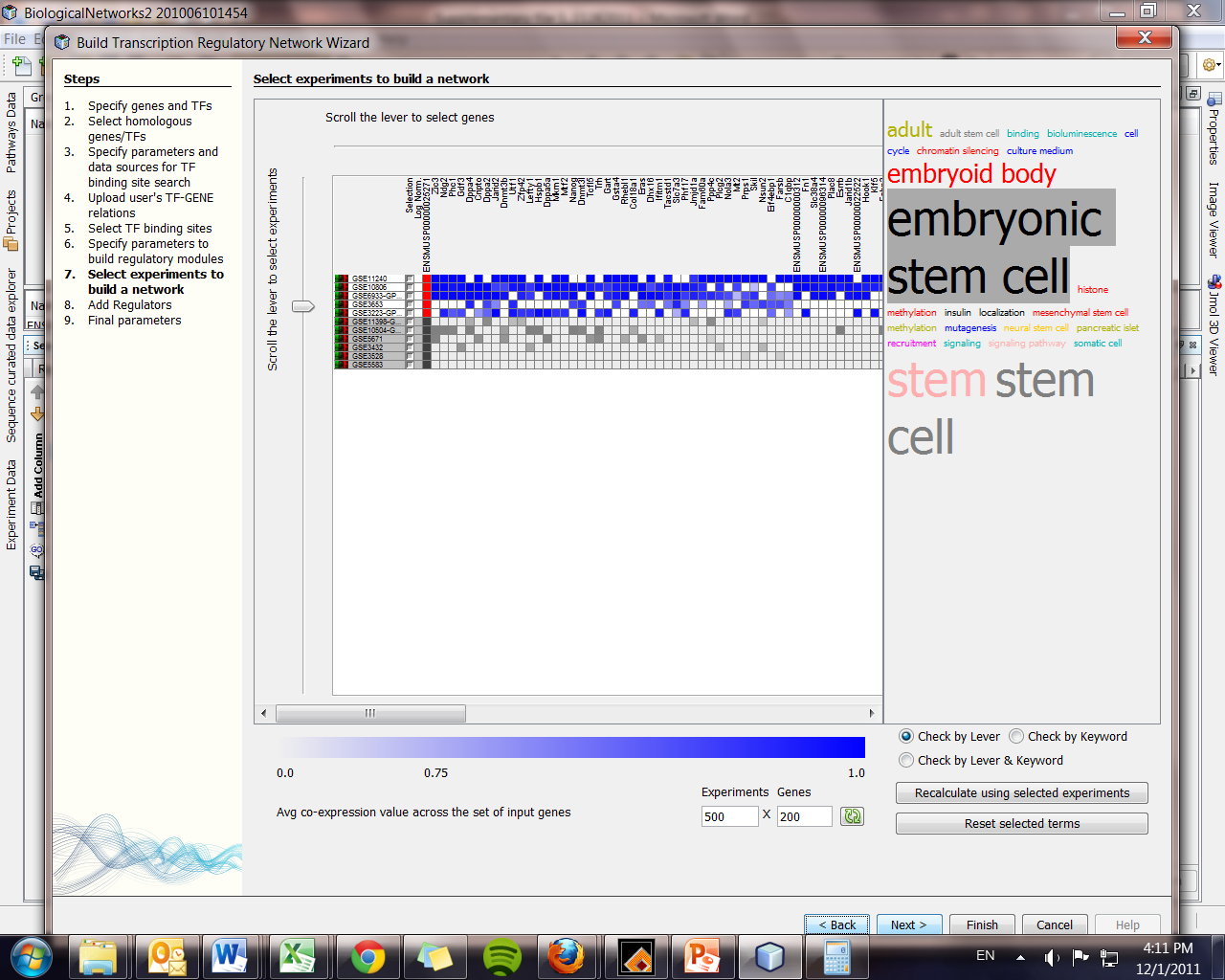
**
- **Select/unselect regulators. Unselect regulators.
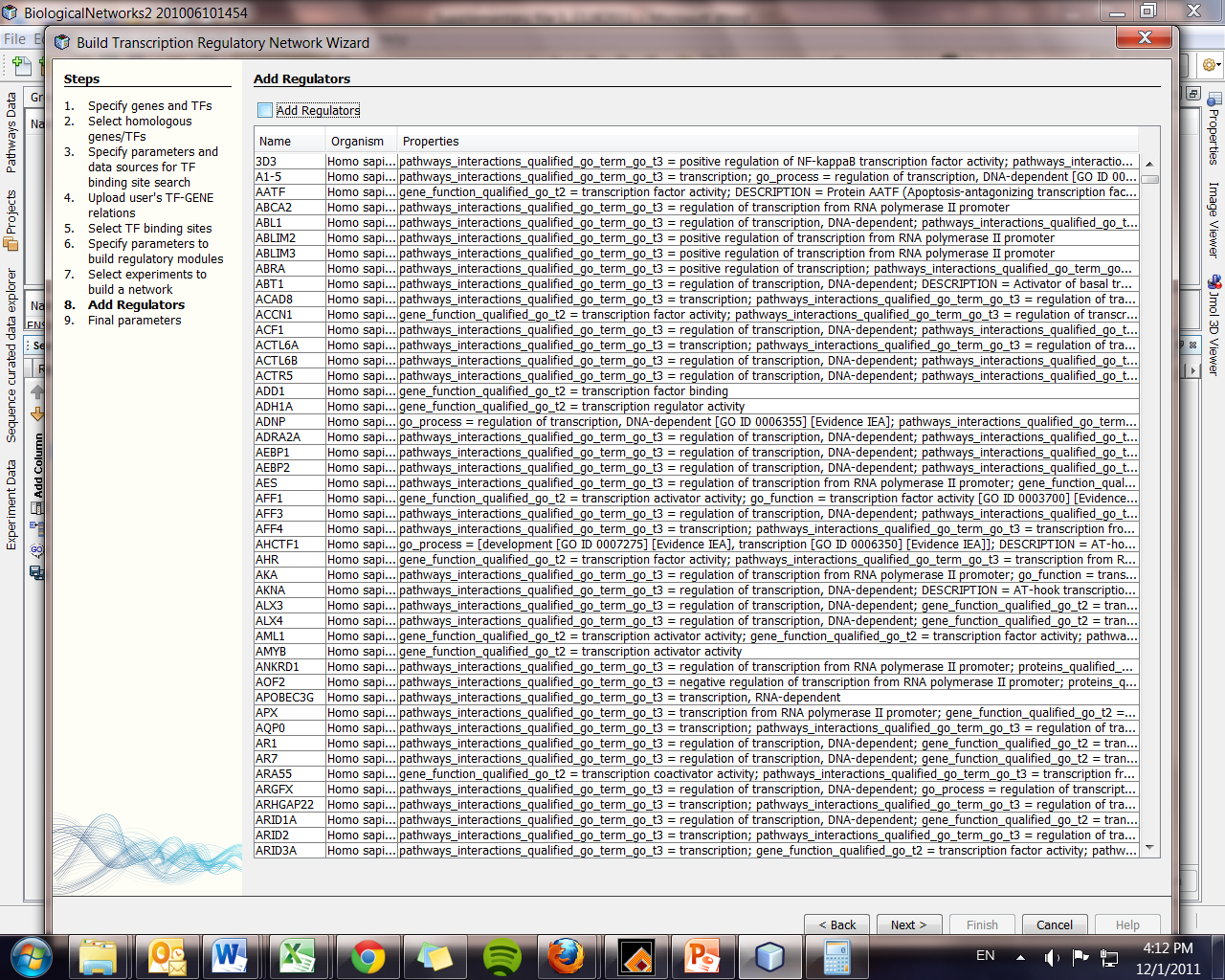
**
- **Select/unselect ‘Create Gene Regulatory Network’.** Use default (select ‘Create Gene Regulatory Network’). Click ‘Finish.’ At this point, the Wizard finishes its work, and the modules will start to be infered. The results are shown in Figure 2 of the main text.

**
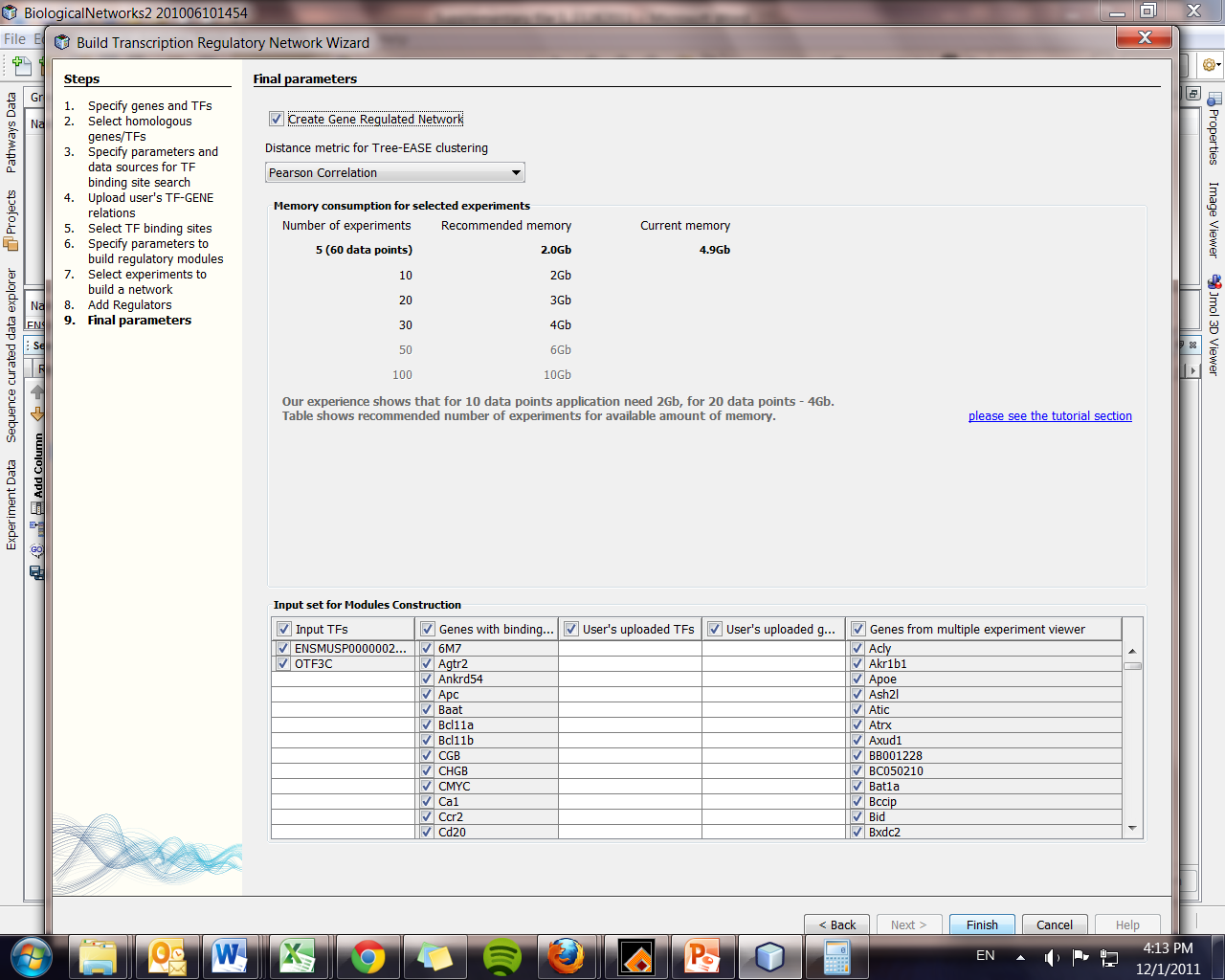
**

In the final Integrative View click “Integrative View” button on the Toolbar so that the windows become

synchronized and data and results could be explored synchronically in different windows.
