# Supplementary material for: An Integrative Approach to Inferring Gene Regulatory Module Networks
Source: PLoS One. 2012 Dec 20;7(12):e52836. doi: 10.1371/journal.pone.0052836 (PMC3527610; doi:10.1371/journal.pone.0052836)
Supplement: File S2 — How to compare two lists of genes/proteins in BiologicalNetworks. (DOCX) [file pone.0052836.s002.docx]

**Supplementary file S2. How to compare two lists of genes/proteins**

- On the right top corner of BiologicalNetworks click ‘…’ button (‘Select text file’) to upload the list of IDs from the file. To compare the list of genes in the modules with the list of OCT4 targets from, for example, Sharov et al, first, upload **Supplementary file S10** that contains list of genes in 70 top modules obtained in Study1 (in the screen-shot below this file is called ‘Study1_70top_modules_genes’).
- **
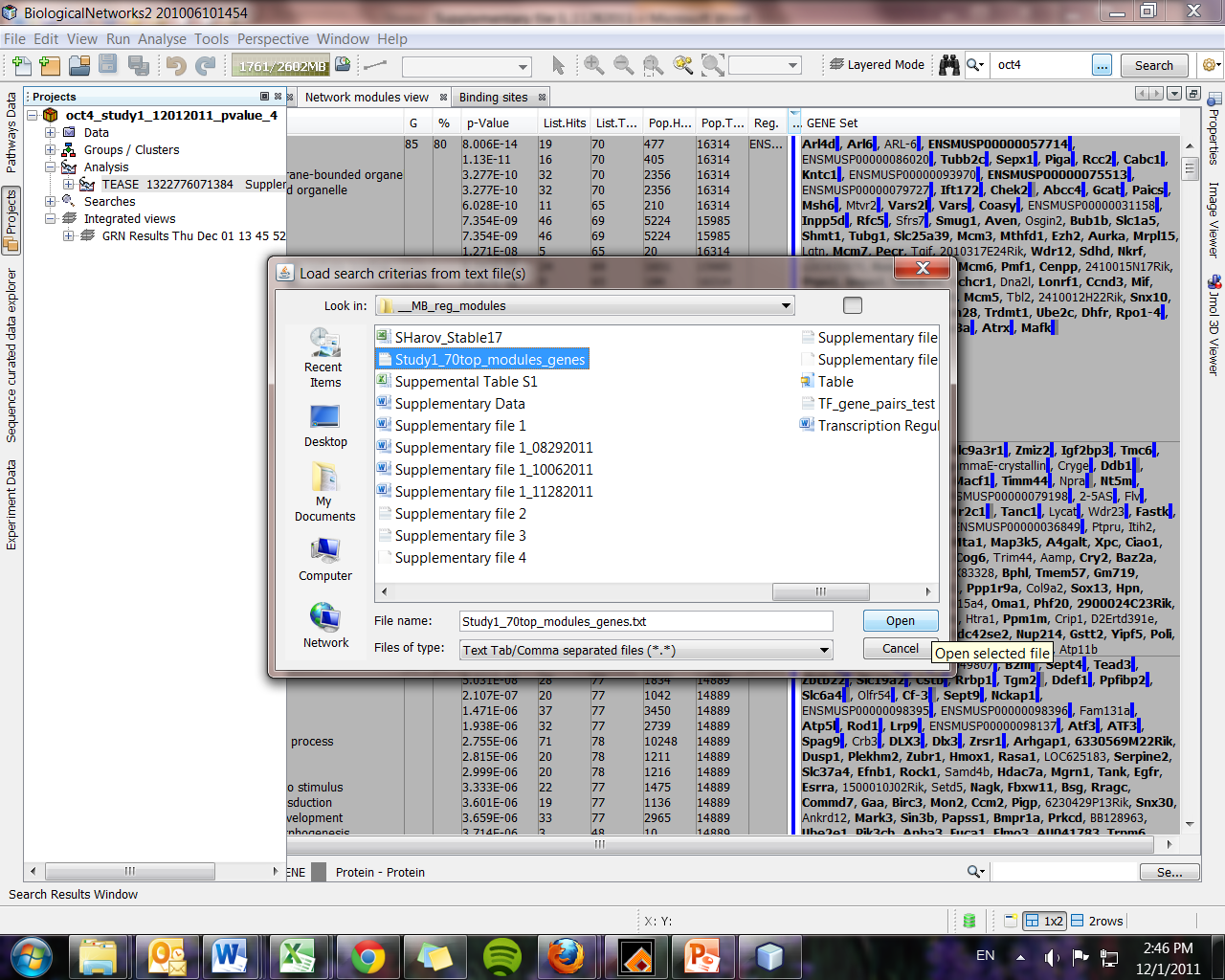
**
- **Select the organism ‘Mus musculus’ (in the right top coner), and click ‘Search’ to search for these genes in IntegromeDB. If invisible, the window with search results can be brought back.
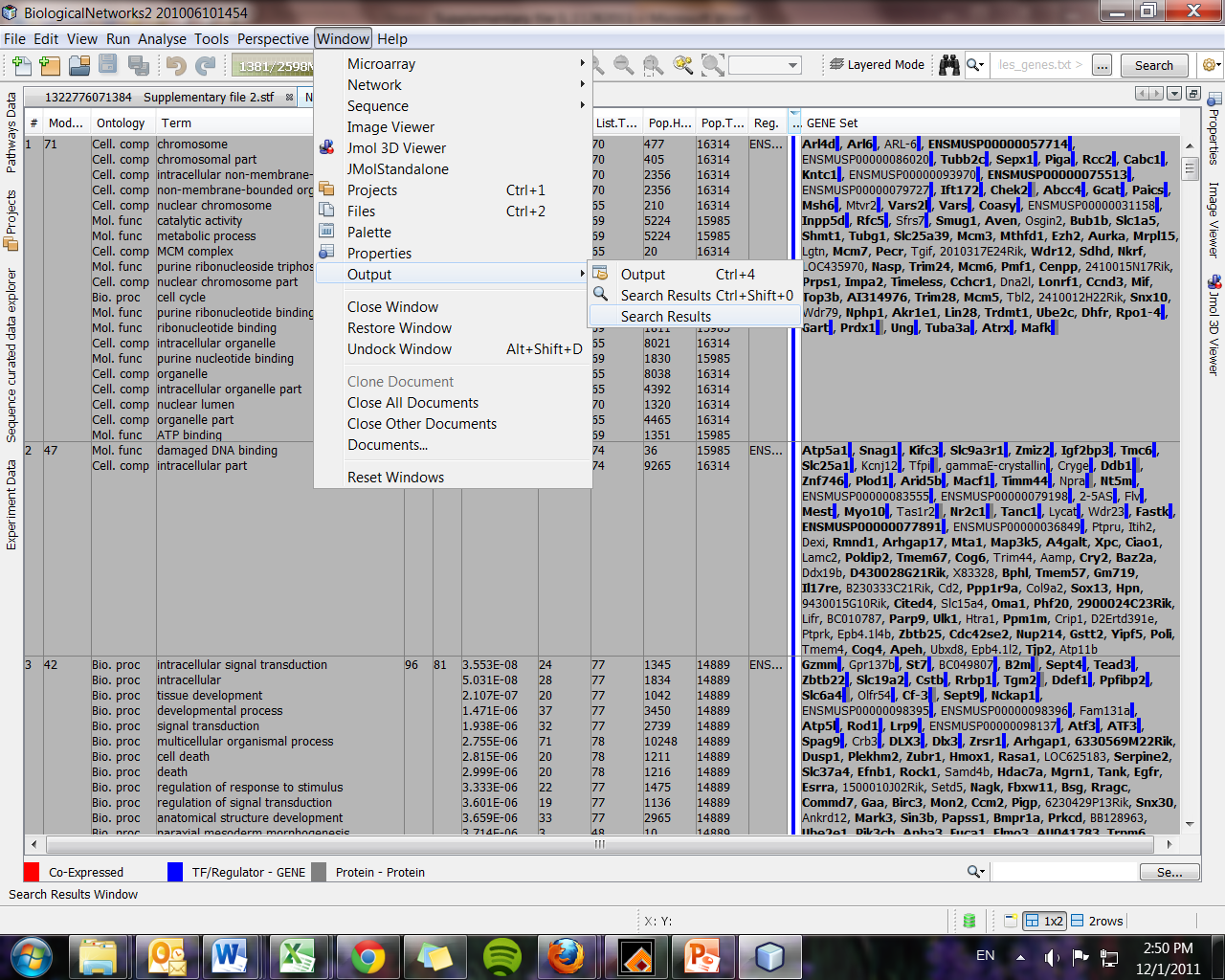
**
- **After search is done, expand the list and click on ‘Type’ tab to sort found entities by type.
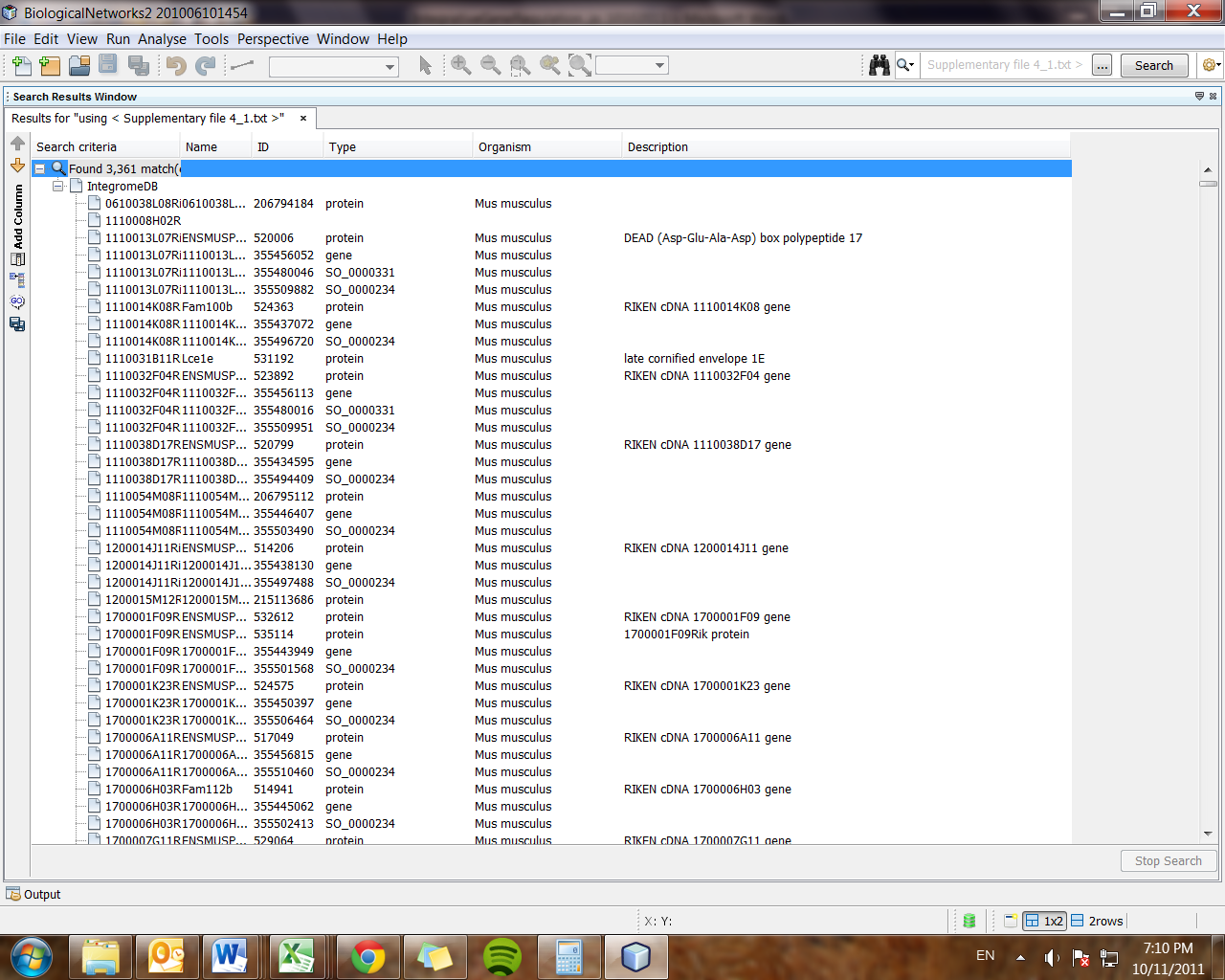
**
- **Select proteins** since they have more synonyms in different databases than genes and we don’t want to count the same gene twice as a protein and a gene. Click twice on ‘Type’ to sort the list in ‘Z to A’ order. Select all proteins, click right mouse button, and click ‘Store Cluster’ to store the selected proteins as a cluster. **
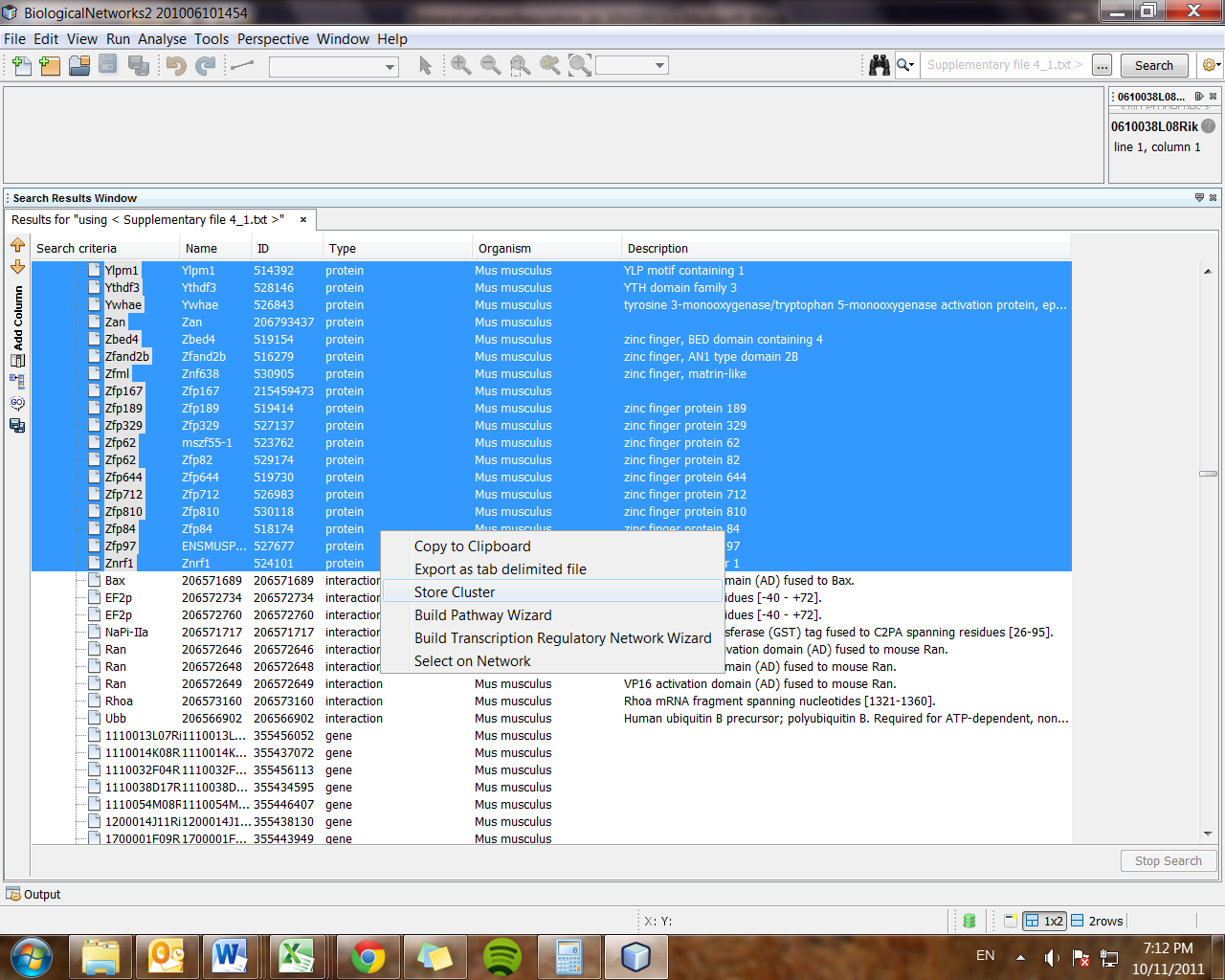
**

**
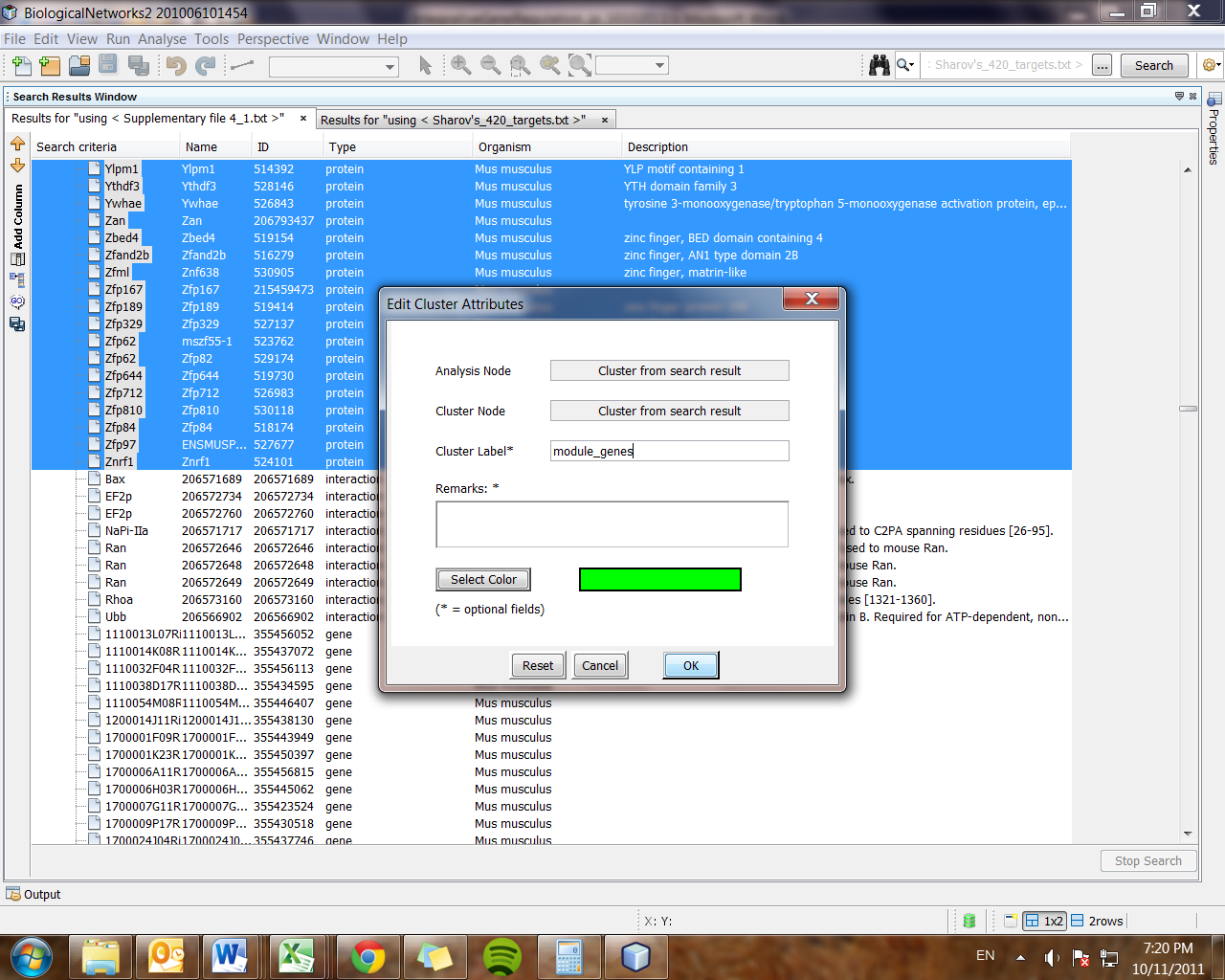
**

- **Upload the second list of genes/proteins which will be compared with the first one.** For example, upload the list of 420 genes (OCT4 targets) from Sharov’s Add. File 17 (**Supplementary file S11**; in a screen-shot below it is called ‘Sharov_420_targets). Click ‘Search’.**
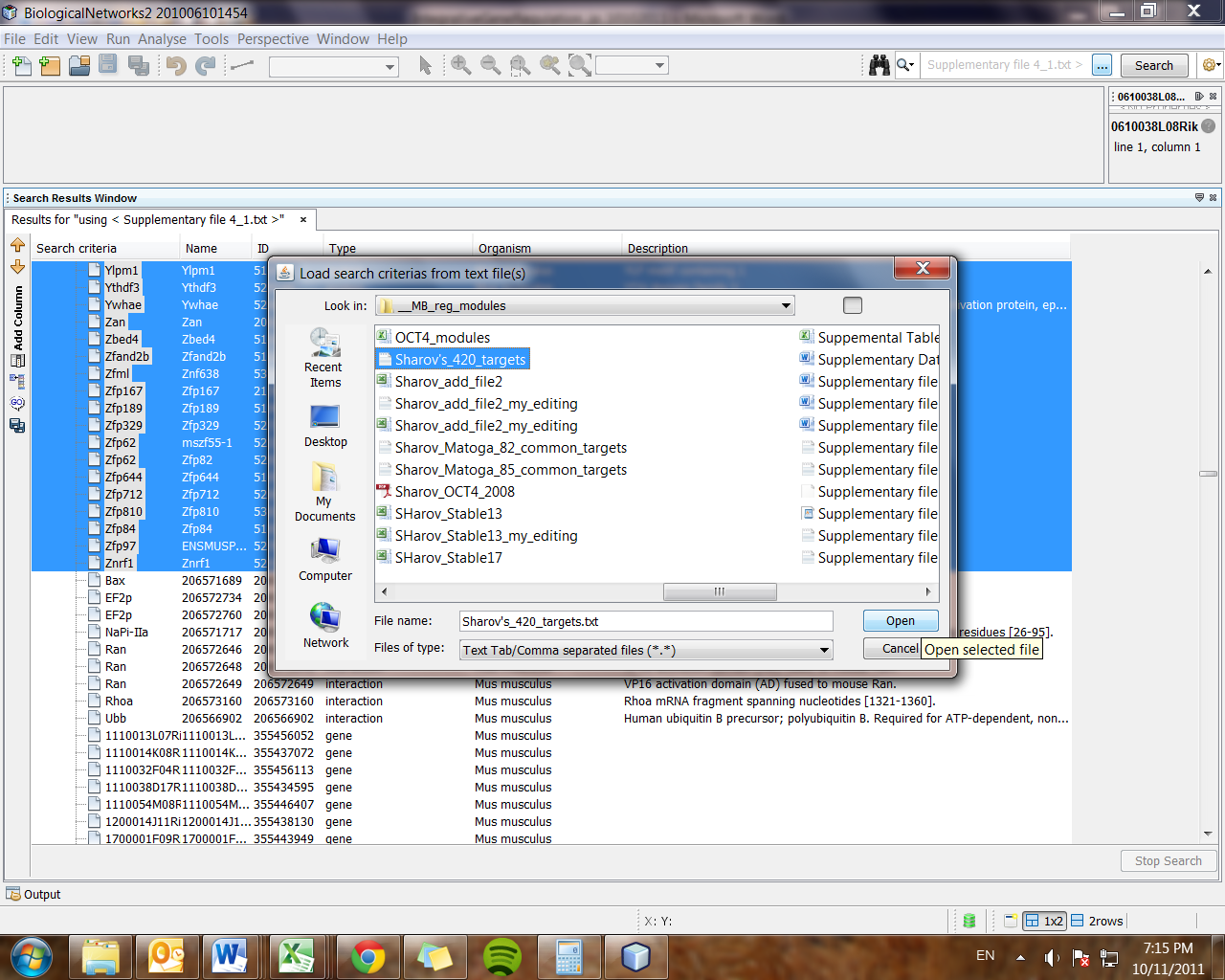
**
- **Select proteins** since they have more synonyms in different databases than genes and we don’t want to count the same gene twice as a protein and a gene. Click twice on ‘Type’ to sort the list in ‘Z to A’ order. Select all proteins, click right mouse button, and click ‘Store Cluster’ to store the selected proteins as a cluster. **
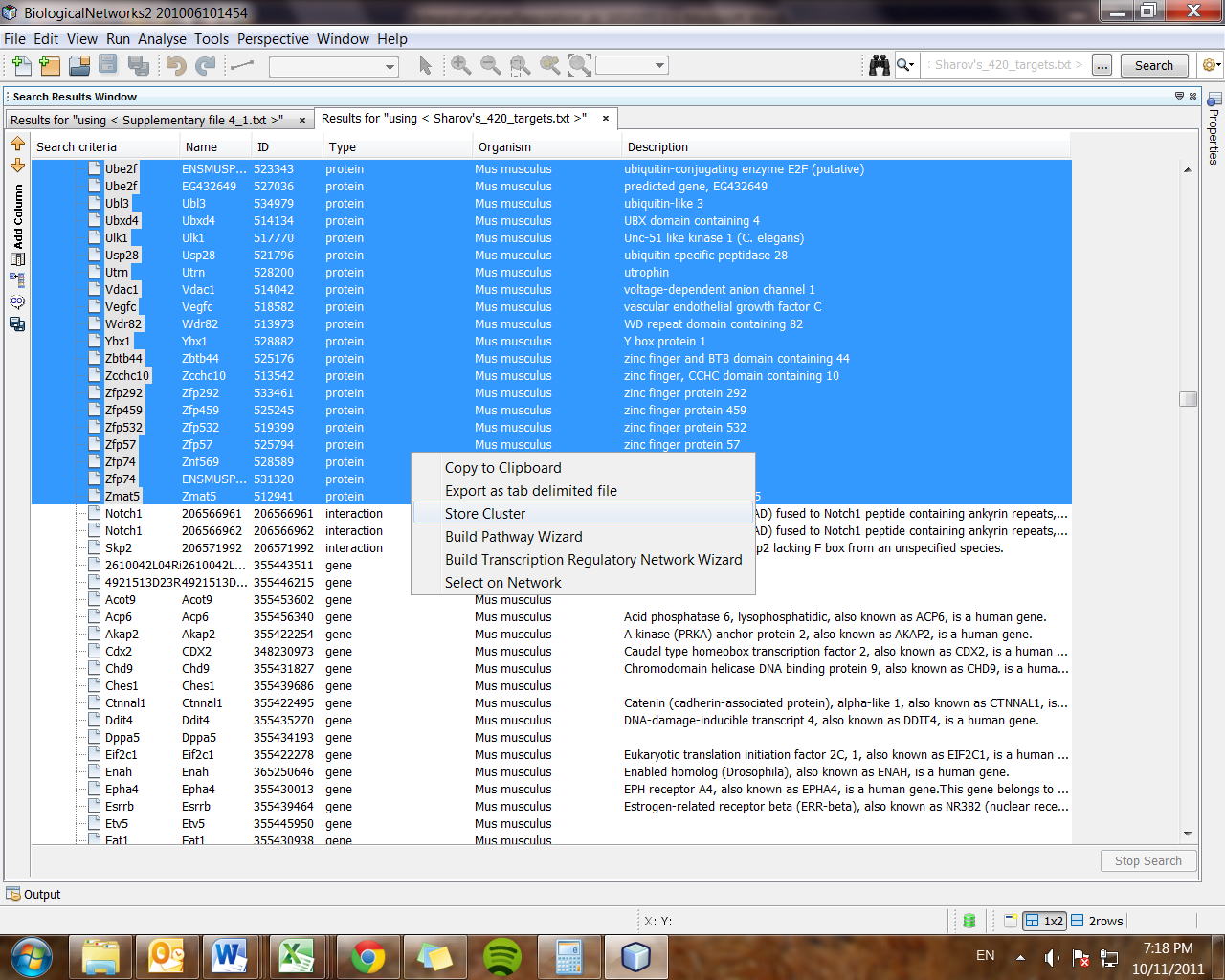
**

**
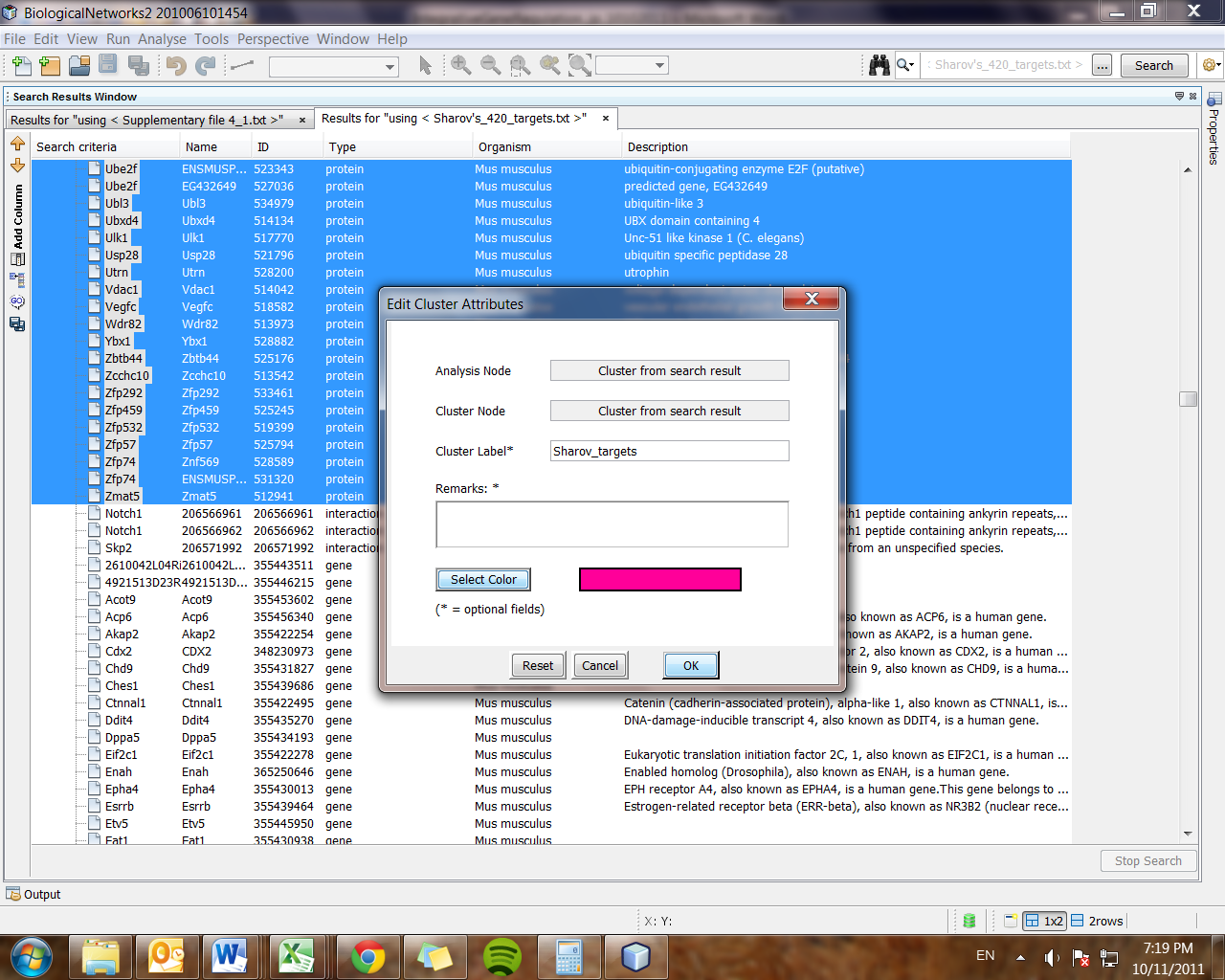
**

- In the project window, click on Groups/Clusters to see the clusters. Select the two clusters to compare in the window where they are shown and using the right mouse button, select ‘Cluster Operation -> Intersection’. **
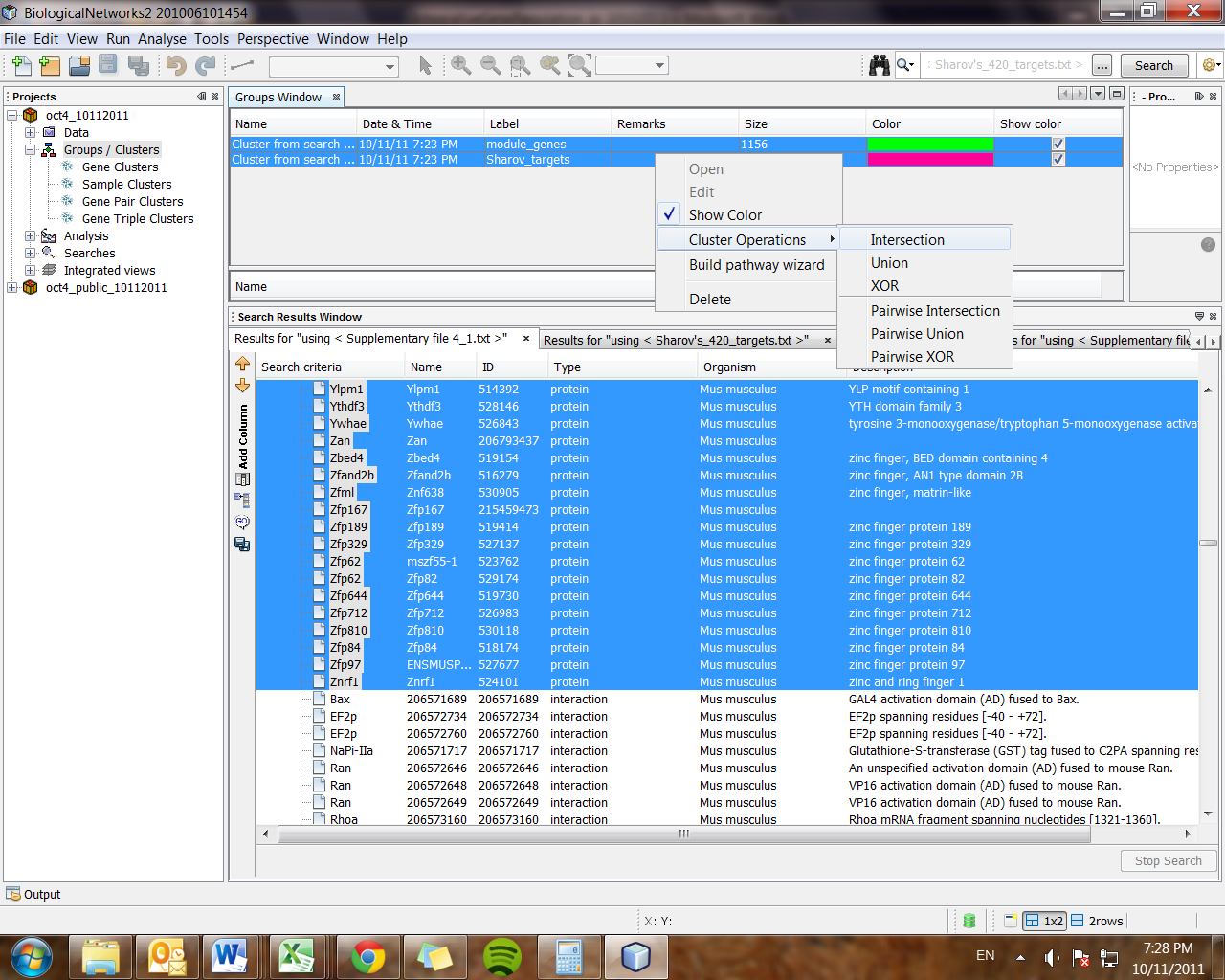
**

**
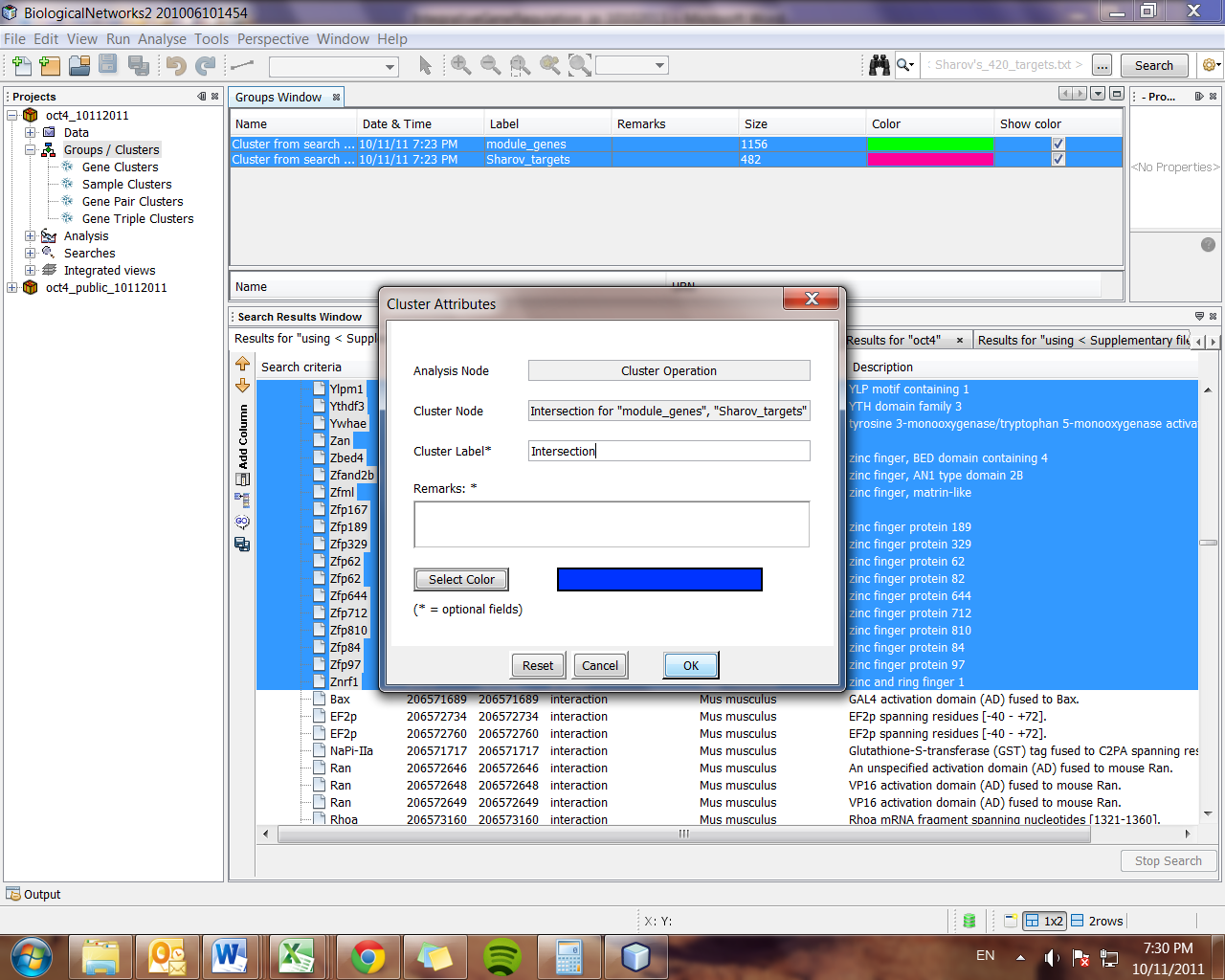
**
