# Supplementary material for: An Integrative Approach to Inferring Gene Regulatory Module Networks
Source: PLoS One. 2012 Dec 20;7(12):e52836. doi: 10.1371/journal.pone.0052836 (PMC3527610; doi:10.1371/journal.pone.0052836)
Supplement: File S3 — Step-by-step analysis for Use Case #2. (DOCX) [file pone.0052836.s003.docx]

**Supplementary file S3.**

- [**Use Case #2. Gene regulatory network of Asthma. Study 1.**](#asthma)
- [**The result of study. Use Case #2, Study 2. Integrative view of Module 1.**](#uc2_st2)

**Note: To run Study 1 in BiologicalNetworks, 10 GB RAM is required.**

**Use Case #2. Gene regulatory network of Asthma. Study 1.**

- At the top right corner of BiologicalNetworks select Mus musculus as an organism (click the lens icon button). Click the ‘…’ button to upload the files with the list of genes. The files listed genes in all 61 modules discovered by the authors were downloaded at <http://www.jail.cs.huji.ac.il/~shefi/> and uploaded in BiologicalNetworks.


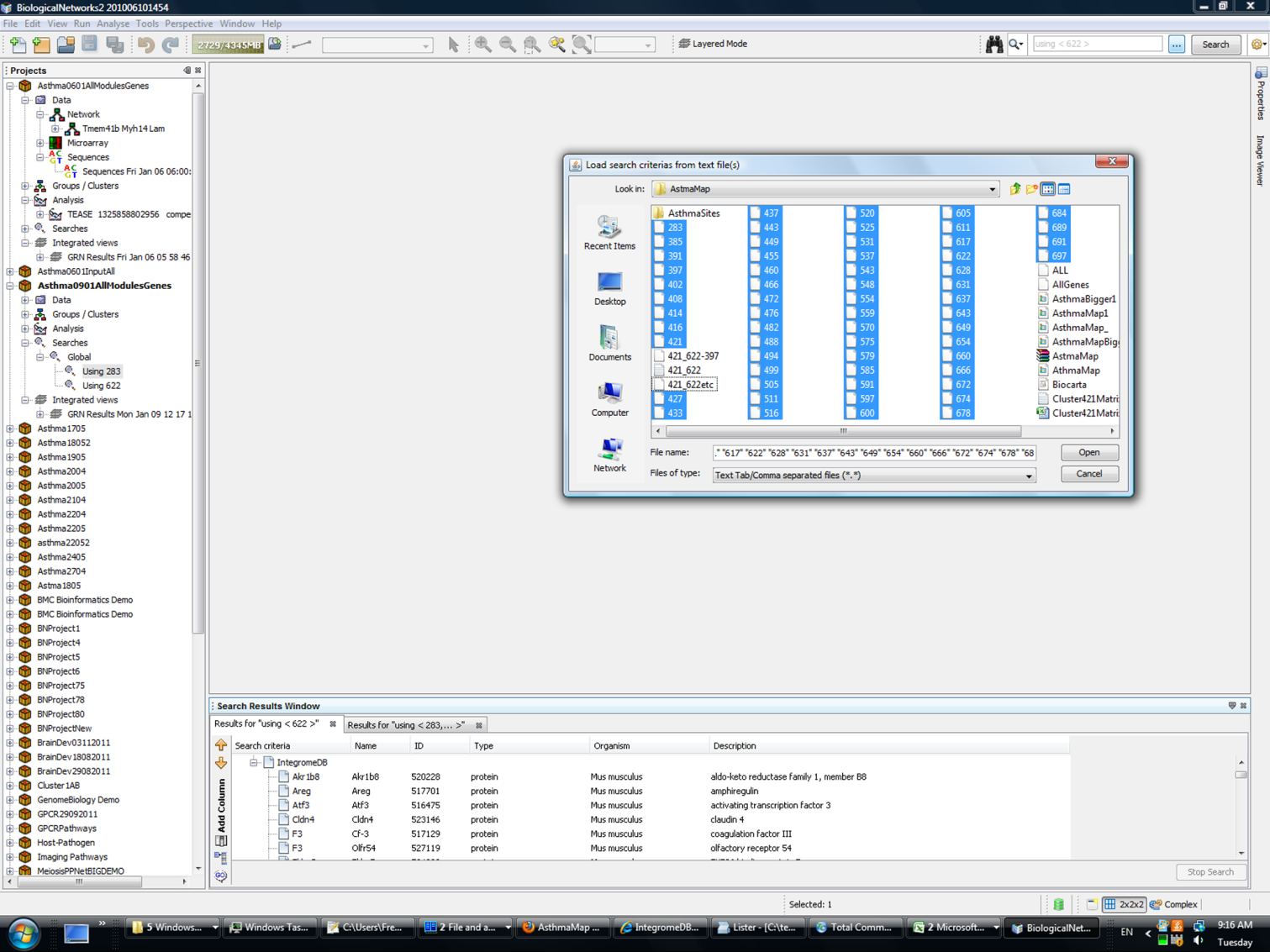


- In the search results panel select all proteins (there were 6890 proteins found) and using the right mouse click select ‘Build Transcription Regulatory Network Wizard’.


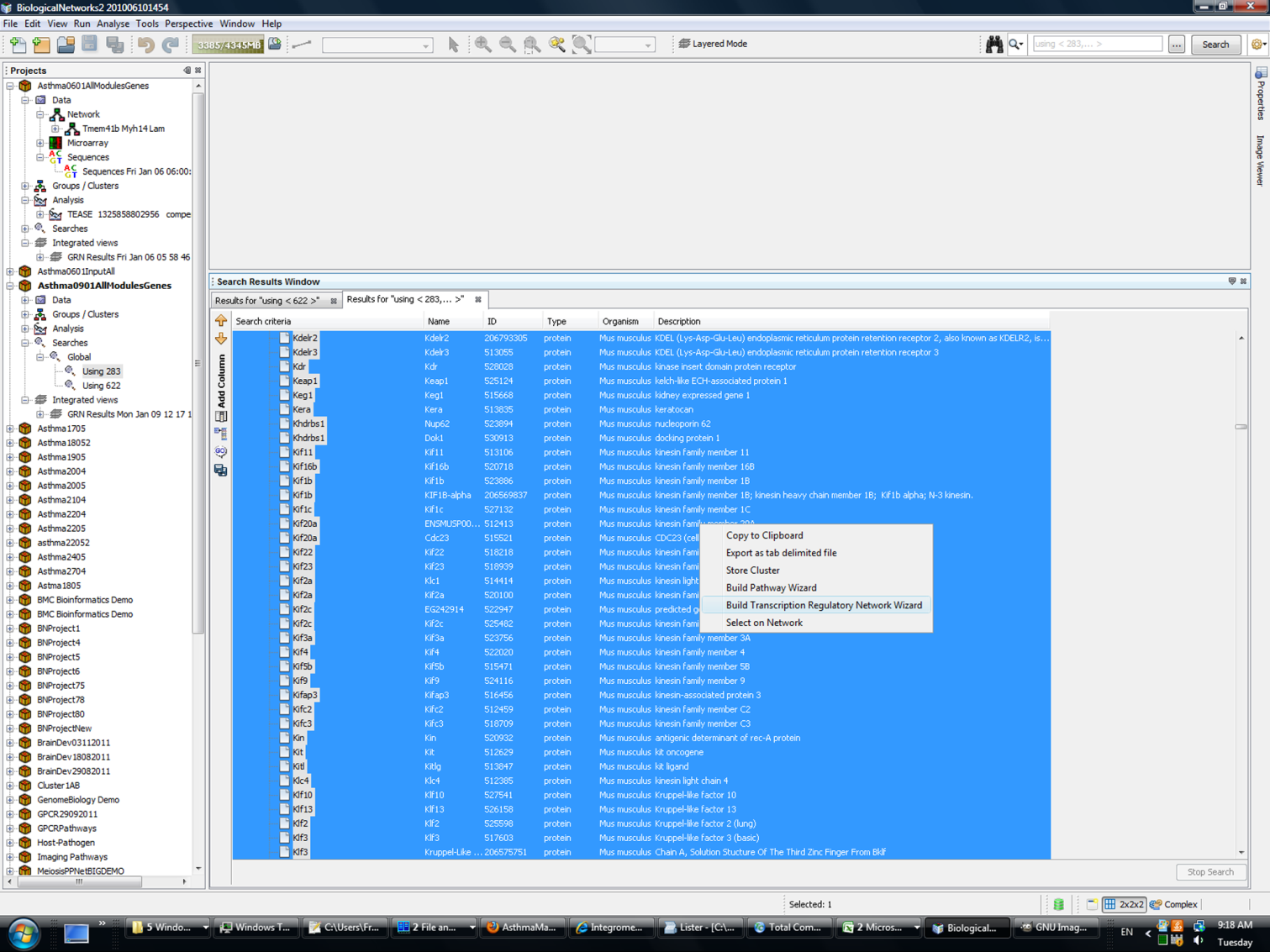


- **Specify genes and TFs. Select ‘**Skip homologous search’ as the network will be built for mouse only. Select ‘Genes’ for the input list as we are interested in TFs that regulate these genes. Click “Next”.


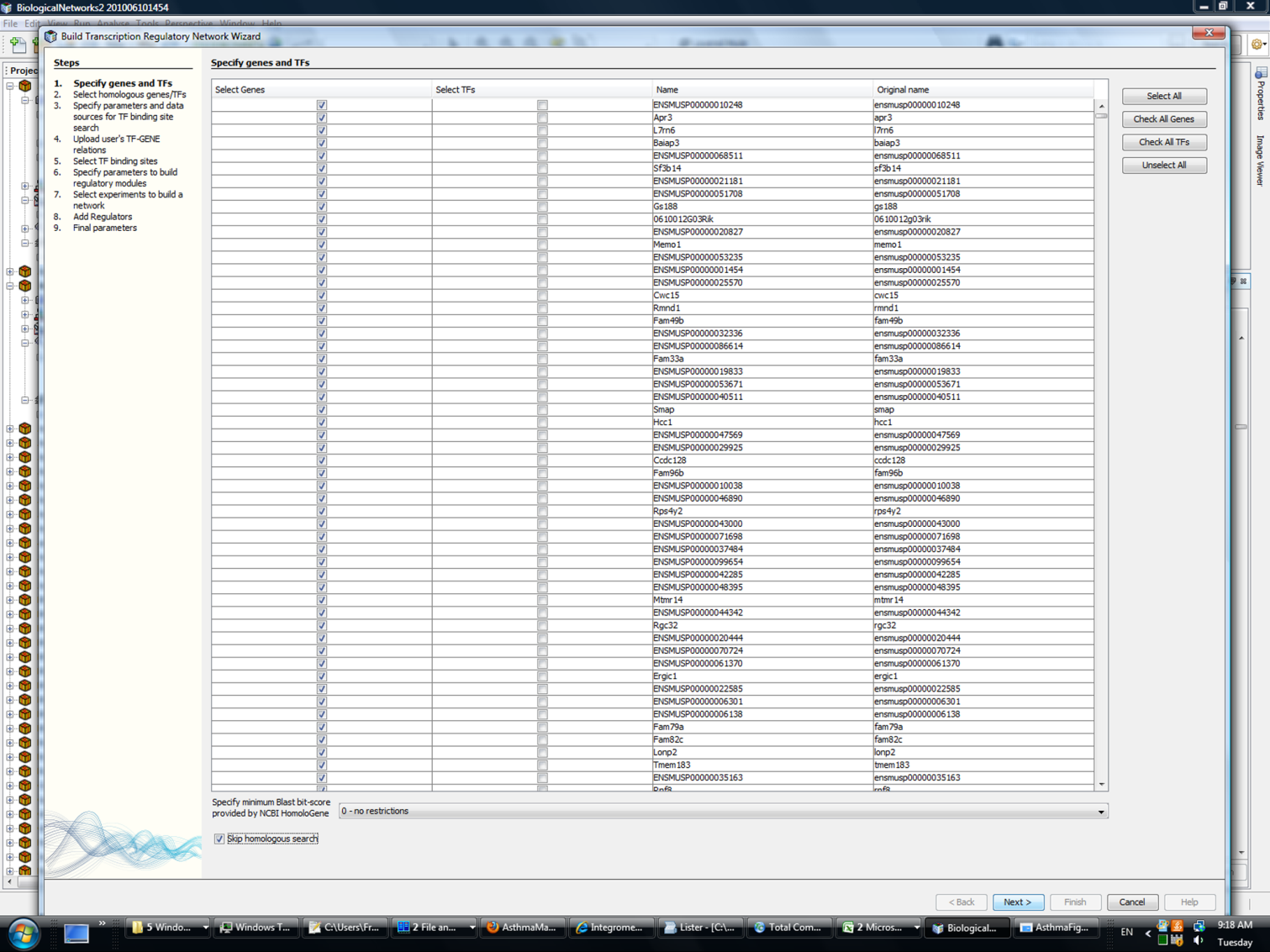


- **Specify parameters for TF binding sites search.** Select all data sources. Select 10,000 bp for upstream and 1000 bp for downstream regulatory region. Click ‘Next.’


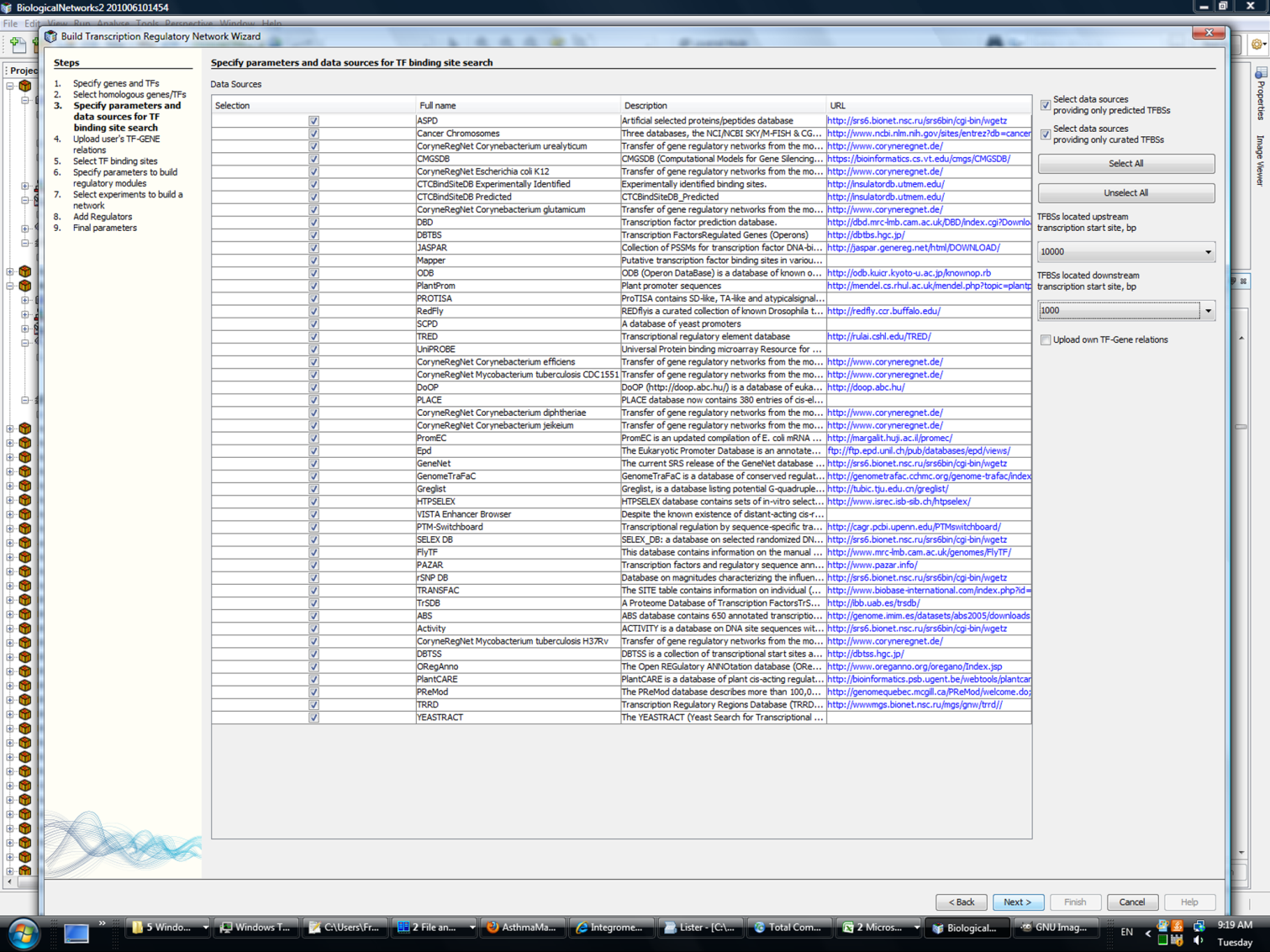


- **Select TF binding sites.** By default all found sites, TFs and genes are selected. Use this default. Make sure that Mus musculus is specified as a main organism. Click ‘Next.’


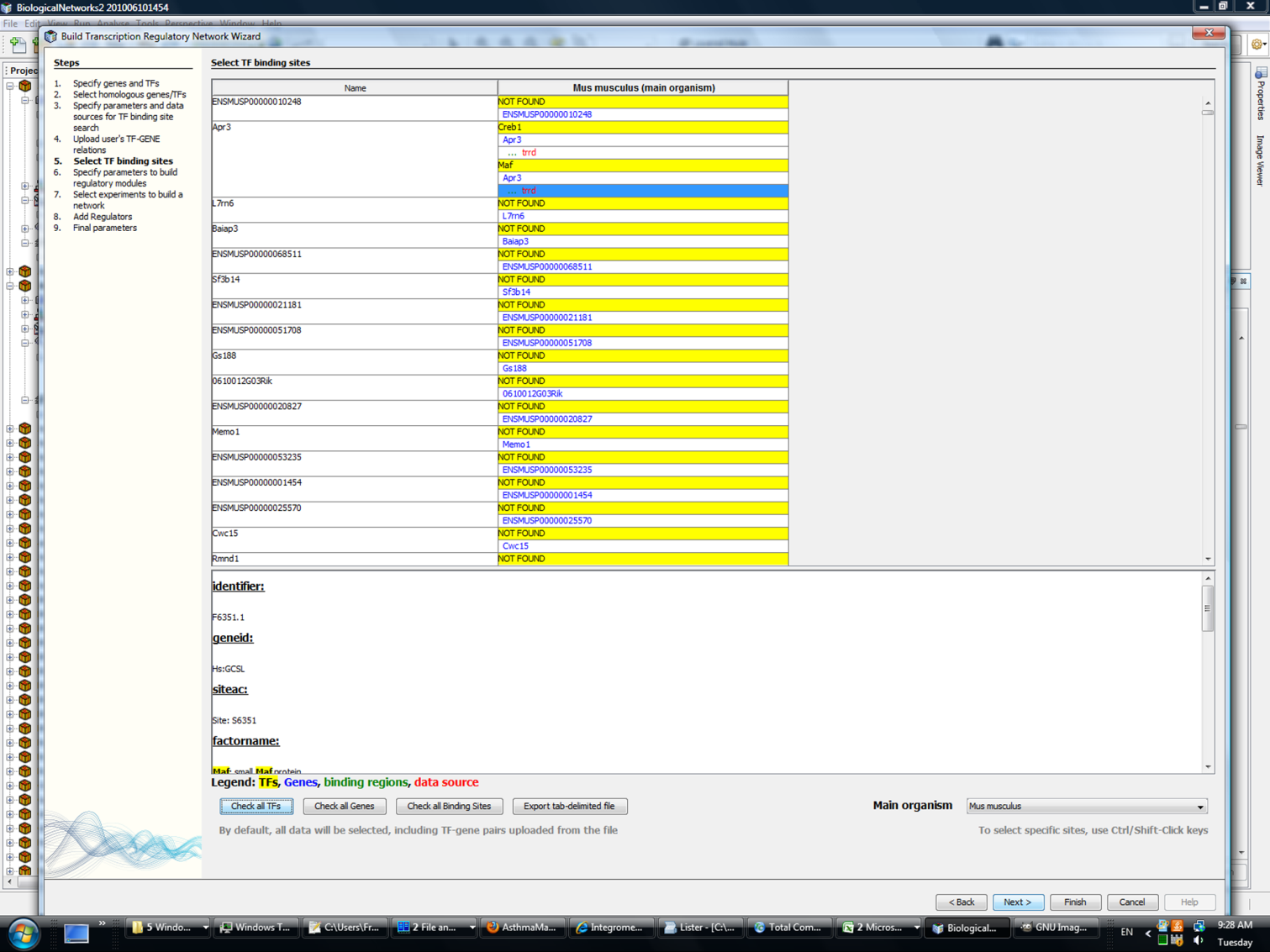


- **Specify parameters to build regulatory modules.** Specify p-value at 0.001 (1.0E-3), select *‘User’s data’* for the source of microarray experiments, and upload microarray data from the file ‘Compendium_2’ provided at <http://www.jail.cs.huji.ac.il/~shefi/>). Click ‘Next.’


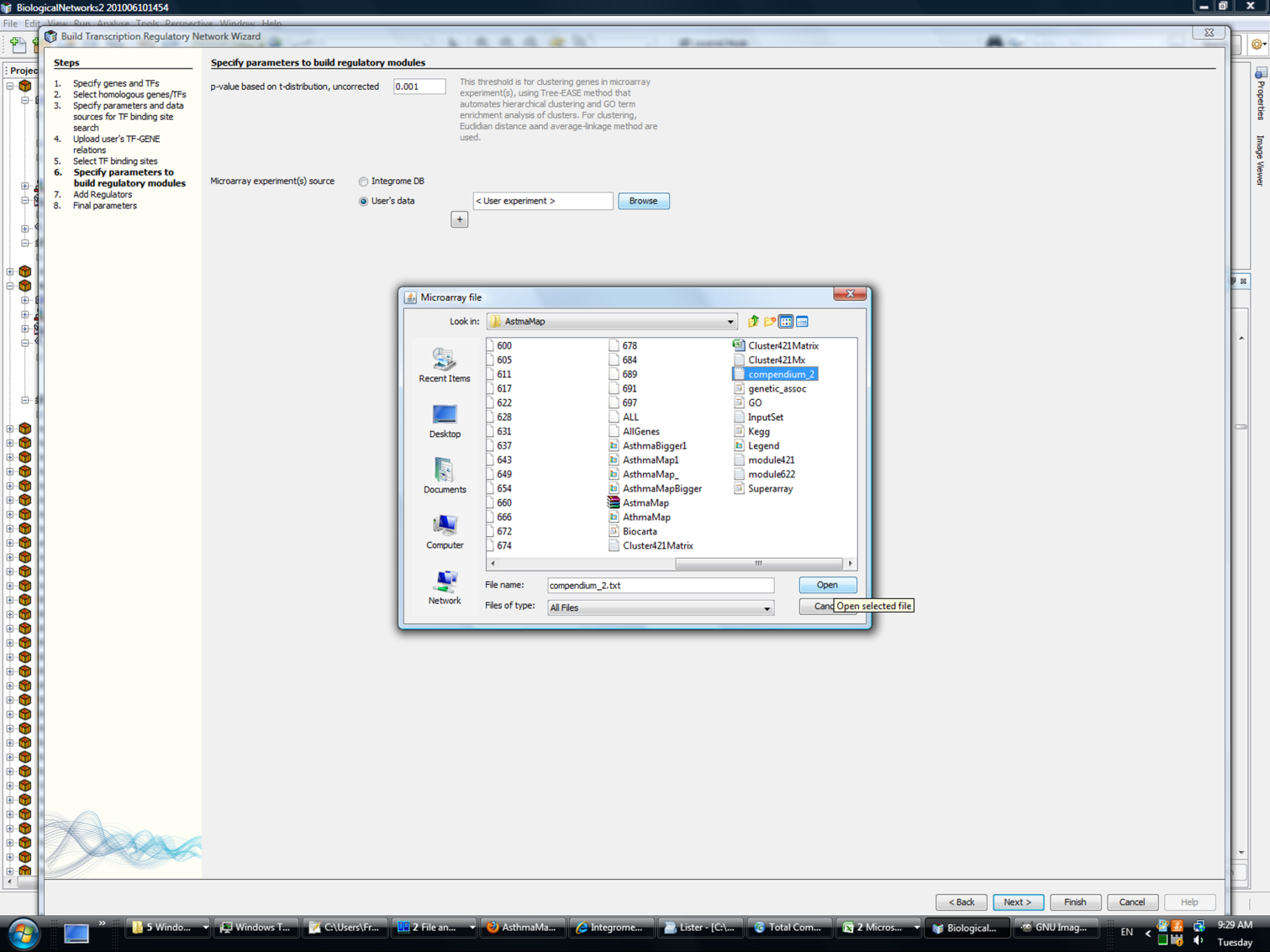


- **Select/unselect regulators. Select** ‘Add Regulators’. Click ‘Next’.


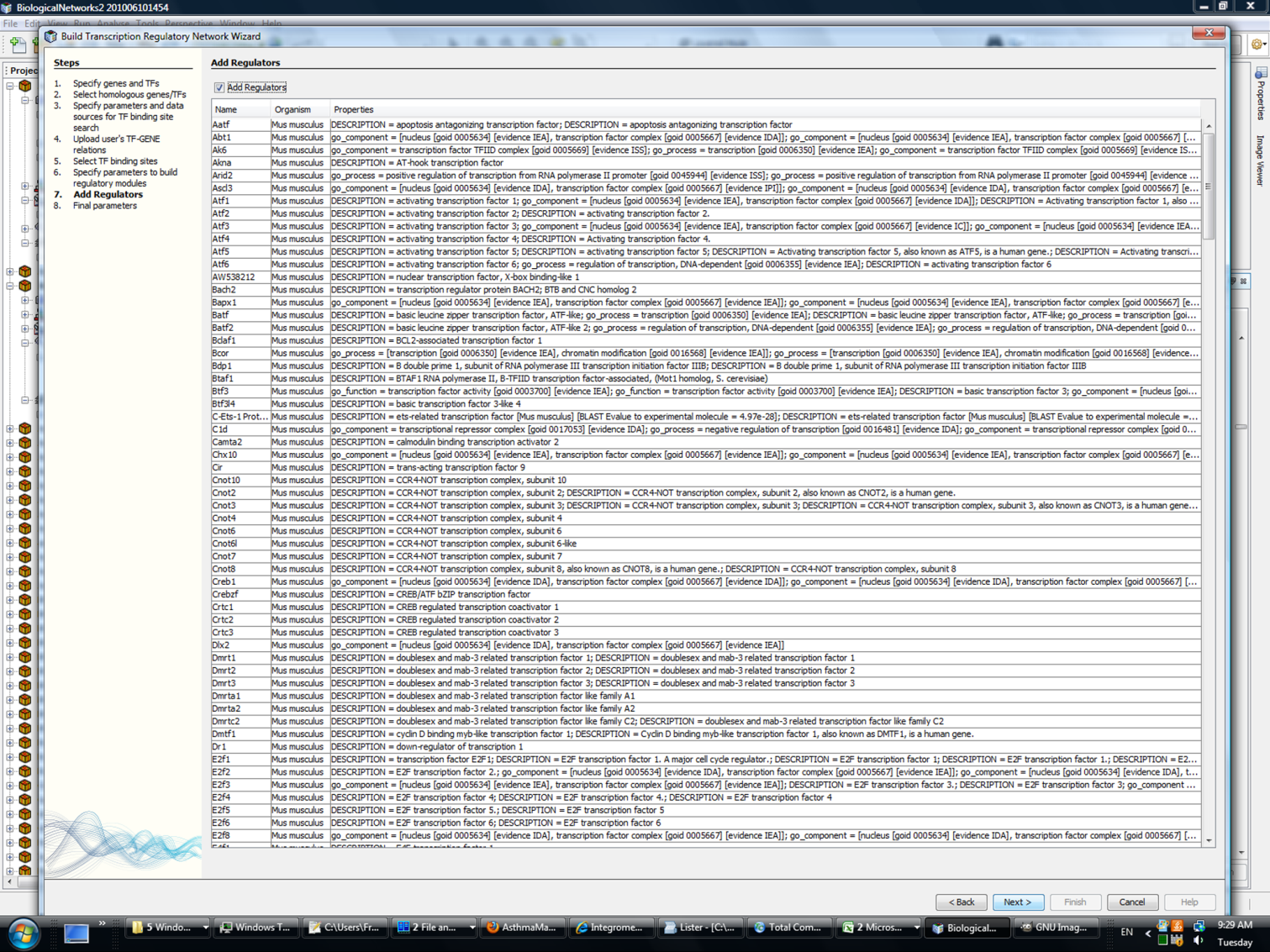


- **Final parameters.** Select ‘Create Gene Regulatory Network’. Also, select Pearson correlation as a distance for clustering and click ‘Finish’.


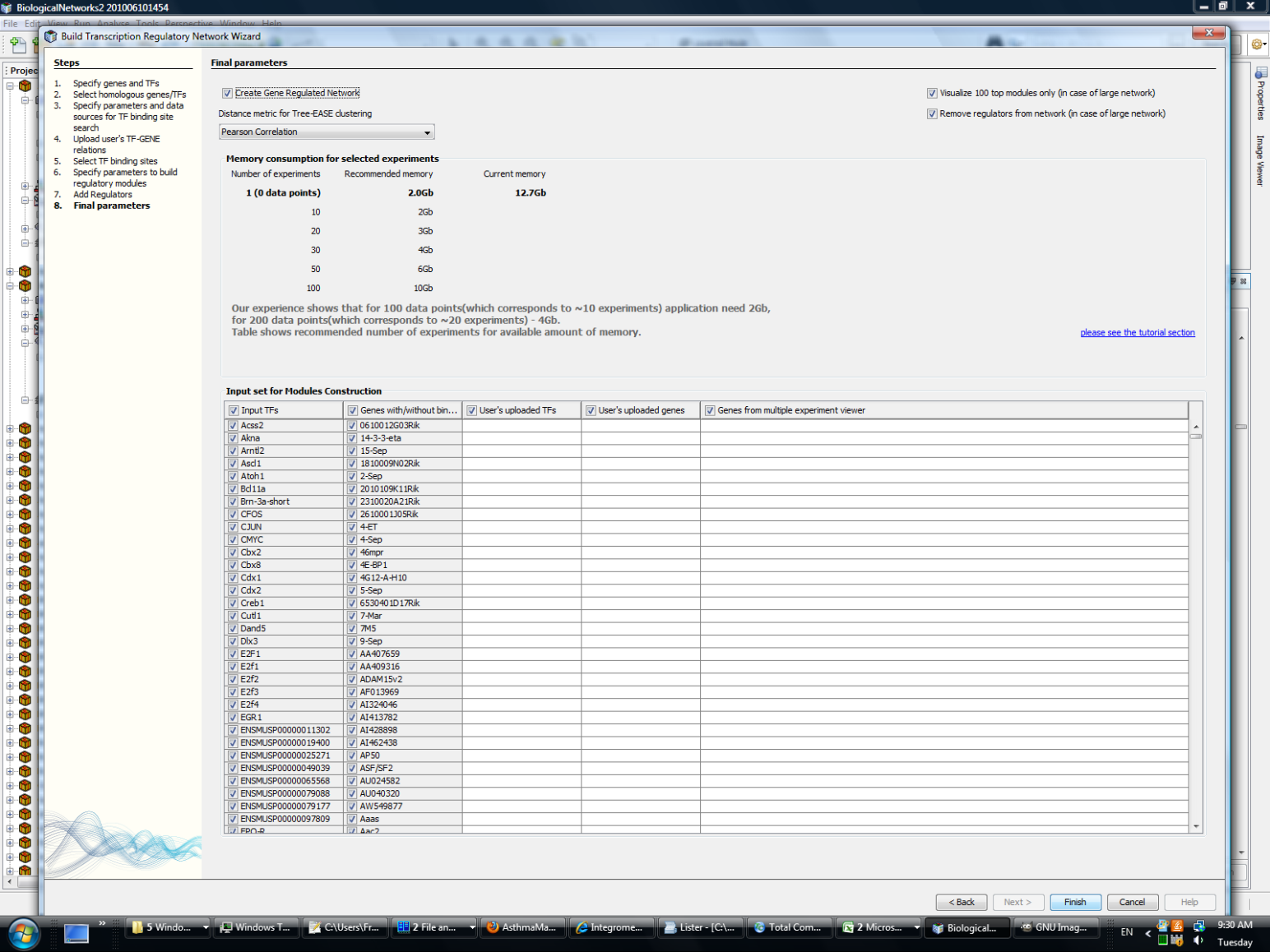


**The result of study. Use Case #2, Study 2. Integrative view of Module 1.**

**
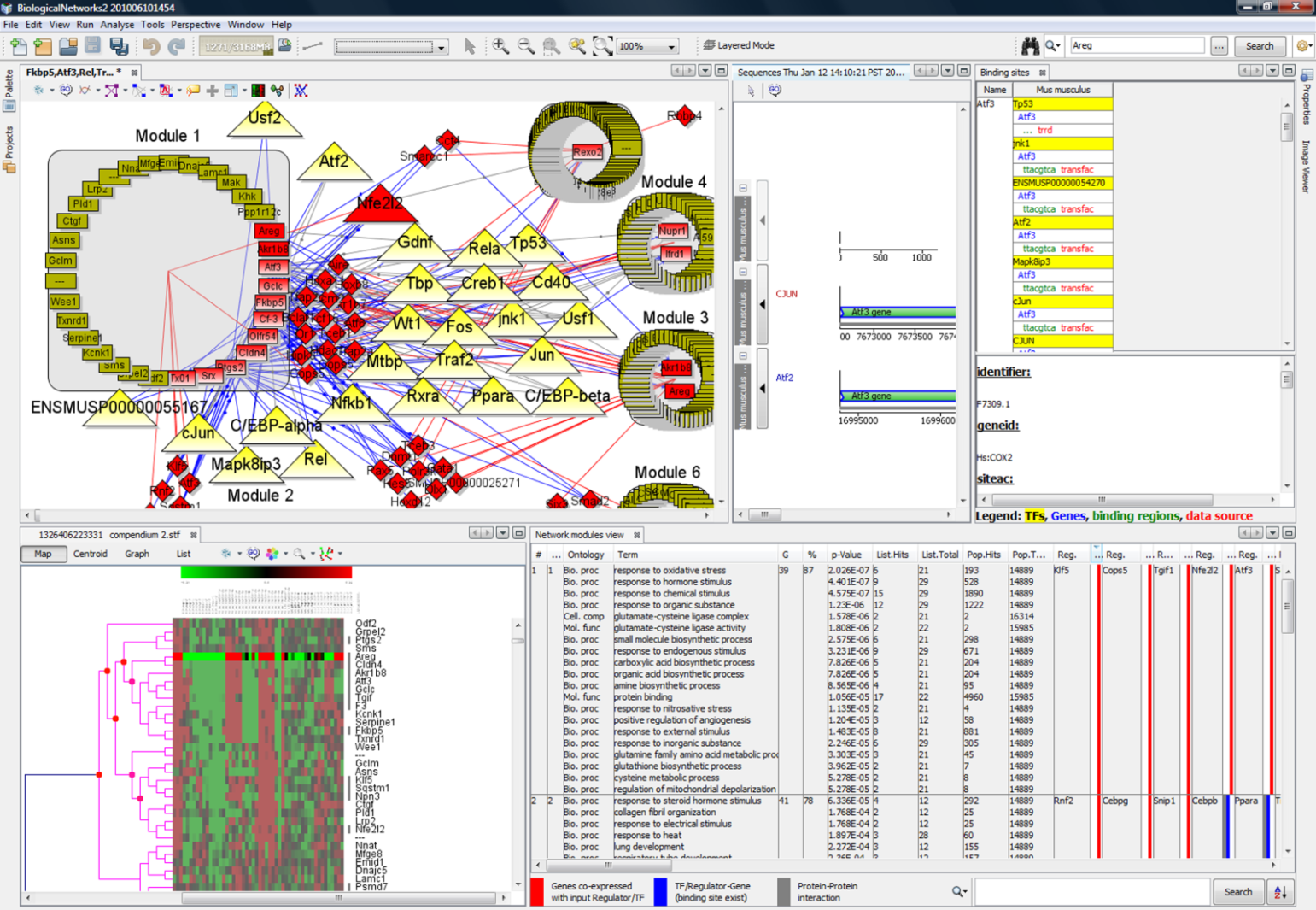
**

In the final Integrative View click “Integrative View” button on the Toolbar so that windows become

synchronized and to explore data synchronically in different windows
